# Effectiveness of system navigation programs linking primary care with community-based health and social services: a systematic review

**DOI:** 10.1186/s12913-023-09424-5

**Published:** 2023-05-08

**Authors:** Kylie Teggart, Sarah E. Neil-Sztramko, Abbira Nadarajah, Amy Wang, Caroline Moore, Nancy Carter, Janet Adams, Kamal Jain, Penelope Petrie, Aref Alshaikhahmed, Shreya Yugendranag, Rebecca Ganann

**Affiliations:** 1 School of Nursing, Faculty of Health Sciences, McMaster University, 1280 Main St. W, Hamilton, ON HSC 3N25L8S 4K1 Canada; 2 Department of Health Research Methods, Evidence and Impact, Faculty of Health Sciences, McMaster University, 175 Longwood Rd S, Suite 210a, Hamilton, ON L8P 0A1 Canada; 3 Department of Family Medicine, University of Alberta, 5-16 University Terrace, Edmonton, AB T6G 2T4 Canada

**Keywords:** Health services research, Patient and public involvement, Patient navigation, Primary care, Social prescribing, Social services, System navigation, Systematic review

## Abstract

**Background:**

Fragmented delivery of health and social services can impact access to high-quality, person-centred care. The goal of system navigation is to reduce barriers to healthcare access and improve the quality of care. However, the effectiveness of system navigation remains largely unknown. This systematic review aims to identify the effectiveness of system navigation programs linking primary care with community-based health and social services to improve patient, caregiver, and health system outcomes.

**Methods:**

Building on a previous scoping review, PsychInfo, EMBASE, CINAHL, MEDLINE, and Cochrane Clinical Trials Registry were searched for intervention studies published between January 2013 and August 2020. Eligible studies included system navigation or social prescription programs for adults, based in primary care settings. Two independent reviewers completed study selection, critical appraisal, and data extraction.

**Results:**

Twenty-one studies were included; studies had generally low to moderate risk of bias. System navigation models were lay person-led (*n* = 10), health professional-led (*n* = 4), team-based (*n* = 6), or self-navigation with lay support as needed (*n* = 1). Evidence from three studies (low risk of bias) suggests that team-based system navigation may result in slightly more appropriate health service utilization compared to baseline or usual care. Evidence from four studies (moderate risk of bias) suggests that either lay person-led or health professional-led system navigation models may improve patient experiences with quality of care compared to usual care. It is unclear whether system navigation models may improve patient-related outcomes (e.g., health-related quality of life, health behaviours). The evidence is very uncertain about the effect of system navigation programs on caregiver, cost-related, or social care outcomes.

**Conclusions:**

There is variation in findings across system navigation models linking primary care with community-based health and social services. Team-based system navigation may result in slight improvements in health service utilization. Further research is needed to determine the effects on caregiver and cost-related outcomes.

**Supplementary Information:**

The online version contains supplementary material available at 10.1186/s12913-023-09424-5.

## Background

Patients and their caregivers often face significant challenges when navigating increasingly complex health and social services. Frequently left to locate and access these siloed services alone [1], adults living with multifaceted health and social needs have described their care as disjointed, confusing, and uncoordinated [2]. Barriers to accessing available health and social services may include restrictive eligibility criteria and wait lists for services, financial constraints, health literacy and communication challenges, lack of transportation, and poor coordination between primary care providers and health and social service agencies [3]. In an effort to overcome this fragmentation and efficiently access the care they need, patients and caregivers often spend an extraordinary amount of time becoming informal system navigators and de facto care coordinators [4]. This can have significant physical, emotional, social, relational, and financial repercussions [1, 4, 5]. Given the rising prevalence of chronic diseases and multimorbidity worldwide [6], in addition to urgent calls to address the social and structural inequities that exist in health systems [7, 8], identifying effective strategies to support individuals in accessing high-quality health and social care is of vital importance.

Over the last 30 years, system navigation programs have gained popularity globally as a person-centred approach to support individuals to access health and social care [9–11] . Established initially to overcome health inequities in cancer care [12], system navigation has since expanded into areas such as chronic disease management [13, 14], mental health [15, 16], and to facilitate access to care for marginalized and historically underserved populations (e.g., persons experiencing homelessness, food insecurity, living in low-income countries) [17, 18]. Various terms are used in the literature to describe individuals who provide navigation support, such as patient navigators, community health workers, case managers, and link workers [17, 19]. For this review, system navigation is defined as programs that link the patient’s primary healthcare delivery and community-based health and social services to create integrated, patient-focused care [17, 20]. System navigation can be facilitated by an individual or team of lay and/or healthcare professionals to reduce barriers and facilitate access to continuous, effective, and efficient care for patients, caregivers, and providers [21].

Despite growing interest and calls to expand navigation programs for the general public to enhance integrated care delivery [1, 22], an understanding of the effectiveness of system navigation overall, and characteristics of effective models is largely unknown. A previous scoping review to identify navigation models [17] and factors influencing the implementation of navigation programs linking primary care with community-based health and social services [21] found the key motivators for implementing such programs included improving the delivery of health and social services to meet patient/population health needs and improve quality of life; however, this review included primarily descriptive, observational, and qualitative studies. In conclusion, Valaitis and colleagues [21] recommended a systematic review of primary care-based system navigation programs as a critical next step to determine program effectiveness and inform practice and policy decision-making related to optimal models and impacts.

As the body of literature has grown, this systematic review builds upon the previous scoping review of system navigation programs [17, 21] to identify the effectiveness of system navigation programs linking primary care with community-based health and social services to improve patient, caregiver, and health system outcomes when compared to usual care.

## Methods

This systematic review was registered with PROSPERO (CRD42020205050). The reporting of this review is based on PRISMA guidelines [23].

### Search strategy

The search strategy was built upon the previous scoping review of navigation programs linking primary care with community-based health and social services [17, 21]. Updating the previously conducted search, the electronic databases PsychInfo, EMBASE, CINAHL, OVID MEDLINE, and Cochrane Clinical Trials Registry were searched from January 1, 2013, to August 10, 2020 (Additional file 1). A health science librarian trained in building searches for systematic reviews consulted on the search strategy. In line with the previous scoping review, database searches were limited to studies published in the English language only.

### Study selection

Identified references were uploaded to Covidence (Veritas Health Innovation Ltd., Melbourne, Australia) and duplicates were removed. Titles and abstracts were independently screened by two reviewers for inclusion based on predetermined eligibility criteria. Full texts of potentially relevant studies were retrieved and screened by two independent reviewers. Conflicts were resolved through discussion or with the input of a third reviewer, as needed. Included studies from the previous scoping review [17, 21] were also reviewed independently and in duplicate to determine eligibility, as the previous review included qualitative and observational studies, in addition to intervention studies.

### Eligibility criteria

#### Types of studies

To determine intervention effectiveness, eligible studies were limited to experimental and quasi-experimental designs, including randomized controlled trials (RCTs), non-randomized controlled trials, and single group, pre-test/post-test intervention studies. Mixed methods studies with eligible quantitative designs were also included; however, only quantitative data were extracted. Qualitative, observational, descriptive, and cross-sectional studies were excluded.

#### Participants

Eligible studies included adults 18 years of age and older utilizing primary care. In contrast to the previous scoping review, studies that focused on disease-specific populations (e.g., cancer, mental health) were excluded to allow broader transferability and inform effective interventions to support health and social care access among general patient populations. However, studies that included patients with a variety of chronic diseases or chronic disease risk factors were eligible, given that the interventions described were not disease specific.

#### Interventions

System navigation programs based in a primary care setting that aimed to link patients to appropriate community-based health and social services were included. Primary care was defined as care delivered at the entry point into the healthcare system, which is typically provided by a physician or nurse practitioner [9]. Social prescription programs, which link users to community social services that may be considered outside of the healthcare system [9, 24], were eligible. In line with the original scoping review, we initially intended to include system navigation programs linking primary care to other medical specialty care services. However, we later decided to include interventions that went beyond health system navigation alone to focus on integrated, upstream, and community-based approaches. This decision was made in light of mounting evidence that integrated health and social care interventions focused on addressing the social determinants of health can improve health outcomes and reduce the use of costlier health services [25, 26]. Given the distinct role and function of case managers as clinical care providers, which may extend beyond the scope of system navigation [27], interventions that focused exclusively on case management were excluded. However, interventions that included a case management component in addition to system navigation were eligible.

#### Comparators

Studies that compared an intervention to any non-intervention comparison group were eligible, including pre-intervention data or data from a non-exposed control group.

#### Outcomes

The primary outcomes of interest were access to care (i.e., timely use of healthcare and/or social services to achieve improved health outcomes) and health and social service utilization. Secondary outcomes included patient-related (e.g., general health and wellbeing, quality of life, self-efficacy) and caregiver outcomes (e.g., caregiver burden, self-efficacy). Upon review of included studies, it became apparent that experience measures (e.g., satisfaction with the quality of care) and cost-related outcomes were also relevant. Thus, these other outcomes were added after the initial PROSPERO registration.

### Assessment of methodological quality

Two independent reviewers critically appraised all eligible studies to assess methodological quality using the Joanna Briggs Institute Critical Appraisal tools for experimental and quasi-experimental studies [28]. Conflicts were resolved through discussion between reviewers and input from a third reviewer when needed.

### Data extraction

Two independent reviewers extracted data using a pre-tested template; discrepancies were resolved through discussion or input from a third reviewer when needed. The data abstraction template included study characteristics (i.e., aim, study design, country), participant characteristics (i.e., number of participants, population description, age, sex, ethnicity, socioeconomic status), description of any comparator groups, limitations, and conclusions as reported by study authors. The Template for Intervention Description and Replication (TIDieR) checklist guided extraction of intervention components [29]. For relevant outcomes, the measure, effect, variation, and statistical significance were extracted. Authors were contacted to obtain missing data. Data collection forms are available upon request.

### Data synthesis

System navigation programs were grouped based on the navigation models identified in the previous scoping review, including lay person-led (i.e., non-healthcare professionals within primary care who perform specific activities related to system navigation), health professional-led (e.g., nurse or social worker who performs specific activities related to system navigation), and team-based (i.e., lay persons and health professionals together, or teams of health professionals) [17]. Results of individual studies were organized into tables by intervention type and outcomes (i.e., type, data collection tool, and measure of effect and significance) to facilitate synthesis and identify possible sources of heterogeneity. A meta-analysis was deemed inappropriate given the wide range of system navigation models and outcomes identified; instead, a narrative approach to synthesis was used [30], with data presented in corresponding tables. Reporting bias was not explored because most studies did not cite trial registrations or protocols. A comprehensive approach to assess the overall certainty of the evidence for each outcome (e.g., GRADE) was not used due to heterogeneity across interventions and outcomes.

### Patient and public involvement

Key research partners, including four older adult citizens and one community-based social service provider, were included in the review team. The aim of patient and public involvement in this systematic review was to support the interpretation of the results and identify key takeaways to inform the co-design of a community-based intervention to enhance physical activity, nutrition, and system navigation among older adults experiencing health inequities [31]. This was achieved through virtual working group meetings and the collaborative development of knowledge translation products, including a public-facing infographic and research brief.

## Results

### Description of included studies

**Fig. 1:**
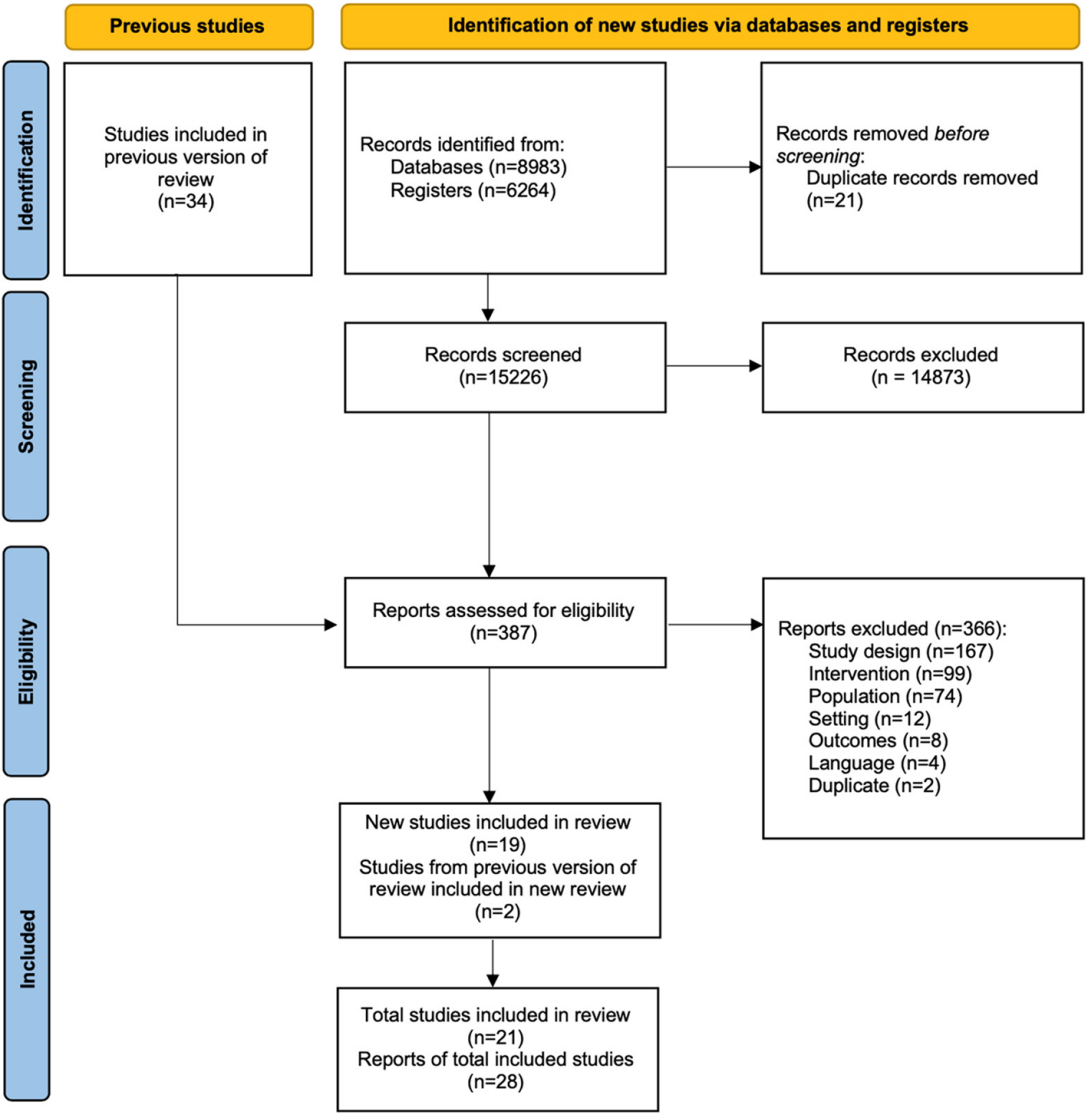
PRISMA Flow diagram

The updated search identified 15,226 unique records (Fig. 1). Following title and abstract screening, 387 full texts were retrieved and assessed for eligibility. A total of 21 studies published between 2009 and 2020 were included (Table 1); 19 of these were newly identified, and 2 were included in the previous scoping review. A list of excluded studies with reasons for exclusion is provided in Additional file 2. Study designs included RCTs (*n* = 8, 38%) [32–39], single group, pre-test/post-test designs (*n* = 7, 33%) [40–46], and two group, non-randomized designs (*n* = 6, 29%) [47–52]. Studies most often took place in the United States of America (*n* = 9, 43%) [32, 34, 35, 40, 43, 48, 50–52] or the United Kingdom (*n* = 8, 38%) [36, 41, 42, 44–47, 49]. A total of 10,743 participants (range 19 to 2,325 across studies) are represented, and, when mean ages were reported, the median mean age across studies was 72 years (range 49 to 82 years).

**Table 1:** Characteristics of included studies (*n* = 21)

Study	Study design	Country	Description of Intervention & Comparator Group	Population description	Ethnicity (%)	Socioeconomic status (%)	N started (completed) study	Mean ± SD age of participants (years)	Sex (%F)
Boult 2013 [32, 53, 54]	RCT	USA	I: Nurse-led Guided Care intervention including assessment of patient needs, care-planning and coordination, transitional care, monitoring, self-management, caregiver support and access to community-based services C: UC	≥ 65yrs, covered by fee-for-service Medicare, potentially eligible if HCC risk ratios were in the highest quartile of older patient population covered by own insurer	Caucasian: I, 51.1; C, 48.9 African American: I, 45.6; C, 46.3 Other: I, 3.3; C, 4.8	Monthly finances: Some money left: I, 57.9; C, 51.1 Just enough money: I, 32.8; C, 34.2 Not enough money: I, 9.3; C, 14.5	904 (477)	I, 77.2; C, 78.1 (SD NR)	I, 54.2; C, 55.4
Burger 2019 [40]	Mixed methods: Single group, pre-test/post-test	USA	I: Health-coach led self-management including care team communication, scheduling reminders, medication refills, referral to social services, emotional support and review of care plans C: Baseline	Adults with HTN or DM, at risk for ineffective health maintenance; physical and psychological capacity to meet self-management goals, speak English or Spanish, access to telephone	NR	NR	19 (16)	Range: 44–59	~ 60
Carnes 2017 [47]	Mixed methods: Two groups, non-randomized	UK	I: Social prescribing service coordinated by social workers with volunteer support, including action planning and referral to community services C: Matched patients from neighbouring area	GP patients with frequent visits and/or are socially isolated	NR	NR	486 (196)	Median: I, 56; C, 58	I, 58.9; C, 54.5
Dolovich 2016 [33, 55]	RCT	Canada	I: Health TAPESTRY, volunteer-led home visit to assess health status and goals, action planning with healthcare team including links to community support C: Wait-list control (UC)	≥ 70yrs, family health team patients not residing in LTC or receiving palliative/end-of-life care, English-speaking	European or white: I, 88.8; C, 86.5	High school: I, 38.0; C, 45.5 ≥ Post-secondary: I, 58.9; C, 48.7	312 (278)	I, 78.1 ± 6.3; C, 79.1 ± 6.6	I, 63.9; C, 60.4
Dye 2018 [48]	Two groups, non-randomized	USA	I: Volunteer health coach intervention, including needs assessment, home visits, self-management, education on use of self-monitoring equipment, linking to external services based on client needs C: Matched patients who chose not to participate	≥ 60yrs, residing in rural census, has a diagnosis of CVD, CHF, HTN, or DM	NR	NR	89 (69)	Range: I, 61–96; C, 62–91	61.5
Franse 2018 [49]	Two groups, non-randomized	UK, Greece, Croatia, Netherlands, and Spain	I: Care coordinator-led (variable by setting including social worker, nurse, nurse practitioner, physician assistant) Urban Health Centres Europe approach, including health assessment, shared decision making in development of care plan and referral to appropriate care pathways including health and social services C: UC	≥ 75yrs, living independently, comprehends local language, and make an informed decision on participation in the study, according to physician	NR	NR	2325 (1844)	I, 79.3 ± 5.7; C, 79.7 ± 5.5	60.8
Kangovi 2016 [35, 56]	RCT	USA	I: Goal setting plus IMPaCT, standardized intervention led by community health workers. Includes tailored coaching, social support, navigation, and advocacy C: Goal setting plus UC	1 visit at a study clinic in the prior yr and an upcoming appointment; lived in a high-poverty 5-ZIP code region in Philadelphia; diagnosed with ≥ 2 of the following CDs (HTN, diabetes, obesity, asthma/COPD with tobacco dependence) Excluded: worked with CHW before	African American: 94.7 Hispanic: 2.7	Household income < 15 000: I, 42; C, 47.4 Household income ≥ 15 000: I, 38; C, 33.6 Unknown: I, 20; C, 19.1	302 (NR)	Total, 56.3 ± 13.1; I, 56.6 ± 13.6; C, 56.1 ± 12.6	I, 76.7; C, 74.3
Kangovi 2018 [34]	RCT	USA	I: Goal setting plus IMPaCT, standardized intervention led by community health workers. Includes tailored coaching, social support, navigation, and advocacy C: Goal setting plus UC	≥ 18 yrs with appointment in the previous yr; living in identified high-poverty zip codes; uninsured/publicly insured; diagnosis for ≥ 2 CDs (≥ 1 in poor control), able to provide consent	African American: 94.3	Household income < 15 000: I, 65; C, 65 Household income ≥ 15 000: I, 23; C, 24 Unknown: I, 13; C, 12	592 (470)	52.6 ± 11.1	62.5
Kellezi 2019 [41]	Mixed methods: Single group, pre-test/post-test	UK	I: Health coach and link worker-led intervention that involved a needs assessment and then subsequent referral to relevant third sector groups C: Baseline	≥ 18 yrs, live or registered with GP in Nottingham), managing ≥ 1 long-term health conditions and feel isolated, lonely or anxious	NR	NR	630 (178)	52.7 ± 14.8	54.0
Loftus 2017 [42]	Single group, pre-test/post-test	Northern Ireland	I: Social worker-led social prescribing activities focused on health and well-being, emotional and practical support, education and self-help C: Patients who declined to participate	> 65 yrs with a chronic condition (i.e., falls, social isolation, depression/anxiety); poly-pharmacy (≥ 5 repeat medications) or a frequent GP attender	NR	NR	68 (28)	72.9 ± 7.3	70.6
Loskutova 2016 [43]	Mixed methods: Single group, pre-test/post-test	USA	I: Cities for Live Program, patient navigators assessed needs, barriers, limitations, stage of change and linked to 2–3 community programs C: Baseline	English-speaking, residing in Birmingham and receiving services at enrolled practices; type 2 diabetes diagnosis/risk or had prediabetes	Non-Hispanic: 76.5 Hispanic: 2.8 NR: 20.7	Some high school: 11.2; High school graduate: 20.1; Some college/ technical school: 11.7; College graduate: 14.5; Postgraduate/professional: 1.7; Unknown: 40.8	179 (179)	53.1 ± 12.2	73.2
Mayhew 2009 [44]	Single group, pre-test/post-test	UK	I: Integrated Care Coordination Service led by a care coordinator, includes identification of needs and liking to relevant health, social security or other organizations C: Baseline	> 65yrs, at risk of avoidable hospital admission, premature admission to institutional care, or concern due to medical, physical, emotional, or social issue	NR	NR	340 (93)	≅ 70% of participants > 75yrs; 50% > 85yrs	NR
Mercer 2019 [36]	RCT	Scotland	I: Community Links Practitioner intervention including assessing patient needs, linking to community organizations and if necessary, providing support to ensure attendance C: UC	≥ 18yrs, registered with intervention or comparator practice. Excluded if PC physician perceived participation is contraindicated	NR	Deprived: I, 79.3; C, 58.1 Employed: I, 24.1; C, 48.7	900 (775)	I, 49; C, 56	I, 59.2; C, 61.1
Pescheny 2019 [45, 57]	Single group, pre-test/post-test	UK	I: Social prescribing service led by trained non-clinicians that linked patients in primary care with sources of support within the community sector to improve their health, well-being, and care experience C: Baseline	PC patients with non-medical needs/psychosocial symptoms. Target groups included people with high risk/diagnosis of type 2 diabetes and COPD, mild to moderate mental health issues, experiencing loneliness and/or social isolation	NR	Not working: 61.8	186 (56)	51.2 ± 15.7	70.4
Spoorenberg 2018 [37]	RCT	Netherlands	I: Embrace, population-based integrated elderly care model (physician, nurse, social worker) including self-management support, introduction to community resources, and case management for those with complex care needs C: UC	≥ 75yrs, registered at participating GP, living at home/home for the elderly (not LTC)	NR	Low education: I, 49.9; C, 53.4 Low income: I, 44.1; C, 42.4	1456 (1131)	I, 80.6 ± 4.5; C, 80.8 ± 4.7	I, 54.2; C, 55.6
Taube 2018 [38]	RCT	Sweden	I: Registered nurse and physical therapist-led case management including monthly home visit, care plan development, healthcare system navigation, health information, information about local activities C: UC	≥ 65yrs, needing assistance in ≥ 2 self-reported ADLs, ≥ 2 hospital admissions or ≥ 4 outpatient care visits in last 12mos, no severe cognitive impairment	NR	Primary school: 46.4 Secondary school: 11.8 Vocational school: 35.9 Higher education: 5.9	153 (27)	81.5	66.7
Tung 2020 [50, 58]	Two groups, non-randomized	USA	I: “HealtheRx” intervention, electronic-medical record generated personalized list of local community resources with access to community health information specialist as needed C: UC	45-74yrs, insured by Medicaid and/or Medicare, sought care in the PC Clinic or ED, and resided in study area. Excluded: non-English speaking, lacked cognitive or physical capacity, recalled receiving a HealtheRx prior	African American: I, 89.5; C, 90.6	Annual household income: < $25,000: I, 48.3; C, 56.5 $25,00–49,999: I, 29.7; C, 21.1	420 (411)	45-54 yr: I, 25.8%; C, 30.7% 55-64 yr: I, 36.4%; C, 32.2% 65-74 yr: I, 37.8%; C, 37.1%	I, 72.7; C, 63.9
Vanderboom 2014 [51, 59]	Two groups, non-randomized	USA	I: Nurse-led Community Connections Program, including strengths assessment, action planning, crisis prevention plan, and circle of support, comprised of community and informal resources for self-management C: UC	≥ 55yrs, multiple chronic conditions, English speaking, and receiving PC from a health care home Excluded: cognitive impairment, untreated psychiatric condition, or terminal illness. Individuals identified by patient as someone supportive in their lives included as support persons	All Caucasian, non-Hispanics	~ 2/3 attended college	Patients: 62 (56) Support persons: 31 (NR)	NR	F > M
Wang 2015 [52]	Two groups, non-randomized	USA	I: Community health worker-led patient navigation including education, appointment scheduling, assistance with overcoming barriers to health care access C: Participants not reached by patient navigators	Type 2 diabetes and/or HTN diagnosis, unengaged with their medical care (not seen by PC physician in last 6mos)	Hispanic/Latino: 57.7 Race White 66% Black 30.2% Other 3.7%	NR	215 (206)	63.4 ± 12	54.9
Woodall 2018 [46]	Mixed methods: Single group, pre-test/post-test	UK	I: Social prescribing via wellbeing coordinators, including needs assessment and referral to local community health and wellbeing resources C: Baseline	≥ 14yrs, registered with a GP clinic	White: 90.6 Black (Caribbean, African, other): 3 Other 6.5	NR	434 (342)	53.1 ± 18	63.9
Zhang 2018 [39]	RCT	China	I: Older person-centred and integrated health management model programme intervention led by community health centre staff and multidisciplinary care team including self-management, health management, referral to community programs, and family participation C: Bimonthly health education	≥ 60yrs, lived in the community for ≥ 2 yrs. Excluded: cognitive deficits, severe chronic illnesses, multiple life-threatening comorbidities, and life expectancy < 1 yr, current or previous participation in another trial within the past 30d	NR	≤ Primary school: I, 57.9; C, 58.1 iddle school: I, 28.2; C, 26.6 ≥ College: I, 13.9; C, 15.3	671 (637)	70.5 (SD NR)	51.9

*ADL* activities of daily living, *C* control group, *CD* chronic disease, *CHF* congestive heart failure, *CHW* community health worker, *COPD* chronic obstructive pulmonary disease, *CVD* cardiovascular disease, *d* day, *DM* diabetes mellitus, *F* = female, *GP* general practitioner, *HCC* hierarchical condition category, *hr* hour, *HTN* hypertension, *I* intervention group, *IMPaCT* Individualized Management for Patient-Centered Targets, *LTC* long-term care, *M* male, *min* minute, *mo* month, *NR* not reported, *PC* primary care, *RCT* randomized controlled trial, *SD* standard deviation, *UC* usual care, *wk* week

Primary care-based system navigation program models included 1) lay person-led (*n* = 10, 48%) [34–36, 40, 43–46, 48, 52], 2) health professional-led (*n* = 4, 19%) [32, 42, 49, 51], and 3) team-based (*n* = 6, 29%) [33, 37–39, 41, 47]. A fourth model was also identified, which included self-navigation based on a personalized list of local resources with lay support available (*n* = 1, 5%) [50]. In studies that used a primarily lay person-led model, most (*n* = 7, 70%) described comprehensive navigator training and employed lay navigators as staff [34, 35, 40, 43, 45, 48, 52]. This training ranged from 3 h of online training [43] to a 16-week community college health coaching course [40]. In studies that used health professional-led models, system navigation was primarily nurse-led [32, 49, 51] or social worker-led [42, 49]; however, in one multi-site study, health professionals varied by setting and also included a nurse practitioner or physician assistant in system navigation roles [49]. The team-based navigation models included either lay person(s) and health professional(s) together [33, 39, 41, 47] or teams of health professionals [37, 38] who provided system navigation support.

Intervention duration and frequency of contact were highly variable across the included studies. The median length of system navigation programs was 6 months (range 2 to 30 months). Of the 17 studies that reported intervention frequency, most programs were delivered variably based on individual patient needs (*n* = 9, 53%) [33, 36, 41–43, 45–47, 49], while others occurred monthly (*n* = 4, 24%) [32, 34, 38, 40], weekly (*n* = 2, 12%) [35, 48], bi-monthly (*n* = 1, 6%) [39], or one-time-only (*n* = 1, 6%) [50]. Theoretical models or frameworks were reported in only 33% (*n* = 7) of studies to support the rationale for system navigation programs; these included the Chronic Care Model [33, 37, 48, 51], the biopsychosocial model [45], the integral conceptual model of frailty [49], and a theory of community-based primary care [36]. A full description of intervention characteristics based on the TIDieR framework is presented in Table 2.

**Table 2:** Intervention characteristics

Study	What	Why	Who provided	How	Where	When and how much	Tailoring	Modifications	How well (Adherence/fidelity)
Boult 2013 [32, 53, 54]	Eight Guided Care services: - Home-assessment of patients’ needs and goals - Care planning - Proactive monitoring - Care coordination - Transitional care - SM coaching - Caregiver support - Access to community-based services	To combine effective chronic care interventions into a single delivery model for wide adoption within PC	RNs + 2–5 PC physicians trained in Guided Care model	1:1, telephone (participants), group (caregivers and participants)	PC, home	Duration: 32mos Frequency: Individual care at least monthly; caregiver SM course 10 h over 6wks, monthly support group, ad-hoc calls	Care plan personalized to patient and caregiver preferences, priorities, and intentions	NR	NR
Burger 2019 [40]	Health coach facilitated communication with care team and promoted patient engagement to pursue provider-created care plans. Care plan, barriers and facilitators to goals were reviewed with patient, communicated to care team through daily huddles and electronic health record	PC physicians have limited time with patients. Including SM in patient care through health coaches may assist in more effective treatment for chronic conditions	Health coaches: Experienced medical assistants who completed a 16wk training course from local community college	1:1, telephone	Community health centre, PC clinic	Duration: 6mos Frequency: Variable (Approx. 5–7 total sessions)	NR	NR	NR
Carnes 2017 [47]	Meetings with SP coordinator and volunteer to develop and execute action plans, including goal setting and referrals to community organizations and services (e.g., exercise, cooking classes)	Commissioned pilot project to improve patient well-being and increase personal self-efficacy to reduce PC resource use	SP coordinators: Trained in social work, employed by a managing third sector organization Volunteers: Trained by coordinator to assist in service delivery and provide patient support	1:1, in-person	PC centres	Duration: 8mos Frequency: Up to 6 sessions with SP coordinators; unlimited volunteer contacts	Goals developed in collaboration with patient and SP coordinator	NR	13.9% no contact with services; 69.2% single consultation only
Dolovich 2016 [33, 55]	Health TAPESTRY intervention: - Home visit with trained volunteer pair to collect information on intervention-designed “TAP-app” on goals, daily life activities and general health - Electronic report sent to clinical team - Clinic team reviews reports and connects with interprofessional healthcare team and PC physician - Care plan is collectively generated	Intervention developed to combine core elements of the Chronic Care Model (healthcare organization and leadership, linkage to community resources, client SM, coordination of delivery, clinical decision support and clinical information systems) into a coordinated approach to improve PC delivery and promote optimal aging	Lay-volunteers: Trained in 2 h in-person training session and ongoing online sessions Clinical intake team comprised of various healthcare team members and PC physician at PC clinic	In-person	PC, home	Duration: 6mos Frequency: Initial home visit with f/u ‘as needed’	Care plan tailored to individuals’ goals and needs	NR	NR
Dye 2018 [48]	Health coach provided education on SM skills, coordination of health care services and referrals, links to community resources, medication management, appointment scheduling and treatment reminders, transportation arrangements, and facilitated communication between client, caregiver, service providers and PC. Digital blood pressure, scales or glucose monitor based on client needs, and patient Personal Health Diary for symptom tracking. Diary reviewed on subsequent visits	Evidence suggests gaps in transition to home health care following hospitalization. Following the Chronic Care Model can help patients meet SM needs	RNs linked patients with health coach Health coaches: Community members received 30 h training and must score ≥ 80% on knowledge test	1:1, in-person, telephone	Home	Duration: 4mos Frequency: Approx/ 3.5 h/wk in mo 1; 3 h/wk in mo 2; 2.5 h/wk in mo 3; 2 h/wk in mo 4	Tailored to the needs of the client and/or caregivers	NR	NR
Franse 2018 [49]	Multidimensional health assessment of fall risk, polypharmacy, loneliness and frailty. Shared decision making to develop care plan and referral to care pathways to promote heathy aging, such as falls prevention (exercise, multifactorial programs), polypharmacy (self-monitoring, pharmaceutical care), loneliness (social activities/support), and frailty (medical management, exercise)	Integral, multidisciplinary conceptual model of frailty: physical, social and psychological components Intervention co-designed based on current evidence and stakeholder input via intervention mapping	Care coordinator: Trained assistant supervised by PC physician, social worker, community nurse or geriatric nurse practitioner (depending on site)	1:1, in-person	Home or senior health centre	Duration: 12mos Frequency: Variable	Tailored to preferences of older adults, results of the short-standardized assessment form, and pathways available	Age was lowered to ≥ 70 in 2 cities Designed to use existing services, when limited/difficult to access new services developed	NR
Kangovi 2016 [35, 56]	IMPaCT intervention consisted of goal setting with PC provider and connecting with a CHW for tailored coaching, social support, advocacy and navigation through 3 phases of action planning, tailored support and connection with long-term support	Intervention had previously been tested in hospitalized patients with positive effects and was then adapted to support outpatients with multiple chronic conditions	CHW from community organizations, underwent mo-long, college-accredited course and mentorship from a senior CHW	1:1, in-person, telephone, text	Home, community	Duration: 6mos Frequency: At least 1x/wk (mean 38.4 h total)	Activities and resources tailored to patient goal	NR	82% participants engaged in full 6mos Mean 4.6 action plans/participant created
Kangovi 2018 [34]	CHWs developed action plan for goals set with PC physician, provided tailored coaching, social support, advocacy, and navigation to appropriate clinician for health education or clinical care. Long-term supports (e.g., neighbours, family, church, support groups) identified for post-intervention SM. Link Worker connects patient to relevant third-sector groups for f/u	As many clinicians are unable to address social issues, evidence suggests lay CHWs can perform various roles to support and improve chronic disease management	Lay CHWs with at least a high school diploma, undergo behavioural interviews and mo-long training. Supervised by a manager, typically master’s degree in social work, for ongoing support, training and clinical integration	1:1, in-person	PC	Duration: 6mos Frequency: Monthly	Tailored to each patient care plan, and relevant to each site using a structured approach	NR	91% completed intervention Mean 5.5 (SD 2.0) action plans per person 60.3% action plans completed
Kellezi 2019 [41]	SP pathway: - Initial needs assessment with health coach - SM or referral to link worker for connection with relevant third-sector groups - Health coach and link worker regularly check patients’ progress	SP pathway implemented within GP practices to increase SM, improve health and reduce PC usage amongst individuals with chronic illness experiencing loneliness	Initial program referral from PC physician Health coach: Unspecified health professional Link worker: Unspecified community-based worker	1:1, in-person	NR	Duration: 8wks, Frequency: Initial meeting plus variable f/u based on patient needs	Tailored to patients’ needs	NR	NR
Loftus 2017 [42]	SP pathway: Home visit conducted to select programs (e.g., social clubs, Men's Shed, counselling, arts, falls prevention, exercises, crocheting, personal development, crafts, befriending, computer courses)	In the UK, all PC physicians are encouraged to consider SP, but many do not. This has the potential to decrease PC workload, but this has not been confirmed	PC physician referred to program SP coordinator: Qualified social worker in community health care	1:1, in-person	Home, community	Duration: 12wks Frequency: 1 home visit; frequency of programs variable	NR	NR	Mean 92 days from referral to starting SP activity 59% of patients did not join any programs
Loskutova 2016 [43]	PNs assessed patients’ needs, barriers, limitations, and stage of readiness to change with diabetes management, and offered support and encouragement to link to 2–3 appropriate community programs. Follow-up letters and reminders were used to encourage participation and monthly feedback reports were provided to PC and community programs	Evidence indicates that PN can improve health outcomes. Many of the services needed for diabetes care can be provided by community organizations and navigation could be provided by non-health workers via telephone	Referral from PC physician 2 × 0.5 FTE lay PNs: non-health professional community members familiar with local resources, backgrounds in community programming or research, underwent 2 × 1.5 h online training sessions	1:1, telephone, email, mail	Home, community	Duration: Variable (mean 120.4 ± 50.5 days, range 1–260) Frequency: Variable (mean 6.1 calls/patient, range 2–15)	NR	69.1% of calls successful 7.8% of patients never reached	Project manager participated in ongoing review and feedback sessions
Mayhew 2009 [44]	Integrated Care Coordination Service provides initial home assessment, ongoing follow-up, and coordination health and social care (e.g., home assistance, living arrangements, financial advice, referrals to health and social care provider in public, private, volunteer sector) based on identified needs	Many hospital admissions could be prevented by early treatment of social factors. This initiative aims to reduce costs through prevention	PC physicians, family/friend, or self-referral Lay care coordinator (not described)	1:1, in-person	Home	Duration: 3mos Frequency: Initial in-home visit, unspecified number of f/u contacts	Tailored to patients’ needs	NR	NR
Mercer 2019 [36]	Link Worker Program - Community links practitioner identifies patients’ needs - Links to local community organizations (e.g., walking groups, finance, welfare, addiction support, socialization) - Support to encourage attendance, if needed - PC staff supported to set up referral systems	Drawing on a theory of community-based PC, patients in deprived areas often have multiple issues not amenable to medical intervention. Community organizations offer many resources but are inaccessible to many. Closer links between PC and community organizations may support better access	PC physicians and nurses refer Community links practitioner: Experienced in community development and working with community organizations	1:1, in-person, some telephone	PC, home, community	Duration/Frequency: Variable; as many times and when necessary	Flexible and dependent on patient needs, wants and professional judgement	NR	NR
Pescheny 2019 [45, 57]	SP pathway: - Assessment of patients’ non-medical needs - Motivational interviewing - Personalized support - Link to non-medical support and referrals to third sector programs (e.g., finance, housing, employment, physical activity, gardening, social activities, stress management, creative activities) - Re-assessment and exit interview	A biopsychosocial model is needed because of wider determinants of health, integration of care across professionals, and changing needs of populations	PC physicians refer to program PNs: Non-clinicians employed in primary care practices, received targeted training to perform navigation and refer patients to third sector organizations	NR	PC	Duration: NR Frequency: Variable (based on individual needs), approx. 12	Referred to services based on patients’ needs	NR	70% lost to f/u or did not engage with SP service after initial assessment
Spoorenberg 2018 [37]	Embrace person-centred integrated care service, SM support and prevention including: - Community meetings - Links to local healthcare and welfare organizations (health maintenance, physical and social activity, diet) - Individual support from a case-manager to develop care plan targeting health-related problems	Following the Chronic Care Model and a Population Health Management model (Kaiser Permanente Triangle) to support older adults to age in place through person-centered, integrated, proactive, and preventive support and care	PC physicians refer Elder Care team includes PC physician, nursing home physician and two case managers (nurse and social worker), all take part in intensive training program	1:1 and group, in-person	Home and community	Duration: 12mos Frequency: NR	Tailored to participants’ risk profile of robust, frail or complex needs	NR	NR
Taube 2018 [38]	- Assessment of lifestyle, functional and cognitive status, monitoring and evaluation, care coordination and encouragement of social activities - General health system information and specific information to address participants’ needs and psychosocial aspects - Continuity and safety	There is evidence that comprehensive case management can benefit a client’s perception of psychological support in terms of providing reassurance, feelings of security and social support	2 case managers: RN focused on health, medications, and psychosocial aspects; physical therapist focused on fall prevention and physical functioning	1:1, in-person, telephone	Home	Duration: 12mos Frequency: At least monthly	Based on patients’ care needs, goals of care	Pilot phase only include RN case manager, PT added	NR
Tung 2020 [50]	CommunityRx intervention: All participants receive a “HealtheRx” including location, hours, and fees for 2 resources closest to patient’s home - Interventions focused on basic needs, physical and mental wellness, and disease management including smoking cessation, weight loss, and counseling based on an evidence-based algorithm - Contact information for community health information specialist also provided	Most referral interventions rely on costly staff to implement such as case managers or CHWs, which can be difficult to implement within routine clinic workflow. An IT solution may reduce cost and healthcare burden	Nurse in ED or administrative staff in PC refer Community health information specialist available (details not provided)	Electronic	PC, ED	Duration: NR Frequency: One time referral	Resource referrals individually tailored	NR	NR
Vanderboom 2014 [51, 59]	Community Connections Program: - Initial strengths assessment including identification of priority needs and development of an action plan, crisis prevention plan, and circle of support - Ongoing f/u provided to problem solve, strengthen supports and coordinate with community services - Nurse care coordinator, patient and support person using “Wraparound” to coordinate the use of comprehensive community-based services	Based on the Chronic Care Model developed in response to widespread inefficiencies of chronic illness care and the need for a multi-faceted, evidence-based model. The Chronic Care Model proposes that effective partnerships between health and community providers are a key element to support patient SM	Nurse care coordinator. Training included strategies for conducting strengths assessments, identifying holistic care needs, and developing care plans to address concerns	1:1, in-person	Home	Duration: 3mos Frequency: Initial meeting, unspecified ongoing f/u	Plan of care tailored to patients’ needs	NR	NR
Wang 2015 [52]	PNs delivered patient-centered education about f/u care, appointment scheduling, assessing needs for specialist referral, identifying challenges to accessing healthcare and aiding to overcome challenges	Evidence of effectiveness of in-person and telephone-based PN in improving access to cancer screening, diagnosis, and treatment in racial/ethnic minority populations. The role of the patient in chronic disease management is not well understood	3 lay PNs: community members trained by the healthcare team and completed CHW training program	1:1, primarily telephone, follow-up via letter or home outreach	Home	NR	NR	NR	Only 31% eligible reached by navigator, and 21% scheduled appointment
Woodall 2018 [46]	SP service: Well-being coordinators assess user needs, offer support, and provide advice on local groups and services (e.g., mental health and counselling, fitness classes, support for physical or emotional difficulties, finance advice and creative groups)	Despite suggestion that SP can reduce burden on PC services, evidence is lacking, and most current programs lack evaluative components or show mixed results	Wellbeing coordinator: Diverse ages, ethnicities, and professional experiences, understood working in marginalized communities	1:1, mostly telephone but in-person for complex cases	NR	Duration: Mean 10wks (most < 16wks) Frequency: Up to 6 sessions	Involvement of specific services/ programs tailored to participants’ needs	NR	NR
Zhang 2018 [39]	Older person-centred and integrated health management model, includes SM, individual health management, community health management (e.g., classes to encourage healthy behaviours), and family management	Few studies have investigated and evaluated effective interventions for multiple healthy lifestyle factors, but many have shown promising results	Community health service centre staff, multidisciplinary team	In-person, telephone, individual, group	Hospital or community centre	Duration: 24mos Frequency: Once every 2mos, all participants were visited at 12 and 24mos	Individual interventions based on health assessment and counselling	NR	NR

*CHW* community health worker, *d* day, *ED* emercengy department, *f/u* follow-up, *GP* general practitioner, *hr* hour, *min* minute, *mo/s* month(s), *PC* Primary care, *PN* patient navigator, *SM* self-management, *SP* social prescribing, *wk* week

### Methodological quality

**Fig. 2:**
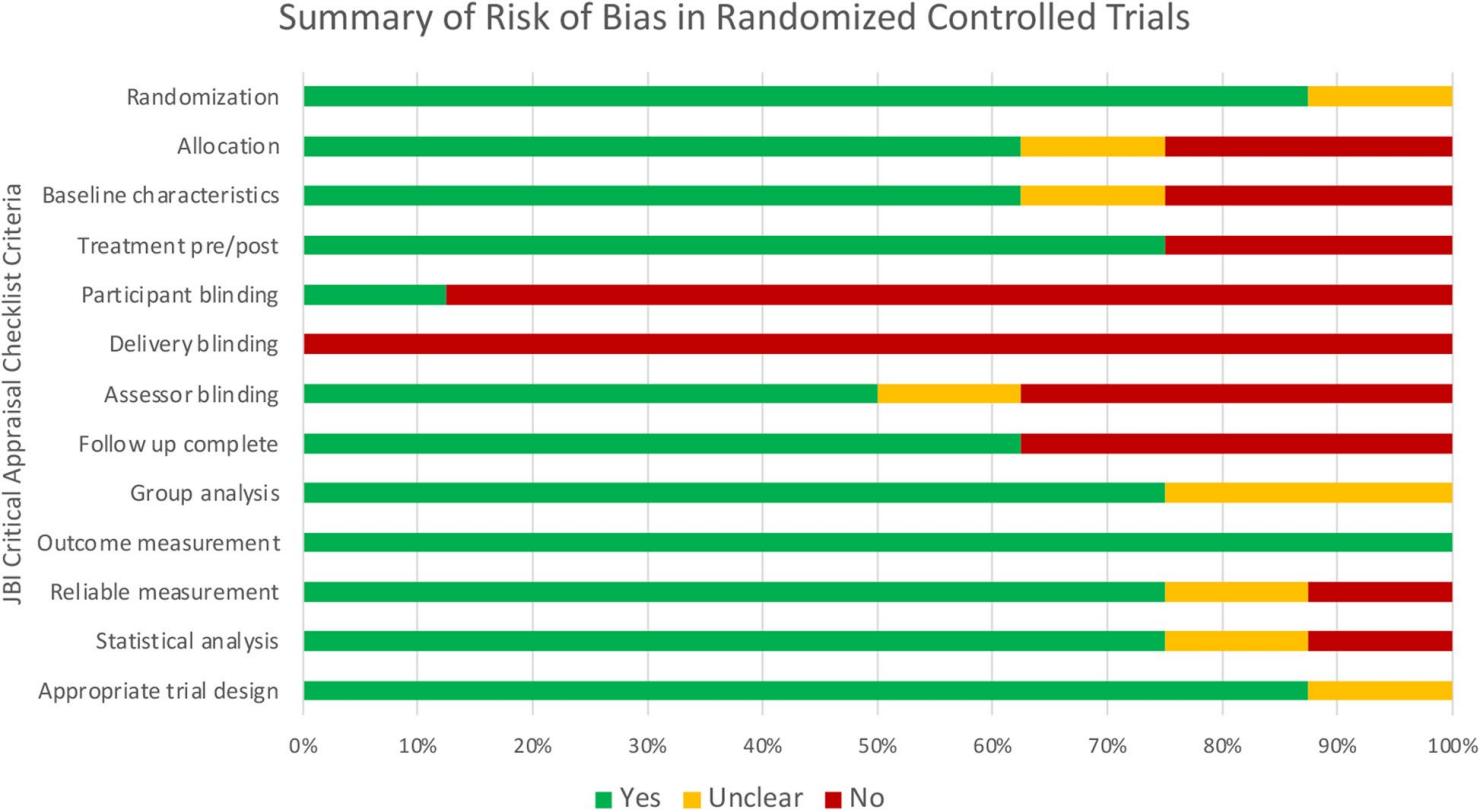
Assessed using JBI Critical Appraisal Checklist for Randomized Controlled Trials

**Fig. 3:**
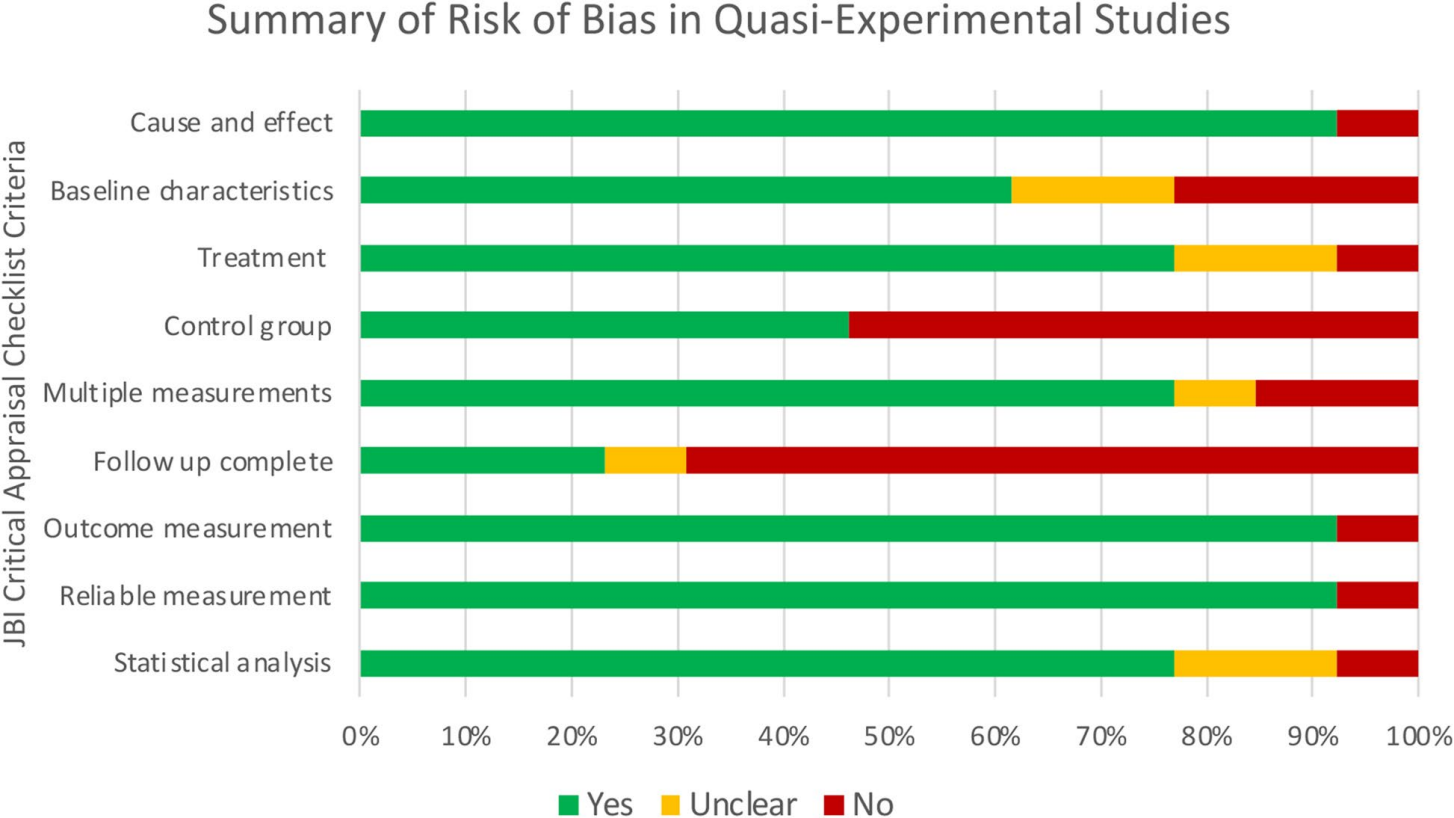
Assessed using JBI Critical Appraisal Checklist for Quasi-Experimental Studies (includes single-group, pre-test/post-test and two-group, non-randomized study designs)

Overall, the included studies had generally low to moderate risk of bias. Within the 8 RCTs, the risk of bias was primarily attributed to the absence of blinding among participants and interventionists (Fig. 2). The lack of control groups and incomplete follow-up predominantly contributed to the risk of bias among the 13 quasi-experimental studies (Fig. 3). Full critical appraisal assessments for each study are reported in Additional files 3 and 4 for RCTs and quasi-experimental studies, respectively.

### Effectiveness of system navigation programs

A summary of findings by system navigation model and outcome category alongside a summary of the risk of bias is provided in Table 3. Complete data used for analyses for each outcome are provided in Additional files 5–9.

**Table 3:** Summary of results

	**TYPE OF SYSTEM NAVIGATION MODEL**											
	**LAY PERSON-LED** **(*n* = 10)** Non-professional trained to perform specific activities related to system navigation			**HEALTH PROFESSIONAL-LED** **(*n* = 4)** Health professional (e.g., social worker, nurse) performs specific activities related to system navigation			**TEAM-BASED** **(*n* = 6)** Lay person(s) and health professional(s) together, OR teams of health professionals perform specific activities related to system navigation			**SELF-NAVIGATION WITH LAY SUPPORT** **(*n* = 1)** Personalized list of local resources with lay support available, as needed		
**OUTCOME CATEGORY**	**# of studies**	**Overall impacts**	**Risk of bias**	**# of studies**	**Overall impacts**	**Risk of bias**	**# of studies**	**Overall impacts**	**Risk of bias**	**# of studies**	**Overall impacts**	**Risk of bias**
**HEALTH AND SOCIAL SERVICE ACCESS AND UTILIZATION OUTCOMES**												
Health service utilization	6	↔	Low-moderate	4	↔	Low-moderate	3	**↑**	Low	0	-	-
**PATIENT-RELATED OUTCOMES**												
Quality of life/health-related quality of life, mental health, wellbeing	5	↔	Low-moderate	3	↔	Low-moderate	4	↔	Low-moderate	1	↔	Low
Social participation and function	2	↔	Moderate	2	↔	Low	4	↔	Low-moderate	0	-	-
Health behaviours	4	↔	Low-moderate	1	↔	Low	2	↔	Low–high	0	-	-
Patient activation, self-efficacy, empowerment	3	↔	Low-moderate	1	↔	Low	0	-	-	1	**↑**	Low
**PATIENT EXPERIENCE OUTCOMES**												
Patient experiences	2	**↑**	Low-moderate	2	**↑**	Low-moderate	1	↔	Low	0	-	-
**CAREGIVER OUTCOMES**												
Caregiver experiences	0	-	-	2	↔	Low-moderate	0	-	-	0	-	-
Caregiver health	0	-	-	1	↔	Moderate	0	-	-	0	-	-
**COST-RELATED OUTCOMES**												
Cost-related	2	Insufficient data	Low-moderate	0	-	-	0	-	-	0	-	-

↑ = all studies reported statistically significant improvements; ↔ = some of the studies reported statistically significant improvements while other studies reported non-statistically significant findings, or none of the studies reported statistically significant findings

#### Health and social service access and utilization outcomes

The 13 studies that reported health service utilization evaluated lay person-led (*n* = 6, 46%) [34, 35, 40, 44, 48, 52], health professional-led (*n* = 4, 31%) [32, 42, 49, 51], and team-based (*n* = 3, 23%) [33, 41, 47] system navigation models. Health service utilization was primarily captured through administrative, health record, and/or health insurance data related to the number of primary care visits (*n* = 10, 77%) [32, 33, 35, 40–42, 47, 49, 51, 52], hospital admissions and/or readmissions (*n* = 9, 69%) [32–35, 40, 44, 48, 49, 51], emergency care visits (*n* = 7, 54%) [32, 33, 40, 44, 47, 48, 51], and home care visits (*n* = 4, 31%) [32, 42, 48, 51] (Additional file 5). None of the included studies reported healthcare access or social service utilization outcomes.

Overall, findings for lay person-led models were mixed. Three studies demonstrated improvements in health service utilization following lay person-led system navigation programs [34, 44, 52]. Compared to baseline, patients at high risk for avoidable hospital admissions due to medical or psychosocial issues who accessed the lay person-led Integrated Care Coordination Service had a statistically significant decrease in emergency department attendance and hospital admissions nine months post-referral (low risk of bias) [44]. Patients living in high-poverty areas who participated in the standardized, 6-month community health worker-led goal setting plus Individualized Management for Patient-Centered Targets (IMPaCT) program (tailored coaching, social support, navigation, advocacy) also had significantly lower odds of repeat admissions, but no difference in overall hospital admissions or length of stay when compared to goal setting plus usual care (low risk of bias) [34]. Compared to usual care, community health worker-led system navigation including patient education, appointment scheduling, and assistance overcoming barriers to healthcare access significantly increased the rate of primary care provider and/or chronic disease nurse visits among patients with chronic health needs who were classified as unengaged with their medical care (i.e., had not seen a primary care physician in last 6 months) (moderate risk of bias) [52]. Further, a higher percentage of these patients visited a primary care provider before seeking other providers for their health needs [52]. However, three studies demonstrated no significant changes following lay person-led system navigation programs when compared to baseline or usual care (moderate risk of bias) [35, 40, 48].

Similarly, the effectiveness of health professional-led system navigation on health service utilization outcomes was unclear. A social worker-led social prescribing program for patients with chronic conditions, polypharmacy, or frequent primary care attendance was associated with a significant decrease in the number of primary care physician visits, but no difference in home visits, telephone visits, or care contacts when compared to usual care in one study (moderate risk of bias) [42]. No significant impacts on health service utilization were observed in three other studies following health professional-led system navigation programs when compared to usual care (low-moderate risk of bias) [32, 49, 51].

In contrast, team-based system navigation models demonstrated some positive impacts on health service utilization across three studies with low risk of bias [33, 41, 47]. In the 6-month Health TAPESTRY program, volunteer-led home visits followed by action planning with the healthcare team and links to community support resulted in a statistically significant increase in primary care visits and reduced rates of hospitalization among older adults, with no significant changes in emergency department visits when compared to usual care [33]. Similarly, social worker and volunteer-led social prescribing to community services resulted in a significantly lower rate of annual general practitioner consultations with no significant impact on emergency department visits among adult patients experiencing social isolation with a history of frequent primary care visits, as compared to matched patients from a neighbouring area [47]. However, it should be noted that this study lacked randomization, and patients assigned to the intervention group had a significantly higher rate of general practitioner consultations at baseline compared to their matched counterparts. Finally, a health coach and link worker-led intervention involving a needs assessment and referral to relevant community services also significantly decreased primary care use over a 3-month time period among patients managing at least one long-term health condition and experiencing social isolation when compared to baseline [41].

#### Patient-related outcomes

In total, 16 studies captured patient-related outcomes [32–39, 41, 43, 45–47, 49–51]. These were grouped into four categories: 1) quality of life/health-related quality of life, mental health, and wellbeing, 2) social participation and function, 3) health behaviours, and 4) theoretical constructs related to behaviour change.

##### **Quality of life/health-related quality of life, mental health, and wellbeing**

In total, 13 studies investigated the impact of lay person-led (*n* = 5, 39%) [34–36, 46, 57], health professional-led (*n* = 3, 23%) [32, 49, 51], team-based (*n* = 4, 31%) [33, 37, 38, 47], and self-navigation with lay support as needed (*n* = 1, 8%) [50] system navigation models on quality of life/health-related quality of life, mental health, and wellbeing outcomes. These outcomes were most often measured using the 12- or 36-Item Short Form Survey (SF-12, SF-36) (*n* = 5, 39%) [32, 34, 35, 49, 50], EuroQol-5 Dimension (*n* = 5, 39%) [33, 36, 37, 46, 51], Hospital Anxiety and Depression Scale (*n* = 2, 15%) [36, 47], or the Warwick-Edinburgh Mental Wellbeing Scale (*n* = 2, 15%) [45, 46]. Various other single-item and self-report measures were used (Additional file 6).

Findings for lay person-led system navigation models were mixed. Social prescribing to local community health and wellbeing resources resulted in reduced anxiety and depression, better self-reported health, as well as a statistically and clinically significant improvement in patient wellbeing when compared to baseline in one study (moderate risk of bias) [46]. However, another social prescribing program found a statistically significant, but not clinically significant difference in wellbeing among patients with multiple chronic conditions experiencing social isolation/loneliness when compared to baseline (moderate risk of bias) [57]. Further, no significant changes in wellbeing, anxiety, depression, or health-related quality of life were found following the Community Links Practitioner intervention when compared to usual care (high risk of bias) [36]. The standardized goal setting plus IMPaCT intervention significantly improved health-related quality of life in the mental domain, but not the physical domain of the SF-12 when compared to goal setting plus usual care in one study (moderate risk of bias) [35]. However, no significant changes were observed in physical or mental health-related quality of life in another study evaluating the goal setting plus IMPaCT intervention when compared to usual care (low risk of bias) [34].

Findings for health professional-led system navigation models were also mixed. The Urban Health Centres Europe approach including health assessment, shared decision making, and referral to appropriate health and social service care pathways (led by either a social worker, nurse, nurse practitioner, or physician assistant based on the setting) significantly improved health-related quality of life compared to usual care (low risk of bias) [49]. However, two studies using nurse-led system navigation models did not result in significant improvements in health-related quality of life compared to usual care (low-moderate risk of bias) [32, 51]. None of the team-based or self-navigation with lay support system navigation models significantly improved quality of life/health-related quality of life, mental health, or wellbeing outcomes compared to baseline or usual care (low-moderate risk of bias) [33, 37, 38, 47, 50].

##### **Social participation and function**

Social participation and function was evaluated in eight studies including lay person-led (*n* = 2, 25%) [36, 46], health professional-led (*n* = 2, 25%) [49, 51], and team-based (*n* = 4, 50%) [33, 38, 41, 47] system navigation models. Various measures were used, including heterogeneous assessments of loneliness [38, 41, 49], social networks [33, 46], participation in social roles [36, 47, 51], and social group memberships [41] (Additional file 6). Overall, the findings were mixed. Of the lay person-led models, social prescribing by wellbeing coordinators significantly increased social networks compared to baseline in one study (moderate risk of bias) [46]. However, no changes in social participation were found following the Community Links Practitioner intervention compared to usual care in another study (high risk of bias) [36]. Neither of the studies that used a health professional-led model found significant differences in social participation and function outcomes (low risk of bias) [49, 51]. Of the team-based models, the health coach and link worker-led intervention for adults managing long-term health conditions and experiencing social isolation, loneliness, or anxiety significantly improved the number of social group memberships from baseline, but did not impact community belonging or loneliness (low risk of bias) [41]. Three additional studies evaluating team-based system navigation models found no significant differences in social participation and function outcomes (low-moderate risk of bias) [33, 38, 47].

##### **Health behaviours**

Health behaviours were assessed in seven studies evaluating lay person-led (*n* = 4, 57%) [34–36, 45], health professional-led (*n* = 1, 14%) [49], and team-based (*n* = 2, 29%) [33, 39] system navigation models. Outcomes included heterogeneous measurements of physical activity/exercise [33, 36, 39, 45, 49], cigarette smoking [34, 35, 39], alcohol intake [39, 49], and diet [39] (Additional file 6). Overall, the findings were mixed. Lay person-led social prescribing significantly increased physical activity compared to baseline in one study (moderate risk of bias) [45]. However, three additional studies evaluating lay person-led models found no significant differences in health behaviour outcomes, including cigarette smoking or exercise level (low-moderate risk of bias) [34–36]. The study that evaluated a health professional-led model compared to usual care did not find significant differences in healthy lifestyle behaviours (low risk of bias) [49]. Of the team-based system navigation models, an integrated health management intervention with referral to community programs led by community health centre staff and a multidisciplinary care team led to significant improvements in health behaviours including physical activity, alcohol intake, diet, and smoking habits when compared to bimonthly health education (high risk of bias) [39]. However, another team-based model did not significantly impact physical activity levels compared to usual care (low risk of bias) [33].

##### **Patient activation, self-efficacy, and empowerment**

Patient activation, self-efficacy, and empowerment were evaluated in five studies including lay person-led (*n* = 3, 60%) [34, 35, 43], team-based (*n* = 1, 20%) [33], and self-navigation with lay support as needed (*n* = 1, 20%) [50] system navigation models. Heterogeneous measurements of self-efficacy [33, 43, 50], patient activation [34, 35], and empowerment [33] were used. Overall, the findings were mixed. Of the lay person-led models, the Cities for Live Program including linkage to community programs following an assessment of needs, barriers, and stage of change significantly improved self-efficacy compared to baseline (moderate risk of bias) [43]. However, the standardized lay person-led goal setting plus IMPaCT intervention did not change patient activation in two studies (low-moderate risk of bias) [34, 35]. No significant changes in goal attainment, self-efficacy, or patient empowerment were observed following team-based system navigation in one study (low risk of bias) [33]. Although limited to evidence from one study evaluating a self-navigation with lay support system navigation model, patients who participated in the “HealtheRx” intervention involving an electronic-medical record generated personalized list of local community resources with access to a community health information specialist as needed were more likely to report higher confidence in finding resources in their community to help manage their health compared to usual care (low risk of bias) [50].

#### Patient experience outcomes

Patient experience outcomes were reported in five studies, including lay person-led (*n* = 2, 40%) [34, 35], health professional-led (*n* = 2, 40%) [32, 51], and team-based (*n* = 1, 20%) [33] system navigation models. Patient experiences with care quality were measured using the Consumer Assessment of Healthcare Providers and Systems-Patient Centered Medical Home survey [34, 35], Patient Assessment of Chronic Illness Care tool [32, 51], and Canadian Institute for Health Information common indicators [33] (Additional file 7). Both lay person-led and health professional-led system navigation models consistently improved patient experiences with quality of care. The community health worker-led goal setting plus IMPaCT intervention significantly improved care comprehensiveness and self-management supportiveness when compared to goal setting plus usual care in two RCTs (low-moderate risk of bias) [34, 35]. Compared to usual care, the nurse-led Guided Care [32] and Community Connections Program [51] also significantly improved overall patient experiences with the quality of their care (low-moderate risk of bias). Only one study evaluated the impact of team-based system navigation on patient experiences; the Health TAPESTRY program did not significantly improve patient experiences (i.e., level of difficulty accessing healthcare resources, care comprehensiveness, patient-centeredness, satisfaction) when compared to usual care (low risk of bias) [33].

#### Caregiver outcomes

Caregiver experience and health outcomes were reported in two studies that investigated health professional-led system navigation models [32, 51]. Overall, the findings were unclear. Compared to usual care, caregiver experiences (i.e., perception of patient care quality) improved after the nurse-led Guided Care intervention (moderate risk of bias) [32] but not after the nurse-led Community Connections Program (low risk of bias) [51]. Evidence from only one study demonstrated no impact of the nurse-led Guided Care intervention on caregiver strain and depression (moderate risk of bias) [32] (Additional file 8).

#### Cost-related outcomes

Only two studies reported on cost-related outcomes; both evaluated a lay person-led system navigation model [44, 48]. The cost of emergency department/hospital visits and emergency care per patient were compared to costs in a matched control group in one study (moderate risk of bias) [48] and projected annual cost savings based on mathematical modelling in another (low risk of bias) [44]. Although both studies reported differences between groups, no formal statistical tests were reported (Additional file 9).

## Discussion

Building upon a previous scoping review, this systematic review synthesizes a growing body of evidence regarding the effectiveness of system navigation programs linking primary care with community-based health and social services. Whereas 1,248 records were screened in the original review, our search identified 15,226 new studies published since 2013, suggesting a substantial increase in interest in this field. Overall, there was variation in impacts across models of system navigation programs linking primary care with community-based health and social services on patient, caregiver, and health system outcomes. Evidence from three studies with low risk of bias [33, 41, 47] suggests a team-based system navigation approach may result in slightly more appropriate health service utilization (e.g., increases in primary care use versus use of costlier health services) compared to baseline or usual care. These results may indicate a shift from reactive to more preventative care and self-management support, with health and social needs being better managed at the most appropriate level of care. Evidence from four studies [32, 34, 35, 51] with moderate risk of bias suggests either lay person-led or health professional-led system navigation models may improve patient experiences with the quality of care when compared to usual care. This is consistent with patient descriptions of such programs as empowering, generally meeting their identified needs, and allowing patients to form positive relationships with their healthcare providers [60]. It is unclear whether system navigation may improve patient-related outcomes (e.g., health-related quality of life, mental health and wellbeing, health behaviours). The evidence is very uncertain about the effect of system navigation programs on caregiver and cost-related outcomes as these were evaluated in a small number of studies. Although promising trends were observed, the potential impacts of lay person-led system navigation models on cost-related outcomes are unclear due to limited data, heterogeneous outcome measurements, and a lack of reporting concerning statistical significance.

Our findings are consistent with those of another systematic review that demonstrated inconsistent effects of social prescribing programs in the United Kingdom on healthcare usage outcomes, generally consistent improvements in patient experiences, and limited evidence on costs [61]. Also consistent with our findings, a recent mixed methods systematic review identified variable effectiveness of social prescribing services on health, wellbeing, health-related behaviours, self-confidence, social isolation/loneliness, and daily functioning [62]. Although qualitative findings demonstrated that social prescribing service users generally experienced positive improvements in health/wellbeing and health behaviours, this was not consistently demonstrated by quantitative measures [62], in line with the patient-related findings in our review.

Heterogeneous measurements across patient-related outcomes may explain some of the variation in findings within this category. Further, the presence of wide confidence intervals for many effect measures suggests that small sample sizes may have contributed to the lack of significant findings observed. While it is possible that quantitative measurements alone are insufficient to capture the holistic impact of system navigation, it is also conceivable that interventions focused primarily on linking patients to existing community-based health and social services may be insufficient to influence significant changes in patient-related health and health behaviour outcomes. For example, evidence from a recent systematic review demonstrates that chronic disease/case management and disease prevention initiatives led by registered nurses in primary care settings are effective for improving health outcomes and health-related behaviours such as weight loss, smoking cessation, diet and physical activity, self-efficacy, and social activity [63]. Thus, while team-based system navigation may be effective for improving health service utilization by supporting patients to access the most appropriate services to meet their needs, the lack of clinical care provision within system navigation programs, when compared to primary care-based chronic disease and/or case management interventions [27], may limit the possible impact of system navigation alone on health-related outcomes.

Several studies in this systematic review focused on populations who may face structural barriers to accessing care and found generally positive results. This included patients experiencing social isolation and/or chronic conditions with high use of primary care [41, 42, 47, 51], individuals managing a chronic condition with previously limited engagement with their primary care team [52], patients with multiple chronic conditions living in high-poverty areas [34, 35], and those deemed to be at high risk for avoidable and costly health services use due to medical or psychosocial conditions [32, 44]. These findings suggest that the greatest impacts of system navigation programs may be observed among populations who stand to benefit the most from improved connections to community-based health and social services. This hypothesis is supported by existing evidence that patients with chronic conditions, unmanaged behavioural health needs, and those experiencing health inequities (e.g., poverty, limited social support) tend to be the highest drivers of potentially avoidable and costly health services use [64, 65]. Further research is needed to identify which populations may benefit the most from system navigation.

Several limitations should be considered when interpreting the results of this review. Although the individual studies within the review were appraised as having a generally low to moderate risk of bias, it is important to note that most were quasi-experimental, therefore lacking randomized controlled groups to facilitate strong comparisons. Further, most studies took place in the United States of America or the United Kingdom, which may limit generalizability to other health and social care contexts. Challenges with outcome measurements in the included studies also limited our conclusions. Although the primary outcomes of interest were access to care and health and social service utilization, none of the included studies objectively measured access to care or social service use outcomes, making it difficult to determine intervention effectiveness. For example, while changes in health services utilization were observed in several studies, we cannot definitively say that this was a direct result of increased connections to community-based social services because outcomes were typically only measured in the primary and/or acute care sectors. Another recent systematic review of social prescribing interventions identified similar limitations when analyzing the available evidence, suggesting that it is important to assess community-level changes (e.g., social service use, belonging, social support) and their associated impacts on health services use [66]. Finally, given the generally small number of studies per outcome and high heterogeneity in results, our certainty regarding the effectiveness of system navigation programs on user and health system outcomes is low. The number of intervention studies has notably increased since the original scoping review, in which most studies were descriptive in nature. As more high-quality data becomes available regarding system navigation programs linking primary care with community-based health and social services, more robust and definitive conclusions may be observed.

### Implications for research

Our synthesis of the effectiveness of system navigation programs, alongside existing synthesized evidence regarding social prescribing services [62], suggests that the potential impacts of these types of interventions may not be adequately captured through quantitative measurement tools alone. Although the decision to limit included studies to experimental and quasi-experimental designs was justified based on the objective of this systematic review to determine intervention effectiveness, future review authors may want to consider alternate research questions and types of evidence syntheses (e.g., integrative review, realist review) that would allow for the inclusion of both qualitative and quantitative data. This may also help determine the acceptability and feasibility of system navigation programs, given the generally high loss to follow up observed across studies (Table 1) and the lack of reporting concerning intervention adherence and fidelity (Table 2). Although we did not review qualitative data when studies used mixed methods, which may be a limitation, less than one quarter (*n* = 5) [40, 41, 43, 46, 47] of included studies conducted mixed methods evaluations.

While only one study evaluated a self-navigation model by providing individuals with a personalized list of local services with lay support available [50], further research is warranted to evaluate similar novel approaches to system navigation. Researchers should ensure appropriate facilitation and support are available when designing self-navigation interventions, as this is known to be key for overcoming fluctuating health status concerns in persons managing chronic conditions or challenges with health literacy [67]. Our review also highlights a need for more research related to the impact of system navigation programs on caregiver and cost-related outcomes. Although this review focused on patients’ and caregivers’ perspectives, it would be salient for future research to also consider the health professional perspective, given the rising levels of burnout and strain reported among primary care providers [68].

### Implications for practice

Assisting patients and families to navigate and access programs and services is a current mandate for primary care providers [69]. Integration of system navigation within primary care settings is proposed as a potential approach to alleviate some of the current and projected demands on the primary care sector [70]. Providers should consider prioritizing individuals at greater risk for potentially avoidable and costly health services use when implementing system navigation programs. Findings from this review suggest that persons managing chronic conditions, experiencing social isolation, and/or living with health inequities (e.g., low income) may stand to benefit the most from navigation support, although further research is warranted. While this review included adults aged 18 + , the median age of 72 years across included studies also suggests that older adults are key targets for system navigation support, consistent with the complex, multimorbid health and social conditions older adults often face [71, 72].

### Implications for policy

Given the current orientation of health systems toward delivering integrated and coordinated health and community services [73, 74], this systematic review is also highly relevant to policy makers. We identified system navigation models that may support outcomes relevant to the Quintuple Aim framework for healthcare improvement [75, 76], which is top of mind for decision makers to advance health equity and improve patient and provider experiences, health system utilization, and cost-effectiveness. Our findings highlight the potential benefit of team-based system navigation as a strategy to improve use of primary healthcare services versus costlier healthcare (e.g., emergency department visits, hospitalizations) and enhance patient experiences with care.

## Conclusion

System navigation programs linking primary care with community-based health and social services demonstrated mixed results. The ideal model of system navigation for improving patient, caregiver, and health system outcomes remains unclear. Nevertheless, a multidisciplinary team of healthcare providers and lay persons performing system navigation activities within primary care settings may result in slightly more appropriate health service utilization. Lay person-led or health professional-led system navigation may improve patient experiences with quality of care. Further research is warranted, specifically to understand the impact of system navigation on caregiver and cost-related outcomes, and to identify which populations may benefit the most from integrated health and social service care delivery programs.

## Supplementary Information

**Additional file 1.** Search Strategies.

**Additional file 2.** List of Excluded Studies.

**Additional file 3.** JBI Critical Appraisal Checklist for Randomized Controlled Trials.

**Additional file 4.** JBI Critical Appraisal Checklist for Quasi-Experimental Studies.

**Additional file 5.** Health Service Utilization Outcomes.

**Additional file 6. **Patient-Related Outcomes.

**Additional file 7.** Patient Experience Outcomes.

**Additional file 8. **Caregiver Outcomes.

**Additional file 9. **Cost-Related Outcomes.

## Data Availability

All data generated or analysed during this study are included in this published article and its supplementary information files.

## Abbreviations

IMPaCT: Individualized Management for Patient-Centered Targets program
RCT: Randomized controlled trial
SF-12, SF-36: 12- Or 36-Item Short Form Survey
TIDieR: Template for Intervention Description and Replication
