# Supplementary material for: Effectiveness of system navigation programs linking primary care with community-based health and social services: a systematic review
Source: BMC Health Serv Res. 2023 May 8;23:450. doi: 10.1186/s12913-023-09424-5 (PMC10165767; doi:10.1186/s12913-023-09424-5)
Supplement: Supplementary file 1 — Additional file 1. Search Strategies. [file 12913_2023_9424_MOESM1_ESM.docx]

# **Additional file 1: Search Strategies**

**Database: OVID Medline Epub Ahead of Print, In-Process & Other Non-Indexed Citations, Ovid MEDLINE(R) Daily and Ovid MEDLINE(R) 1946 to Present**

1. continuity of patient care.mp. or “Continuity of Patient Care”/
2. patient navigation.mp or Patient Navigation/
3. case management.mp. or Case Management/
4. ((care or coach* or service* or system* or healthcare or health care) adj (coordinat* or facilitat* or navigat* or transition*)).mp. [mp=title, abstract, original title, name of substance word, subject heading word, floating sub-heading word, keyword heading word, organism supplementary concept word, protocol supplementary concept word, rare disease supplementary concept word, unique identifier, synonyms]
5. (guided adj care).mp [mp=title, abstract, original title, name of substance word, subject heading word, floating sub-heading word, keyword heading word, organism supplementary concept word, protocol supplementary concept word, rare disease supplementary concept word, unique identifier, synonyms]
6. (case adj manage*).mp [mp=title, abstract, original title, name of substance word, subject heading word, floating sub-heading word, keyword heading word, organism supplementary concept word, protocol supplementary concept word, rare disease supplementary concept word, unique identifier, synonyms]
7. post-discharge support.mp.
8. social prescri*.mp.
9. nurse navgiat*.mp.
10. 1 or 2 or 3 or 4 or 5 or 6 or 7 or 8 or 9
11. family practice.mp. or Family Practice
12. general practice.mp. or General Practice/
13. primary health care.mp. or Primary Health Care/
14. public health.mp. or Public Health/
15. Community Health Centers/ or community health centre.mp.
16. Community Health Services/ or community health service.mp.
17. (aboriginal health cent* or community health cent* or CLSCs or centre local de services communautaires or community health service* or family doctor or family health or family health organization or family health team or family practitioner or family medicine or family physician or family practice or family practise or general practice or general practise or general practitioner* or group health or group practise or group practice or guided care or local community service centre or medical home or nurse practitioner led clinic* or outpost nursing station or patient centred care or primary care physician or primary care or primary care network* or primary nursing or primary health care or primary healthcare or primary health service or primary medical care).mp.
18. 11 or 12 or 13 or 14 or 15 or 16 or 17
19. 10 and 18
20. Limit 19 to yr=”2013-Current”

**Database: PsychINFO**

1. “Continuum of Care”/ or continuum of care.mp.
2. Integrated services/ or integrated services.mp.
3. patient navigat*.mp.
4. case management/ or case manage*.mp.
5. ((care or coach* or service* or system* or health care or healthcare) adj (coordinat* or facilitat* or navigat* or transition*)).mp.
6. (guided adj care).mp.
7. (case adj manage*).mp.
8. post-discharge support.mp.
9. social prescri*.mp.
10. nurse navgiat*.mp.
11. 1 or 2 or 3 or 4 or 5 or 6 or 7 or 8 or 9 or 10
12. family medicine/
13. primary health care/
14. public health/ or public heart.mp.
15. (aboriginal health cent* or community health cent* or CLSCs or centre local de services communautaires or community health service* or family doctor or family health or family health organization or family health team or family practitioner or family medicine or family physician or family practice or family practise or general practice or general practise or general practitioner* or group health or group practise or group practice or guided care or local community service centre or medical home or nurse practitioner led clinic* or outpost nursing station or patient centred care or primary care physician or primary care or primary care network* or primary nursing or primary health care or primary healthcare or primary health service or primary medical care).mp.
16. 12 or 13 or 14 or 15
17. 11 and 16
18. Limit 17 to yr=”2013-Current”

**Database: EMBASE**

1. patient care planning.mp. or patient care planning/
2. patient navigat*.mp.
3. case management/ or care manage*.mp.
4. continuity or patient care.mp.
5. ((care or coach* or service* or system* or health care or healthcare) adj (coordinat* or facilitat* or navigat* or transition*)).mp. [mp=title, abstract, heading word, drug trade name, original title, device manufacturer, drug manufacturer, device trade name, keyword, floating subheading word, candidate term word]
6. (guided adj care).mp. [mp=title, abstract, heading word, drug trade name, original title, device manufacturer, drug manufacturer, device trade name, keyword, floating subheading word, candidate term word]
7. social prescrip*.mp.
8. nurse navigat*.mp.
9. post-discharge support.mp.
10. 1 or 2 or 3 or 4 or 5 or 6 or 7 or 8 or 9
11. general practice/ or general practi*.mp. or general practitioner/
12. family practi*.mp.
13. primary health care.mp. or primary health care/
14. public health.mp. or public health/
15. community health cent*.mp.
16. community care/ or community health service*.mp.
17. (aboriginal health cent* or community health cent* or CLSCs or centre local de services communautaires or community health service* or family doctor or family health or family health organization or family health team or family practitioner or family medicine or family physician or family practice or family practise or general practice or general practise or general practitioner* or group health or group practise or group practice or guided care or local community service centre or medical home or nurse practitioner led clinic* or outpost nursing station or patient centred care or primary care physician or primary care or primary care network* or primary nursing or primary health care or primary healthcare or primary health service or primary medical care).mp.
18. 11 or 12 or 13 or 14 or 15 or 16 or 17
19. 10 and 18
20. Limit 19 to yr=”2013-Current”

**Databse: CINAHL**

1. (MH “Continuity of Patient Care”) OR “continuity of care”
2. (MH “Patient Navigation”) OR “patient navigat*”
3. “system navigat*”
4. (MH “Case Management”) OR (MH “Case Managers”) OR “case manage*”
5. coordinat* N0 (care or coach* or service* or system* or healthcare or health care)
6. facilitat* N0 (care or coach* or service* or system* or healthcare or health care)
7. navigat* N0 (care or coach* or service* or system* or healthcare or health care)
8. transition* N0 (care or coach* or service* or system* or healthcare or health care)
9. guided N0 care
10. “post-discharge support”
11. S1 OR S2 OR S3 OR S4 OR S5 OR S6 OR S7 OR S8 OR S9 OR S10
12. “aboriginal health care”
13. (MH “community Health Centers”) OR “community health centre”
14. “CLSC”
15. “centre local de services communautaires”
16. (MH “Community Health Services”) OR “community health services”
17. “family doctor”
18. “family health organization”
19. (MH “Multidisciplinary Care Team”) OR “family health team”
20. (MF “Family Nurse Practitioners”) OR “family practitioner”
21. (MH “Physicians, Family”) OR “family physician”
22. (MH “Family Practice”) OR “family practice”
23. “family practise”
24. “general practi*”
25. (MH “Group Practice”) OR “group practi*”
26. “guided care”
27. (MH “Public Health”)
28. “social prescri*”
29. S11 or S28
30. S12 OR S13 OR S14 OR S15 OR S16 OR S17 OR S18 OR S19 OR S20 OR S21 OR S22 OR S23 OR S24 OR S25 OR S26 OR S27
31. S29 AND S30
32. S29 AND S30 – Limiters – Published Date 20130101-20201231

**Database: Cochrane Central Register of Controlled Trials**

1. MeSH descriptor: [Continuity of Patient Care] this term only
2. continuity of patient care
3. MeSH description: [Patient Navigation] this term only
4. patient navigat*
5. MeSH descriptor: [Case Management] in all MeSH products
6. case manage*
7. ((care or coach* or service* or system* or health care or healthcare) NEAR/1 (coordinat* or facilitate* or navigat* or transition*))
8. guided NEAR/1 care
9. case NEAR/1 manage*
10. post-discharge support
11. social prescri*
12. nurse navigat*
13. #1 OR #2 OR #3 OR #4 OR #5 OR #6 OR #7 OR #8 OR #9 OR #10 OR #11 OR #12
14. MeSH description: [Family Practice] this term only
15. MeSH descriptor: [General Practice] this term only
16. MeSH descriptor: [Primary Health Care] this term only
17. MeSH descriptor: [Public Health] this term only
18. MeSH descriptor: [Community Health Services] this term only
19. MeSH descriptor: [Community Health Centers] this term only
20. (aboriginal health cent* or community health cent* or CLSCs or centre local de services communautaires or community health service* or family doctor or family health or family health organization or family health team or family practitioner or family medicine or family physician or family practice or family practise or general practice or general practise or general practitioner* or group health or group practise or group practice or guided care or local community service centre or medical home or nurse practitioner led clinic* or outpost nursing station or patient centred care or primary care physician or primary care or primary care network* or primary nursing or primary health care or primary healthcare or primary health service or primary medical care).mp.
21. #14 OR #15 OR #16 OR #17 OR #18 OR #19 OR #20
22. #13 AND #21 – Limit to 2013-Current
