# Supplementary material for: Effectiveness of system navigation programs linking primary care with community-based health and social services: a systematic review
Source: BMC Health Serv Res. 2023 May 8;23:450. doi: 10.1186/s12913-023-09424-5 (PMC10165767; doi:10.1186/s12913-023-09424-5)
Supplement: Supplementary file 2 — Additional file 2. List of Excluded Studies. [file 12913_2023_9424_MOESM2_ESM.docx]

# **Additional file 2: List of Excluded Studies**

1. Achilla E, McCrone P, Phillips R, Barley EA, Mann A, Tylee A. UPBEAT-UK: Cost-effectiveness of Nurse-Led Case Management and Usual Care for Patients with Coronary Heart Disease and Co-Morbid Depression. Journal of Mental Health Policy and Economics. 2013;16:S1.

Exclusion reason: Population

1. Aggar C, Thomas T, Gordon C, Bloomfield J, Baker J. Social Prescribing for Individuals Living with Mental Illness in an Australian Community Setting: A Pilot Study. Community Mental Health Journal. 2021;57(1):189-195.

Exclusion reason: Population

1. Alderwick HAJ, Gottlieb LM, Fichtenberg CM, Adler NE. Social Prescribing in the U.S. and England: Emerging Interventions to Address Patients' Social Needs. American Journal of Preventive Medicine. 2018;54(5):715-8.

Exclusion reason: Study design

1. Allary C, Bourmaud A, Tinquist F, Oriol M, Kalecinski J, Dutertre V, Lechopier N, Pommier M, Benoist Y, Rousseau S, Regnier V, Buthion V, Chauvin F. ColoNav: Patient Navigation for Colorectal Cancer Screening in Deprived Areas - Study protocol. BMC Cancer. 2016;16:416.

Exclusion reason: Study design

1. Allen JD, Perez JE, Tom L, Leyva B, Diaz D, Idali Torres M. A Pilot Test of a Church-Based Intervention to Promote Multiple Cancer-Screening Behaviors among Latinas. Journal of Cancer Education. 2014;29(1):136-43.

Exclusion reason: Intervention

1. Allen K, Huff NL. Family coaching: An Emerging Family Science Field. Family Relations: An Interdisciplinary Journal of Applied Family Studies. 2014;63(5):569-82.

Exclusion reason: Study design

1. Anderson JE, Larke SC. Navigating the mental health and addictions maze: a community-based pilot project of a new role in primary mental health care. Mental Health in Family Medicine. 2009;6(1):15–9.

Exclusion reason: Population

1. Anderson JE, Larke SC. The Sooke Navigator project: using community resources and research to improve local service for mental health and addictions. Mental Health in Family Medicine. 2009;6(1):21–8.

Exclusion reason: Study design

1. Anonymous. ED navigators Help Patients Find a PCP. Hospital Case Management: The Monthly Update On Hospital-Based Care Planning and Critical Paths. 2014;22(1):9-10.

Exclusion reason: Study design

1. Anonymous. Social prescribing: Community-Based Referral in Public Health. Perspectives in Public Health. 2018;138(1):18-9.

Exclusion reason: Study design

1. Aponte-Soto L. Assessing Patient Navigation and Support Services at FQHCs. Cancer Research. 2019;79.

Exclusion reason: Study design

1. Australian Clinical Trials Registry. A Person-Centred Model of Residential Respite Care Transition Support: The Transition Support-Person Centred Care (TS-PCC) Program for Family Caregivers and Care Recipients with Dementia. 2014.

Exclusion reason: Population

1. Australian Clinical Trials Registry. Evaluation of Gold Coast Integrated Care for Patients with Chronic Disease through a Non-Randomised Controlled Clinical Trial. 2016.
2. Australian Clinical Trials Registry. The Central Australian Heart Protection Study: A Randomised Trial of Nurse-Led, Family Based Secondary Prevention of Acute Coronary Syndromes. 2014.

Exclusion reason: Study design

Exclusion reason: Intervention

1. Baggett TP, Teixeira JB, Rodriguez EC, Anandakugan N, Sporn N, Chang Y, Park SPLER, Rigotti NA. Provider perceptions and costs outcomes of a community-health center based patient navigation program for lung cancer screening. 2018;Conference: 41st Annual Meeting of the Society of General Internal Medicine, SGIM 2018. United States. 33(2 Supplement 1):316.

Exclusion reason: Intervention

1. Baird M, Blount A, Brungardt S, Dickinson P, Dietrich A, Epperly T, Green L, Henley D, Kessler R, Korsen N, McDaniel S, Miller B, Pugno P, Roberts R, Schirmer J, Seymour D, Degruy F. The development of joint principles: Integrating behavioral health care into the patient-centered medical home. Families, Systems, & Health. 2014;32(2):153.

Exclusion reason: Study design

1. Bartels SJ, Aschbrenner KA, Rolin SA, Hendrick DC, Naslund JA, Faber MJ. Activating Older Adults with Serious Mental Illness for Collaborative Primary Care Visits. Psychiatric Rehabilitation Journal. 2013;36(4):278-88.

Exclusion reason: Population

1. Belmonte IA, Guanyabens IV. Social Prescribing in a Community: Do We Do it from Primary Care? Formación Médica Continuada en Atención Primaria. 2019;26(7):420-3.

Exclusion reason: Language

1. Belsher BE, Freed MC, Evatt DP, Engel CC, Liu X, Novak L, Zatzick DF. Population Impact of PTSD and Depression Care for Military Service Members: Reach and Effectiveness of an Enhanced Collaborative Care Intervention. Psychiatry: Interpersonal and Biological Processes. 2018;81(4):349-60.

Exclusion reason: Population

1. Bensink ME, Ramsey SD, Battaglia T, Fiscella K, Hurd TC, McKoy JM, Patierno SR, Raich PC, Seiber EE, Mears VW, Whitley E, Paskett ED, Mandelblatt JS. Costs and Outcomes Evaluation of Patient Navigation After Abnormal Cancer Screening: Evidence from the Patient Navigation Research Program. Cancer. 2014;120(4):570-8.

Exclusion reason: Intervention

1. Berkowitz SA, Parashuram S, Rowan K, Andon L, Bass EB, Bellantoni M, Brotman DJ, Deutschendorf A, Dunbar L, Durso SC, Everett A, Giuriceo KD, Hebert L, Hickman D, Hough DE, Howell EE, Huang X, Lepley D, Leung C, Lu Y, Lyketsos CG, Murphy SME, Novak T, Purnell L, Sylvester C, Wu AW, Zollinger R, Koenig K, Ahn R, Rothman RB, Brown PMC. Association of a Care Coordination Model With Health Care Costs and Utilization: The Johns Hopkins Community Health Partnership (J-CHiP). JAMA Network Open. 2018;1(7).

Exclusion reason: Intervention

1. Berkowitz SA, Percac-Lima S, Ashburner JM, Chang Y, Zai AH, He W, Grant RW, Atlas SJ. Building Equity Improvement into Quality Improvement: Reducing Socioeconomic Disparities in Colorectal Cancer Screening as Part of Population Health Management. Journal of General Internal Medicine. 2015;30(7):942-9.

Exclusion reason: Intervention

1. Bertotti M, Frostick C, Hutt P, Sohanpal R, Carnes D. A Realist Evaluation of Social Prescribing: An Exploration into the Context and Mechanisms Underpinning a Pathway Linking Primary Care with the Voluntary Sector. Primary Health Care Research & Development. 2018;19(3):232-45.

Exclusion reason: Study design

1. Berz J, Quintiliani LM, Truong V, Murillo J, Lasser KE. Implementation Strategies to Increase Use of Community-Based Diabetes Prevention Programs by Primary Care Patients at an Urban Safety-Net Hospital. Journal of General Internal Medicine. 2019;34.

Exclusion reason: Population

1. Bharmal N, Clarke R, Di Capua P, Gupta I, Doyle B, Ali A, Malim A, Mittman BS. Development and Application of a Classification Scheme for Care Coordination Activities in an Academic Primary Care System. Journal of General Internal Medicine. 2014;29.

Exclusion reason: Intervention

1. Bickerdike L, Booth A, Wilson PM, Farley K, Wright K. Social Prescribing: Less Rhetoric and More Reality. A Systematic Review of the Evidence. BMJ Open. 2017;7(4).

Exclusion reason: Study design

1. Bishop SE, Edwards JM, Nadkarni M. Charlottesville Health Access: a locality-based model of health care navigation for the homeless. Journal of Health Care for the Poor & Underserved. 2009;20(4):958–63.

Exclusion reason: Study design

1. Bodenmann P, Velonaki VS, Baggio S, Iglesias K, Moschetti K, Ruggeri O. Frequent Users of the Emergency Department in a Universal Health Coverage System: A Randomized Controlled Trial of a Case-Management Intervention. Journal of General Internal Medicine. 2015;30.

Exclusion reason: Intervention

1. Bodenmann P, Velonaki VS, Griffin JL, Baggio S, Iglesias K, Moschetti K. Case Management May Reduce Emergency Department Frequent Use in a Universal Health Coverage System: A Randomized Controlled Trial. Journal of General Internal Medicine. 2017;32(5):508-15.

Exclusion reason: Wrong setting

1. Bodenmann P, Velonaki VS, Ruggeri O, Hugli O, Burnand B, Wasserfallen JB. Case Management for Frequent Users of the Emergency Department: Study Protocol of a Randomised Controlled Trial. BMC Health Services Research. 2014;14:264.

Exclusion reason: Intervention

1. Bohman TM, Wallisch L, Christensen K, Stoner D, Pittman A, Reed B, Ostermeyer B. Working Well – The Texas Demonstration to Maintain Independence and Employment: 18-month outcomes. Journal of Vocational Rehabilitation. 2011;34:97-106.

Exclusion reason: Population

1. Boult C, Wieland GD. Comprehensive primary care for older patients with multiple chronic conditions: “Nobody rushes you through”. The Journal of the American Medical Association. 2010;304(17):1936–43.

Exclusion reason: Study design

1. Boyd CM, Boult C, Shadmi E, Leff B, Brager R, Dunbar L, Wolff JL, Wegener S. Guided care for multimorbid older adults. Gerontologist. 2007;47(5):697–704.

Exclusion reason: Wrong outcomes

1. Bozorgmehr K, Szecsenyi J, Ose D, Besier W, Mayer M, Krisam J, Jacke CO, Salize H, Brandner R, Schmitt S, Kiel M, Kamradt M, Freund T. Practice Network-Based Care Management for Patients with Type 2 Diabetes and Multiple Comorbidities (GEDIMAt plus): Study Protocol for a Randomized Controlled Trial. Trials. 2014;15:243.

Exclusion reason: Population

1. Bradford JB, Coleman S, Cunningham W. HIV System Navigation: an emerging model to improve HIV care access. AIDS Patient Care & STDs. 2007;21:S–58.

Exclusion reason: Study design

1. Braun KL, Thomas WL, Jr., Domingo JL, Allison AL, Ponce A, Haunani Kamakana P, Brazzel SS, AluliNE, Tsark JU. Reducing Cancer Screening Disparities in Medicare Beneficiaries through Cancer Patient Navigation. Journal of the American Geriatrics Society. 2015;63(2):365-70.

Exclusion reason: Population

1. Bravin JI, Anderson ML, Clark T, Savin KL, Ledesma DL, Euyoque JA, Fortmann AL, Tsimikas A, Gallo LC. Mi Puente: A Care Transitions Intervention for At-Risk Hispanics with Multiple Cardiometabolic Conditions. Trials. 2019;68.

Exclusion reason: Intervention

1. Brinker N. Delivering Meaningful Care for All: The Promise of Patient Navigation. Journal of Oncology Navigation & Survivorship. 2020;11(3):82-3.

Exclusion reason: Study design

1. Brown N, Vaughn NA, Lin AJ, Browne R, White M, Smith P. Healthy families Brooklyn: working with health advocates to develop a health promotion program for residents living in New York City housing authority developments. Journal of Community Health. 2011;36(5):864.

Exclusion reason: Study design

1. Burhansstipanov L. The Art of Community-Based Patient Navigation. Journal of Oncology Navigation & Survivorship. 2017;8(8):370-4.

Exclusion reason: Study design

1. Burton J, Murphy E, Riley P. Primary immunodeficiency disease: a model for case management of chronic diseases. Professional case management. 2010;15(1):5–6.

Exclusion reason: Study design

1. Cabassa LJ, Manrique Y, Meyreles Q, Capitelli L, Younge R, Dragatsi D, Alvarez J, Lewis-Fernandez R. “Treated me ... like I was family”: Qualitative Evaluation of a Culturally Adapted Health Care Manager Intervention for Latinos with Serious Mental Illness and at Risk for Cardiovascular Disease. Transcultural Psychiatry. 2019;56(6):1218-36.

Exclusion reason: Study design

1. Caldwell R, Eberth J, Thibault A, Seabrook M, Berger F. A High-Quality Colonoscopy Screening Program for the Uninsured and Medically Underserved in South Carolina. The American Journal of Gastroenterology. 2015;110:S595-628.

Exclusion reason: Study design

1. Calhoun EA, Pauls H, Vijayasiri G, Darnell JS, Molina Y, Berrios N. Patient Navigation in Medically Underserved Areas. Cancer Epidemiology Biomarkers and Prevention. 2015;24.

Exclusion reason: Population

1. Campbell JW, Wank D, Campbell P, Crider P. Geriatric principles promote cost saving in ACOa. Journal of the American Geriatrics Society. 2018;66(S2):S211.

Exclusion reason: Study design

1. Canada RE, Klusaritz H, Garland JM, Medina J, Van Decker SG, Nivasch E. The Complex Care Program: Navigation and Advocacy for Medically Complicated Undocumented Latinos at Puentes de Salud. Journal of General Internal Medicine. 2016;31.

Exclusion reason: Study design

1. Capp R, Misky GJ, Honigman B, Logan H, Hardy R, Nguyen DQ, Wiler JL. Coordination Program Reduced Acute Care Use and Increased Primary Care Visits Among Frequent Emergency Care Users. Health Affairs. 2017;36(10):1705-11.

Exclusion reason: Population

1. Carrasquillo O, McCann S, Amofah A, Pierre L, Rodriguez B, Alonzo Y, Ilangovan K, Gonzalez M, Trevil D, Byrne MM, Koru-Sengul T, Kobetz E. Rationale and Design of the Research Project of the South Florida Center for the Reduction of Cancer Health Disparities (SUCCESS): Study Protocol for a Randomized Controlled Trial. Trials. 2014;15(1):299.

Exclusion reason: Population

1. Carroll JK, Humiston SG, Meldrum SC, Salamone CM, Jean-Pierre P, Epstein RM, Fiscella K. Patients’ experiences with navigation for cancer care. Patient Education & Counseling. 2010;80(2):241–7.

Exclusion reason: Study design

1. Carter J, Walton A, Donelan K, Thorndike A. Implementing Community Health Worker-Patient Pairings at the Time of Hospital Discharge: A Randomized Control Trial. Contemporary Clinical Trials. 2018;74:32‐7.

Exclusion reason: Intervention

1. Carter N, Valaitis RK, Lam A, Feather J, Nicholl J, Cleghorn L. Navigation Delivery Models and Roles of Navigators in Primary Care: A Scoping Literature Review. BMC Health Services Research. 2018;18(1):2-13

Exclusion reason: Study design

1. Cavanagh MF, Lane DS, Messina CR, Anderson JC. Clinical Case Management and Navigation for Colonoscopy Screening in an Academic Medical Center. Cancer. 2013;119(S15):2894-2904.

Exclusion reason: Intervention

1. Chatterjee H, Polley M, Clayton G. Social Prescribing: Community-Based Referral in Public Health. Perspectives in Public Health. 2018;138(1):18-9.

Exclusion reason: Study design

1. Cheng Q, Kinner SA, Lee XJ, Snow KJ, Graves N. Cost-Utility Analysis of Low-Intensity Case Management to Increase Contact with Health Services among Ex-Prisoners in Australia. BMJ Open. 2018;8(8):1-8.

Exclusion reason: Wrong setting

1. Chouinard MC, Hudon C, Dubois MF, Roberge P, Loignon C, Tchouke, Fortin M, Couture, Sasseville M. Case Management and Self-Management Support for Frequent Users with Chronic Disease in Primary Care: A Pragmatic Randomized Controlled Trial. BMC Health Services Research. 2013;13:1-13.

Exclusion reason: Intervention

1. Chow SK, Wong FK. A Randomized Controlled Trial of a Nurse-Led Case Management Programme for Hospital-Discharged Older Adults with Comorbidities. Journal of Advanced Nursing. 2014;70(10):2257-71.

Exclusion reason: Intervention

1. Cirlugea O, Ta J. Chronic Disease Management and Integrated Care Among Hispanic Populations. Enhancing Behavioral Health in Latino Populations. 2016;77(1):267-95.

Exclusion reason: Study design

1. Clark CR, Baril N, Kunicki M, Johnson N, Soukup J, Ferguson K, Lipsitz S, Bigby J. Addressing social determinants of health to improve access to early breast cancer detection: results of the Boston REACH 2010 Breast and Cervical Cancer Coalition Women’s Health Demonstration Project. Journal of Women’s Health. 2010;18(5):677–90.

Exclusion reason: Population

1. Clark S. Acceptability And Feasibility Of A Multicomponent Group Intervention To Initiate Health Behavior Change: The Kickstart Health Program. Wayne State University. 2020;81.

Exclusion reason: Intervention

1. Cole H, Thompson HS, White M, Browne R, Trinh-Shevrin C, Braithwaite S, Fiscella K, Boutin-Foster C, Ravenell J. Community-Based, Preclinical Patient Navigation for Colorectal Cancer Screening Among Older Black Men Recruited from Barbershops: The MISTER B Trial. American Public Health Association. 2017;107(9):1433-40.

Exclusion reason: Intervention

1. Collinsworth AW, Vulimiri M, Schmidt KL, Snead CA. Effectiveness of a Community Health Worker-Led Diabetes Self-Management Education Program and Implications for CHW Involvement in Care Coordination Strategies. The Science of Diabetes Self-Management and Care. 2013;39(6):792-9.

Exclusion reason: Population

1. Connor KI, Cheng EM, Barry F, Siebens HC, Lee ML, Ganz DA, Mittman BS, Connor MK, Edwards LK, McGowan MG, Vickrey BG. Randomized Trial of Care Management to Improve Parkinson Disease Care Quality. American Academy of Neurology. 2019;92(16).

Exclusion reason: Intervention

1. Coronado GD, Johnson ES, Leo MC, Schneider JL, Smith D, Mummadi R, Petrik AF, Thompson JH, Jimenez R. Patient Randomized Trial of a Targeted Navigation Program to Improve Rates of Follow-Up Colonoscopy in Community Health Centers. Contemporary Clinical Trials. 2020;89.

Exclusion reason: Study design

1. Corrigan PW, Kraus DJ, Pickett SA, Schmidt A, Stellon E, Hantke E, Lara JL. Using Peer Navigators to Address the Integrated Health Care Needs of Homeless African Americans With Serious Mental Illness. Psychiatric Services. 2017;68(3):264-70.

Exclusion reason: Population

1. Corrigan PW, Pickett S, Schmidt A, Stellon E, Hantke E, Kraus D, Dubke R. Peer Navigators To Promote Engagement Of Homeless African Americans With Serious Mental Illness In Primary Care. Psychiatry Research. 2017;255:101-3.

Exclusion reason: Population

1. D'Ambrosio JG, Faul AC, Furman CD, Yankeelov PA. New Zealand Rheumatology Association and Australian Rheumatology Association with the Rheumatology Health Professionals Association Joint Annual Scientific Meeting. Internal Medicine Journal. 2017;47(S2):5-41.

Exclusion reason: Study design

1. Dahlman D, Palm A, Sunesdotter C, Troberg K, Wallin C. Low-Threshold Primary Care for Patients in Opiate Maintenance Therapy: A Pilot Project in Malmo, Sweden, Integrates Primary Care and OMT. Lakartidningen. 2016;113

Exclusion reason: Language

1. Dahrouge S, Gauthier A, Chiocchio F, Presseau J, Kendall C, Lemonde M. Access to Resources in the Community Through Navigation: Protocol for a Mixed-Methods Feasibility Study. JMIR Research Protocols. 2019;8(1):e11022.

Exclusion reason: Study design

1. Daumit GL, Stone EM, Kennedy-Hendricks A, Choksy S, Marsteller JA, McGinty EE. Care Coordination and Population Health Management Strategies and Challenges in a Behavioral Health Home Model. Medical Care. 2019;57(1):79-84.

Exclusion reason: Population

1. Davey A. Social Prescribing and Pre-Operative Care. Anaesthesia. 2018;73(2):256-7.

Exclusion reason: Study design

1. Davis E, Janssen J, Johnson A, Fontil V, Aronson L, Handley MA. Primary Care-Based Complex Care Management: Using An Interdisciplinary Geriatric Approach To Improve Care For Frequently Admitted Patients In A Safety Net Primary Care Clinic. Journal of General Internal Medicine. 2013;28(S1):S445.

Exclusion reason: Study design

1. Davis KM, Eckert MC, Shakib S, Harmon J, Hutchinson AD, Sharplin G, Caughey GE. Development and Implementation of a Nurse-Led Model of Care Coordination to Provide Health-Sector Continuity of Care for People With Multimorbidity: Protocol for a Mixed Methods Study. JMIR Research Protocols. 2019;8(12):15006.

Exclusion reason: Study design

1. De Stampa M, Bagaragaza E, Herr M, Aegerter P, Vedel I, Bergman H, Ankri J. Use of social and health primary care services for older people with complex needs: Comparison of three types of gerontological coordination. Revue d’Epidemiologie et de Sante Publique. 2014;62(5):315-22.

Exclusion reason: Language

1. Dean G, Haywood M. Navigation & Case Management Supporting General Practices in the Eastern Bay of Plenty, New Zealand. International Journal of Integrated Care. 2018;18:1-2.

Exclusion reason: Study design

1. DeGroff A, Coa K, Morrissey KG, Rohan E, Slotman B. Key Considerations In Designing A Patient Navigation Program For Colorectal Cancer Screening. Health Promotion Practice. 2014;15(4):483-95.

Exclusion reason: Study design

1. DeGroff A, Gressard L, Glover-Kudon R, Rice K, Tharpe FS, Escoffery C, Gersten J, Butterly L. Assessing the Implementation of a Patient Navigation Intervention For Colonoscopy Screening. BMC Health Services Research. 2019;19(1):803.

Exclusion reason: Intervention

1. DeGroff A, Schroy PC, Morrissey KG, Slotman B, Rohan EA, Bethel J, Murillo J, Ren W, Niwa S, Leadbetter S, Joseph D. Patient Navigation for Colonoscopy Completion: Results of an RCT. American Journal of Preventive Medicine. 2017;53(3):363-72.

Exclusion reason: Intervention

1. Dharamshi R. The Bridport Project - Integrated Community Services for Frail, Elderly Patients in West Dorset. Age and Ageing. 2017;46(1)1-22.

Exclusion reason: Study design

1. Dhruva SS. Implementation of an EConsult System with Patient Navigation. Journal Of Health Care For The Poor And Underserved. 2019;30(1):28-39.

Exclusion reason: Study design

1. Di Capua P, Clarke R, Tseng CH, Wilhalme H, Sednew R, McDonald KM, Skootsky SA, Wenger N. The Effect Of Implementing A Care Coordination Program On Team Dynamics And The Patient Experience. The American Journal of Managed Care. 2017;23(8):494-500.

Exclusion reason: Intervention

1. Doblecki-Lewis S, Butts S, Botero V, Klose K, Cardenas G, Feaster D. A Randomized Study of Passive versus Active PrEP Patient Navigation for a Heterogeneous Population at Risk for HIV in South Florida. Journal of the International Association of Providers of AIDS Care. 2019;18.

Exclusion reason: Population

1. Dominguez A, Jr. Decreasing Primary-Care-Related Emergency Department Visits In The Hispanic Population Using Patient Navigators. College of Health Sciences. 2018;79.

Exclusion reason: Wrong setting

1. Dowden A. How social prescribing can benefit patients and prescribers. Prescriber. 2019;30(4):21-4.

Exclusion reason: Study design

1. Dowden A. How Social Prescribing Can Benefit Patients And Prescriber. Prescriber. 2019;30(4):21-4.

Exclusion reason: Duplicate

1. Doyle D, Emmett M, Crist A, Robinson C, Grome M. Improving the Care of Dual Eligible Patients in Rural Federally Qualified Health Centers: The Impact of Care Coordinators and Clinical Pharmacists. Journal of Primary Care & Community Health. 2016;7(2):118-21.

Exclusion reason: Population

1. Drennan V. Evaluating The Use of Social Prescribing Coordinators in General Practices. Primary Health Care. 2018;28(1):13.

Exclusion reason: Study design

1. Drinkwater C, Wildman J, Moffatt S. Social prescribing. BMJ. 2019;364.

Exclusion reason: Intervention

1. Duarte-Climates G, Sanchez-Gomez MB, Rodriguez-Gomez JA, Rodriguez-Alvarez C, Sierra-Lopez A, Aguirre-Jaime A, Gomez-Salgado J. Impact Of The Case Management Model Through Community Liaison Nurses. International Journal Of Environmental Research And Public Health. 2019;16(11).

Exclusion reason: Study design

1. Duffin C. Assessing the Benefits Of Social Prescribing. Cancer Nursing Practice. 2016;15(2):18-20.

Exclusion reason: Study design

1. DuHamel KN, Schofield EA, Villagra C, Sriphanlop P, Itzkowitz SH, Cotter G, Cohen N, Erwin DO, Winkel G, Thompson HS, Zauber AG, Jandorf LH. Promoting Colonoscopy Screening Among Low-Income Latinos At Average Risk Of Colorectal Cancer: A Randomized Clinical Trial. Cancer. 2019;126(4).782-91.

Exclusion reason: Intervention

1. Dulaney S, Lui K, Merrilees J, Kuo A, Choi J, Ragosta M. Care Ecosystem: Care Navigation For People Living Alone With Cognitive Impairment. Alzheimer's and Dementia. 2019;15(S7):1154.

Exclusion reason: Study design

1. Dwinnells R, Misik L. An Integrative Behavioral Health Care Model Using Automated SBIRT and Care Coordination in Community Health Care. Journal of Primary Care & Community Health. 2017;8(4):300-4.

Exclusion reason: Intervention

1. Eastwood JG, Shaw M, Garg P, De Souza DE, Tyler I, Dean L, MacSween M, Moore M. Designing an Integrated Care Initiative for Vulnerable Families: Operationalisation of Realist Causal and Programme Theory, Sydney Australia. International Journal of Integrated Care. 2019;19(3):10.

Exclusion reason: Study design

1. Elseroad S, Seaberg D, Dumas M, Mendiratta S, Whittle J, Holcombe J. SAEM Annual Meeting Abstracts. Academic Emergency Medicine. 2016;23(S1):S7-S276.

Exclusion reason: Population

1. Enard KR, Ganelin DM. Reducing Preventable Emergency Department Utilization And Costs By Using Community Health Workers As Patient Navigators. Journal of Healthcare Management/American College Of Healthcare Executives. 2013;58(6).

Exclusion reason: Wrong setting

1. Eng JA, Allison TA, Kao H, Williams BA, Barnes DE, Ritchie CS. Paper Abstract. Journal of the American Geriatrics Society. 2014;62(Suppl.1):S1-S331.

Exclusion reason: Population

1. Englander H, Michaels L, Chan B, Kansagara D. The care transitions innovation (C-TraIn) for socioeconomically disadvantaged adults: results of a cluster randomized controlled trial. Journal of General Internal Medicine. 2014;29(11):1460-7.

Exclusion reason: Wrong setting

1. Ernst E. Social Prescribing. Perfusion. 2018;31(1):1.

Exclusion reason: Study design

1. Esperat M, McMurry L, Du F, Huaxin S, Billings L, Masten Y, Flores D. Transformacion para salud: a patient navigation model for chronic disease self- management. Online Journal of Issues in Nursing. 2012;17(2):1.

Exclusion reason: Study design

1. Espey D, Castro G, Flagg T, Landis K, Henderson JA, Benard VB, Royalty JE. Strengthening breast and cervical cancer control through partnerships: American Indian and Alaska Native Women and the National Breast and Cervical Cancer Early Detection Program. Cancer. 2014;120(Suppl. 16):2557-65.

Exclusion reason: Study design

1. Fang CY, Ma GX, Hahndorf EA, Feng Z, Tan Y, Rhee J, Miller SM, Kim C, Koh HS. Addressing Multi Level Barriers To Cervical Cancer Screening In Korean American Women: A Randomized Trial Of A Community-Based Intervention. Cancer. 2017;123(6):1018-26.

Exclusion reason: Intervention

1. Fernández-Moyano A, Fernandez-Ojeda R, Ruiz-Romero V, Garcia-Benitez B, Palmero-Palmero C, Aparicio-Santos R. Comprehensive Care Program For Elderly Patients Over 65 Years With Hip Fracture. Revista Clinica Espanola. 2014;214(1):17-23.

Exclusion reason: Intervention

1. Ferrante JM, Cohen DJ, Crosson JC. Translating the patient navigator approach to meet the needs of primary care. Journal of the American Board of Family Medicine. 2010;23(6):736–44.

Exclusion reason: Wrong outcomes

1. Ferrer RL, Gonzalez Schlenker C, Lozano Romero R, Poursani R, Bazaldua O, Davidson D, Gonzales MA, DeHoyos J, Castilla M, Corona BA, Tysinger J, Alsip B, Trejo J, Jaen CR. Advanced Primary Care In San Antonio: Linking Practice And Community Strategies To Improve Health. Journal of the American Board of Family Medicine. 2013;26(3):288-98.

Exclusion reason: Intervention

1. Finn M, Foster K, Gilmore ME, Goetz P, McHale B. Novice Navigator: A Case Study on the Complexities of Care Coordination. Journal of Oncology Navigation & Survivorship. 2016;7(4):31-2.

Exclusion reason: Study design

1. Fixsen A, Polley M. Social Prescribing for Stress Related Disorders and Brain Health. International Review of Neurobiology. 2020;152:237.

Exclusion reason: Intervention

1. Fleming A, Kishida MG, Kimmel CB, Keynes RJ. The CHNA: Building the Backbone of a Navigation Process. Oncology Nurse Advisor. 2016;7(3):11-2.

Exclusion reason: Study design

1. Foret Giddens J, Tanner E, Frey K, Reider L, Boult C. Expanding the gerontological nursing role in Guided Care. Geriatric Nursing. 2009;30(5):358–64.

Exclusion reason: Study design

1. Francis-Hill C, Boone M. Nurse Navigator Role in Head and Neck Cancer Community Screening. Journal of Oncology Navigation & Survivorship. 2019;10(11):440.

Exclusion reason: Duplicate

1. Fritz M, Hughes A, Woodward A, Freddolino P, Reeves MJ. The Michigan Stroke Transitions Trial: Assessment of Unmet Needs and Case Management Activities from an In-Home Patient-Centered Social Work Case Management Program. Stroke. 2017;48.

Exclusion reason: Wrong setting

1. Frostick C, Bertotti M. The Frontline Of Social Prescribing - How Do We Ensure Link Workers Can Work Safely And Effectively Within Primary Care? Chronic Illness. 2019;17(4):404-15.

Exclusion reason: Study design

1. Fujiwara M, Inagaki M, Shimazu T, Kodama M, So R, Matsushita T, et al. A Randomised Controlled Trial Of A Case Management Approach To Encourage Participation In Colorectal Cancer Screening For People with Schizophrenia in Psychiatric Outpatient Clinics: Study Protocol For The J-Support 1901 (Access) Study. BMJ Open. 2019;9(11).

Exclusion reason: Population

1. Gaglioti AH, Barlow P, Thoma KD, Bergus GR. Integrated Care Coordination By An Interprofessional Team Reduces Emergency Department Visits And Hospitalisations At An Academic Health Centre. Journal of Interprofessional Care. 2017;31(5):557-65.

Exclusion reason: Study design

1. Gerber DE, Hamann HA, Santini NO, Abbara S, Chiu H, McGuire M, Quirk L, Zhu H, Craddock SJ. Patient Navigation For Lung Cancer Screening In An Urban Safety-Net System: Protocol For A Pragmatic Randomized Clinical Trial. Contemporary Clinical Trials. 2017;60:78.

Exclusion reason: Study design

1. Gimpel N, Marcee A, Kennedy K, Walton J, Lee S, DeHaven MJ. Patient perceptions of a community-based care coordination system. Health Promotion Practice. 2010;11(2):173–81.

Exclusion reason: Study design

1. Gioia S, Torres C, Cavalcanti J, Brigagao L, Proencio T, Krush L, Goss P. The Value Of Patient Navigation In Breast Cancer Being Tested In Rio De Janeiro, Brazil. Cancer Research. 2019;79(4):S1.

Exclusion reason: Intervention

1. Gioia S, Torres C, Galdino R, Brigagao L, Heringer A, Medeiros M, Krush L, Goss PE. Bringing patient navigation to primary health care in Rio de Janeiro: Pilot project in the Andarai community. Journal of Clinical Oncology. 2019;37(S15).

Exclusion reason: Study design

1. Giunta N, Cain K. Community-Based Case Management And Health Care Use In Older Adults: Outcomes Of A Collaborative Multi Agency Approach. Care Management Journals. 2015;16(1):20-9.

Exclusion reason: Intervention

1. Glendenning D, Jones CA. New Hanover Community Paramedicine Success Story. Journal of Emergency Medical Services. 2015;40(2):59.

Exclusion reason: Study design

1. Gomez D, Bridges AJ, Andrews AR, Cavell TA, Pastrana FA, Gregus SJ, Ojeda CA. Delivering Parent Management Training In An Integrated Primary Care Setting: Description And Preliminary Outcome Data. Cognitive And Behavioral Practice. 2014;21(3):296-309.

Exclusion reason: Wrong outcomes

1. Gonzalo J, Thompson BM, Wolpaw DR. Value-added medical education: Students As Patient Navigators. Journal of General Internal Medicine. 2016;31(2):S854-S5.

Exclusion reason: Intervention

1. Goodare H. Making Social Prescriptions Mainstream. Journal of the Royal Society of Medicine. 2020;113(1):4.

Exclusion reason: Study design

1. Gottlieb L, Cottrell EK, Park B, Clark KD, Gold R, Fichtenberg C. Advancing Social Prescribing with Implementation Science. Journal of the American Board of Family Medicine. 2018;31(3):315-21.

Exclusion reason: Study design

1. Green BB, Anderson ML, Wang CY, Vernon SW, Chubak J, Meenan RT, Fuller S. Results Of Nurse Navigator Follow-Up After Positive Colorectal Cancer Screening Test: A Randomized Trial. Journal of the American Board of Family Medicine. 2014;27(6):789-95.

Exclusion reason: Population

1. Guo Y, Vogel WB, Muller KE, Stoner D, Huo T, Shenkman EA. The Wellness Incentive And Navigation Intervention Improved Health-Related Quality Of Life Among Medicaid Enrollees: A Randomized Pragmatic Clinical Trial. Health Services Research. 2019;54(6):1156‐65.

Exclusion reason: Population

1. Halkitis PN, Kupprat SA, Mukherjee PP. Longitudinal associations between case management and supportive services use among Black and Latina HIV- positive women in New York City. Journal of Women’s Health. 2010;19(1):99–108.

Exclusion reason: Population

1. Hamilton-West KE, Gadsby E, Hotham S. Improving The Evidence Base For Social Prescribing. BMJ. 2019;364.

Exclusion reason: Intervention

1. Happell B, Stanton R, Scott D. Utilization Of A Cardiometabolic Health Nurse - A Novel Strategy To Manage Comorbid Physical And Mental Illness. Journal of Multimorbidity and Comorbidity. 2014;4:22-8.

Exclusion reason: Intervention

1. Heatwole Shank KS, Kenley B, Brown S, Shipley J, Baum M, Beers C. "We Need More Things For Us": Being Low Income And Under Occupied In Older Age. Canadian Journal of Occupational Therapy. 2020;87(1):21-9.

Exclusion reason: Study design

1. Hendren S, Chin N, Fisher S, Winters P, Griggs J, Mohile S, Fiscella K. Patients’ barriers to receipt of cancer care, and factors associated with needing more assistance from a patient navigator. Journal of the National Medical Association. 2011;103(8):701–10.

Exclusion reason: Population

1. Highfield L, Ottenweller C, Pfanz A, Hanks J. Interactive Web-Based Portals To Improve Patient Navigation and Connect Patients With Primary Care And Specialty Services In Underserved Communities. Perspective in Health Information Management. 2014;11:1.

Exclusion reason: Study design

1. Hindle L. A New Framework For Social Prescribing For AHPs. Podiatry Now. 2019;22(9):6-.

Exclusion reason: Study design

1. Holtrop JS, Luo Z, Piatt G, Green LA, Chen Q, Piette J. Diabetic and Obese Patient Clinical Outcomes Improve During a Care Management Implementation in Primary Care. Journal of Primary Care & Community Health. 2017;8(4):312-8.

Exclusion reason: Population

1. Honeycutt S, Green R, Ballard D, Hermstad A, Brueder A, Haardorfer R, Yam J, Arriola KJ. Evaluation Of A Patient Navigation Program To Promote Colorectal Cancer Screening In Rural Georgia, USA. Cancer. 2013;119(16):3059-66.

Exclusion reason: Intervention

1. Horne HN, Phelan-Emrick DF, Pollack CE, Markakis D, Wenzel J, Ahmed S, Garza MA, Shapiro GR, Bone LR, Johnson LB, Ford JG. Effect Of Patient Navigation On Colorectal Cancer Screening In A Community-Based Randomized Controlled Trial Of Urban African American Adults. Cancer Causes & Control. 2015;26(2):239-46.

Exclusion reason: Intervention

1. Howarth M, Griffiths A, da Silva A, Green R. Social Prescribing: A 'Natural' Community-Based Solution. British Journal of Community Nursing. 2020;25(6):294-8.

Exclusion reason: Study design

1. Howarth M, Leigh J. Social Prescribing: Collaboration In Times Of Stability And Crisis. British Journal of Nursing. 2020;29(10):578-9.

Exclusion reason: Study design

1. Hoy-Rosas J, Kaur M, Mayer VL, Edelman L, Arend J. Partnership Between Providers And Community-Based Health Coaches To Improve Diabetes Outcomes In Primary Care. Journal of General Internal Medicine. 2017;32(2 Suppl. 1): S781.

Exclusion reason: Population

1. Huddy J. A New Hope: Social Prescribing in Cornwall. The British Journal of General Practice. 2019;69(682):243.

Exclusion reason: Study design

1. Hudon C, Chouinard M-C, Dubois M-F, Roberge P, Loignon C, Tchouke, Lambert M, Hudon E, Diadiou F, Bouliane D. Case Management in Primary Care for Frequent Users of Health Care Services: A Mixed Methods Study. Annals of Family Medicine. 2018;16(3):232-9.

Exclusion reason: Intervention

1. Husk K, Blockley K, Lovell R, Bethel A, Lang I, Byng R, Garside, R. What Approaches To Social Prescribing Work, For Whom, And In What Circumstances? A Realistic Review. Health & Social Care in the Community. 2020;28(2):309-24.

Exclusion reason: Study design

1. Husk K, Elston J, Gradinger F, Callaghan L, Asthana S. Social Prescribing: Where Is The Evidence? British Journal of General Practice. 2019;69(678):6-7.

Exclusion reason: Study design

1. Iglesias K, Baggio S, Moschetti K, Wasserfallen J-B, Hugli O, Daeppen J-B, Burnand B, Bodenmann P. Using Case Management In A Universal Health Coverage System To Improve Quality Of Life Of Frequent Emergency Department Users: A Randomized Controlled Trial. Quality of Life Research. 2018;27(2):503-13.

Exclusion reason: Wrong setting

1. Iglesias K, Moschetti K, Baggio S, Velonaki VS, Ruggeri O, Hugli O, Burnand B, Wasserfallen J, Daeppen J, Bodenmann P. Impact of case management on frequent users' quality of life: A randomized controlled trial. Journal of General Internal Medicine. 2015;30(Suppl. 2): S59-S60.

Exclusion reason: Intervention

1. Iliffe S, Waugh A, Poole M, Bamford C, Brittain K, Chew-Graham C, Fox C, Katona C, Livingston G, Manthorpe J, Steen N, Stephens B, Hogan V, Robinson L. The Effectiveness Of Collaborative Care For People With Memory Problems In Primary Care: Results Of The Caredem Case Management Modelling And Feasibility Study. Health Technology Assessment. 2014;18(52):1-148.

Exclusion reason: Population

1. Isrctn. Case management to enhance occupational support (CAMEOS). 2014.

Exclusion reason: Intervention

1. Jan C-F, Chiu T-Y, Chen C-Y, Guo F-R, Lee M-C. A 10-Year Review Of Health Care Reform On Family Practice Integrated Care Project-Taiwan Experience. Family Practice. 2018;35(4):352-7.

Exclusion reason: Study design

1. Jandorf L, Braschi C, Ernstoff E, Wong CR, Thelemaque L, Winkel G, Redd W, Itzkowitz SH. Culturally Targeted Patient Navigation For Increasing African Americans' Adherence To Screening Colonoscopy: A Randomized Clinical Trial. Cancer. 2013;22(9):1577-87.

Exclusion reason: Intervention

1. Jandorf L, Stossel LM, Cooperman JL, Graff Zivin J, Ladabaum U, Hall D, Thelemaque LD, Itzkowitz SH. Cost Analysis of a Patient Navigation System to Increase Screening Colonoscopy Adherence Among Urban Minorities. Cancer. 2013;119(3):612-20.

Exclusion reason: Intervention

1. Jani A, Bertotti M, Lazzari A, Drinkwater C, Addarii F, Conibear J, Gray M. Investing Resources To Address Social Factors Affecting Health: The Essential Role Of Social Prescribing. Journal of the Royal Society of Medicine. 2020;113(1):24-7.

Exclusion reason: Study design

1. Jani A, Gray M. Making Social Prescriptions Mainstream. Journal of the Royal Society of Medicine. 2019;112(11):459-61.

Exclusion reason: Study design

1. Johnson JD, Pollard M. Community-Based Care Coordination To Overcome Fragmentation Of Services In Aurora, Colorado. Journal of General Internal Medicine. 2013;28(Suppl. 1): S430-S1.

Exclusion reason: Study design

1. Johnson SL, Gunn VL. Community Health Workers as a Component of the HealthCare Team. Pediatric Clinics of North America. 2015;62(5):1313-28.

Exclusion reason: Study design

1. Joo JY. Community-Based Case Management and Outcomes In Medicare Beneficiaries. Iowa Research. 2013.

Exclusion reason: Intervention

1. Karliner LS, Kobashi B, Kuryan C, Lam R, Fox RK. Enhancing Hepatitis C and HIV Screening and Linkage to Treatment in Primary Care Practice. Journal of General Internal Medicine. 2017;32(2 Suppl. 1): S748.

Exclusion reason: Intervention

1. Kelley T. Population Care Coordinators: A Key To Improved Care At Lower Cost? Managed care. 2014;23(9):46-50.

Exclusion reason: Study design

1. Kelly E, Duan L, Cohen H, Kiger H, Pancake L, Brekke J. Integrating Behavioral Healthcare For Individuals With Serious Mental Illness: A Randomized Controlled Trial Of A Peer Health Navigator Intervention. Schizophrenia Research. 2017; 182:135-41.

Exclusion reason: Population

1. Kelly E, Fulginiti A, Pahwa R, Tallen L, Duan L, Brekke JS. A Pilot Test Of A Peer Navigator Intervention For Improving The Health Of Individuals With Serious Mental Illness. Community Mental Health Journal. 2014;50(4):435-46.

Exclusion reason: Population

1. Kelly EL, Braslow JT, Brekke JS. Using Electronic Health Records to Enhance a Peer Health Navigator Intervention: a Randomized Pilot Test for Individuals with Serious Mental Illness and Housing Instability. Community Mental Health Journal. 2018;54(8):1172‐9.

Exclusion reason: Population

1. Kennedy M. Community-Based Health Plans Take the (Complex) Path to Integrated Care. American Society on Aging. 2013;37(2):30-2.

Exclusion reason: Study design

1. Kenning C, Lovell K, Hann M, Agius R, Bee PE, Chew-Graham C, Coventry P, Van Der Feltz-Cornelis CM, Gilbody S, Hardy G, Kellett S, Kessler D, McMillan D, Reeves D, Rick J, Sutton M, Bower P. Collaborating Case Management to Aid Return to Work After Long-Term Sickness Absence: A Pilot Randomised Controlled Trial. Public Health Research. 2018.

Exclusion reason: Population

1. Kenyon S, Jolly K, Hemming K, Hope L, Blissett J, Dann SA, Lilford R, MacArthur C. Lay Support For Pregnant Women with Social Risk: A Randomised Controlled Trial. BMJ Open. 2016;6(3): e009203.

Exclusion reason: Population

1. Kern LM, Edwards AM, Dhopeshwarkar RV, Kaushal R. Association Between the Patient-Centered Medical Home and Quality of Care. Journal of General Internal Medicine. 2013;28(Suppl. 1): S26-S7.

Exclusion reason: Intervention

1. Kiely B, Clyne B, Boland F, O’Donnell P, Connolly D, O’Shea E, Smith SM. Use Of Link Workers To Provide Social Prescribing And Health And Social Care Coordination For People With Complex Multimorbidity In Socially Deprived Areas. BMJ Open. 2019;11(2):1-9.

Exclusion reason: Study design

1. Kilgarriff-Foster A, O'Cathain A. Exploring the Components and Impact of Social Prescribing. Journal of Public Mental Health. 2015;14(3):127-34.

Exclusion reason: Study design

1. Kim SE, Michalopoulos C, Kwong RM, Warren A, Manno MS. Telephone Care Management's Effectiveness In Coordinating Care For Medicaid Beneficiaries In Managed Care: A Randomized Controlled Study. Health Services Research. 2013;48(5):1730-49.

Exclusion reason: Population

1. Kim TY, Mortensen K, Eldridge B. Linking Uninsured Patients Treated In The Emergency Department To Primary Care Shows Some Promise In Maryland. Health Affairs. 2015;34(5):796-804.

Exclusion reason: Intervention

1. Kinchin I, Jacups S, Mann J, Quigley R, Harvey D, Doran CM, Strivens E. Efficacy and Cost-Effectiveness of a Community-Based Model Of Care For Older Patients with Complex Needs: A Study Protocol For A Multicentre Randomised Controlled Trial Using A Stepped Wedge Cluster Design. Trials. 2018;19(1):668.

Exclusion reason: Population

1. King AII, Boyd ML, Raphael DL, Jull A. The Effect Of A Gerontology Nurse Specialist For High Needs Older People in the Community On Healthcare Utilisation: A Controlled Before-After Study. BMC Geriatrics. 2018;18(1):22.

Exclusion reason: Intervention

1. King MA, Sav A, McSwan J. Abstracts of Papers Presented at the Health Services Research & Pharmacy Practice Conference, 16-17 April 2015, Riddel Hall, Queen's University Belfast. International Journal of Pharmacy Practice. 2015;23 Suppl 1:2-48.

Exclusion reason: Study design

1. Kleinpell Ruth M, Ely E Wesley, Grabenkort Robert W. Shock LP. Care Coordination and Care Team Redesign. Advance for NPs &PAs. 2013;4(4):13.

Exclusion reason: Study design

1. Kramer A, Nosbusch J, Rice J. Safe mom, safe baby: A collaborative model of care for pregnant women experiencing intimate partner violence. Journal of Perinatal & Neonatal Nursing. 2012;26(4):307–18.

Exclusion reason: Study design

1. Krantz MJ, Coronel SM, Whitley EM, Dale R, Yost J, Estacio RO. Effectiveness Of A Community Health Worker Cardiovascular Risk Reduction Program In Public Health And Health Care Settings. American Public Health Association. 2013;103(1): e19-27.

Exclusion reason: Wrong outcomes

1. Labrada M, Mintzer MJ, Karanam C, Castellanos R, Cruz L, Hoang M, Wieger R, Aguilar E, Florez H, Ruiz JG. Dramatic Reduction in 30-Day Readmissions Through High-Risk Screening and Two-Phase Interdisciplinary Care. Southern Medical Journal. 2017;110(12):757-60.

Exclusion reason: Population

1. Lairson DR, Dicarlo M, Deshmukh AA, Fagan HB, Sifri R, Katurakes N, Cocroft J, Sendecki J, Swan H, Vernon SW, Myers RE. Cost-Effectiveness Of A Standard Intervention Versus A Navigated Intervention On Colorectal Cancer Screening Use In Primary Care. Cancer. 2014;120(7):1042-9.

Exclusion reason: Intervention

1. Lalonde L, Goudreau J, Hudon E, Lussier MT, Bareil C, Duhamel F, et al. Development of An Interprofessional Program for Cardiovascular Prevention in Primary Care: A Participatory Research Approach. SAGE Open Medicine. 2014;2(101624744):2050312114522788.

Exclusion reason: Intervention

1. Larkey L, Szalacha L, Herman P, Gonzalez J, Menon U. Randomized Controlled Dissemination Study of Community-To-Clinic Navigation To Promote CRC Screening: Study Design and Implications. Contemporary Clinical Trials. 2017; 53:106-14.

Exclusion reason: Wrong outcomes

1. Larsen IG, Oestergaard LG, Thomsen LM, Nielsen CV, Schiottz-Christensen B. Effect Of Adding Lay-Tutors To A Back To School Programme For Patients With Subacute, Non-Specific Low Pain: A Randomized Controlled Clinical Trial With A Two-Year Follow-Up. Journal of Rehabilitation Medicine.2019;51(9):698‐704.

Exclusion reason: Population

1. Lasser KE, Buitron de la Vega P, Ashe EM, Xuan Z, Alva S, Battisti L, Losi S, Sieber C, Richards C, Sullivan P, Triscari L, Brody L, Roth M, LeBlanc A, Silverstein M. A Pharmacy Liaison-Patient Navigation Intervention To Reduce Inpatient And Emergency Department Utilization Among Primary Care Patients In A Medicaid Accountable Care Organization: A Pragmatic Trial Protocol. Contemporary Clinical Trials. 2020; 94:106046.

Exclusion reason: Study design

1. Lasser KE, Kenst KS, Quintiliani LM, Wiener RS, Murillo J, Pbert L, Xuan Z, Bowen DJ. Patient Navigation To Promote Smoking Cessation Among Low-Income Primary Care Patients: A Pilot Randomized Controlled Trial. Journal of Ethnicity in Substance Abuse. 2013;12(4):374-90.

Exclusion reason: Intervention

1. Lasser KE, Quintiliani LM, Truong V, Xuan Z, Pbert L, Xuan Z, Bowen DJ. Patient Navigation To Promote Smoking Cessation In Primary Care: Preliminary Findings From An Ongoing Randomized Controlled Trial. Journal of General Internal Medicine. 2017;32(2 Suppl. 1): S266.

Exclusion reason: Intervention

1. Layne LR K, Longnecker J, Holmes RM. Integrating patients into a healthcare home through a patient navigation program. 2012.

Exclusion reason: Study design

1. Lee JH, Fulp W, Wells KJ, Meade CD, Calcano E, Roetzheim R. Patient Navigation And Time To Diagnostic Resolution: Results For A Cluster Randomized Trial Evaluating The Efficacy Of Patient Navigation Among Patients With Breast Cancer Screening Abnormalities, Tampa, Fl. Plos One. 2013;8(9):74542.

Exclusion reason: Population

1. Lee JJ, Bae SG. Implementation Of A Care Coordination System For Chronic Diseases. Yeungnam University Journal Of Medicine. 2019;36(1):1-7.

Exclusion reason: Study design

1. Lee N, Kwak M, Jeong M, Choi EJ, Lim E, Kwon IB, Lee W, Ku H, Kim D, Nam H, Na J, Park M. Pilot Testing Of An Ict-Based Care Management Support System To Deliver Integrated Community Care. Studies In Health Technology And Informatics. 2019; 264:1821-2.

Exclusion reason: Study design

1. Lemke M, Kappel R, McCarter R, D'Angelo L, Tuchman LK. Perceptions Of Health Care Transition Care Coordination In Patients With Chronic Illness. Pediatrics. 2018;141(5).

Exclusion reason: Intervention

1. Lennane S. Social Prescribing In The GP Surgery. British Journal of Healthcare Assistants. 2019;13(4):176-7.

Exclusion reason: Study design

1. Li Y, Carlson E, Villarreal R, Meraz L, Pagan JA. Cost-Effectiveness Of a Patient Navigation Program To Improve Cervical Cancer Screening. The American Journal of Managed Care. 2017;23(7):429-34.

Exclusion reason: Intervention

1. Lim KY, Son SJ, Hong CH. Effect of Multidisciplinary Management in Community Dwelling Patients with Depression: Preliminary Study. 2018;48: S447.

Exclusion reason: Population

1. Ling SM, McGann P. Changing Healthcare Service Delivery to Improve Health Outcomes For Older Adults: Opportunities Not to Be Missed. Journal of the American Geriatrics Society. 2018;66(2):235-8.

Exclusion reason: Study design

1. Linkins K, Brya J, Oelschlaeger A, Simonson B, Lahiri S, McFeeters J, Schutze M, Jonas J, Mowry M. Influencing the disability trajectory for workers with serious mental illness: Lessons from Minnesota’s Demonstration to Maintain Independence and Employment. Journal of Vocational Rehabilitation. 2011; 34(2):107–18.

Exclusion reason: Population

1. Looman W, Bleijenberg N, Karimi M, Hoedemakers M, De Wit N, Rutten-van Mölken M. Multi-Criteria Decision Analysis of a Proactive Person-Centred Integrated Primary Care Program Care for Frail Elderly in the Netherlands: U-PROFIT. International Journal of Integrated Care. 2019;19(4):1-2.

Exclusion reason: Intervention

1. Lopez Vallejo M, Puente Alcaraz J. Institutionalisation of the Case Management Nurse in Spain. Comparative Analysis of Health Systems of the Spanish Autonomous Communities. Enfermeria Clinica. 2019;29(2):107-18.

Exclusion reason: Study design

1. Lynch M, Jones C. Social Prescribing for Frequent Attenders: Findings from an Innovative Pilot Intervention. The Lancet. 2019;394(Suppl. 2): S69.

Exclusion reason: Study design

1. Mabunda SA, London L, Pienaar D. An Evaluation of the Role of an Intermediate Care Facility in the Continuum of Care in Western Cape, South Africa. International Journal of Health Policy and Management. 2018;7(2):167-79.

Exclusion reason: Study design

1. MacRi J, Garcia ME, Rapp H, Aoki M. Improving Engagement in Depression Treatment at an Academic Medical Center through a Telephone-Based Depression Navigator Program. Journal of General Internal Medicine. 2019;34(Suppl. 3): S739.

Exclusion reason: Population

1. Maeng D, Davis D, Tomcavage J, Graf T, Procopio K. Improving patient experience by transforming primary care: evidence from geisinger’s patient-centered medical homes. Population Health Management. 2013;16(3):157–63.

Exclusion reason: Study design

1. Marcotte LM, Reddy A, Zhou L, Miller SC, Hudelson C, Liao JM. Trends in Utilization of Transitional Care Management in the United States. JAMA Network Open. 2020;3(1):1-3.

Exclusion reason: Study design

1. Marek KD, Stetzer F, Adams SJ, Bub LD, Schlidt A, Colorafi KJ. Cost Analysis of a Home-Based Nurse Care Coordination Program. Geriatrics Healthcare Professionals. 2014;62(12):2369-76.

Exclusion reason: Intervention

1. Marek KD, Stetzer F, Ryan PA, Bub LD, Adams SJ, Schlidt A, Lancaster R, O’Brien A. Nurse Care Coordination and Technology Effects on Health Status of Frail Older Adults Via Enhanced Self-Management of Medication: Randomized Clinical Trial To Test Efficacy. Nursing Research. 2013;62(4):269-78.

Exclusion reason: Intervention

1. Marshall JK, Mbah OM, Ford JG, Phelan-Emrick D, Ahmed S, Bone L, Wenzel J, Shapiro GR, Howerton M, Johnson L, Brown Q, Ewing A, Pollack CE. Effect of Patient Navigation on Breast Cancer Screening Among African American Medicare Beneficiaries: A Randomized Controlled Trial. Journal of General Internal Medicine. 2016;31(1):68-76.

Exclusion reason: Intervention

1. Martin RL, Tully M, Kos A, Frazer D, Williamson A, Conlon A, Enser JJ, LoConte NK. Increasing Colorectal Cancer Screening at an Urban FQHC Using iFOBT and Patient Navigation. Cancer Research & Prevention. 2017;18(5):741-50.

Exclusion reason: Intervention

1. Mas-Expósito L, Amador-Campos JA, Gómez-Benito J, Lalucat-Jo L. Depicting Current Case Management Models. Journal of Social Work. 2013;14(2):133-46.

Exclusion reason: Study design

1. Masson CL, Delucchi KL, McKnight C, Hettema J, Khalili M, Min A, et al. A Randomized Trial of a Hepatitis Care Coordination Model in Methadone Maintenance Treatment. American Public Health Association. 2013;103(10):81-8.

Exclusion reason: Intervention

1. Mateo-Abad M, Gonzalez N, Fullaondo A, Merino M, Azkargorta L, Gine A, Verdoy D, Vergara I, Keenoy E. Impact of the CareWell Integrated Care Model for Older Patients with Multimorbidity: A Quasi-Experimental Controlled Study in the Basque Country. BMC Health Services Research. 2020;20(1):613.

Exclusion reason: Intervention

1. Matousek AC, Addington SR, Kahan J, Shannon H, Luckner T, Exe C, Eisenhower RR, Louis J, Lipsitz S, Meara JG, Riviello R. Patient Navigation by Community Health Workers Increases Access to Surgical Care in Rural Haiti. World Journal of Surgery. 2017;41(12):3025-30.

Exclusion reason: Wrong setting

1. Mattei da Silva AT, de Fatima Mantovani M, Castanho Moreira R, Perez Arthur J, Molina de Souza R. Nursing Case Management for People with Hypertension in Primary Health Care: A Randomized Controlled Trial. Research in Nursing & Health. 2020;43(1):68-78.

Exclusion reason: Intervention

1. Mbah O, Ford JG, Qiu M, Wenzel J, Bone L, Bowie J, Elmi A, Slade JL, Towson M, Dobs AS. Mobilizing Social Support Networks to Improve Cancer Screening: The COACH Randomized Controlled Trial Study Design. BMC Cancer. 2015;15(1):907.

Exclusion reason: Study design

1. McAlister FA, Grover S, Padwal RS, Youngson E, Fradette M, Thompson A, Buck B, Dean N, Tsuyuki RT, Shuaib A, Majumdar SR. Case Management Reduces Global Vascular Risk After Stroke: Secondary Results from the Preventing Recurrent Vascular Events and Neurological Worsening through Intensive Organized Case-Management Randomized Controlled Trial. American Heart Journal. 2014;168(6):924-30.

Exclusion reason: Population

1. McBrien KA, Ivers N, Barnieh L, Bailey JJ, Lorenzetti DL, Nicholas D, Tonelli M, Hemmelgarn B, Lewanczuk R, Edwards A, Braun T, Manns B. Patient navigators for People with Chronic Disease: A Systematic Review. Plos One. 2018;13(2).

Exclusion reason: Study design

1. McCann TV, Clark E. Adopting care provider-facilitator roles: community mental health nurses and young adults with an early episode of schizophrenia. Social Theory & Health. 2005;3(1):39–60.

Exclusion reason: Study design

1. McCloskey J. Promotores as partners in a community-based diabetes intervention program targeting Hispanics. Family & Community Health. 2009;32(1):48–57.

Exclusion reason: Population

1. McGregor LM, Kerrison RS, Green T, Macleod U, Hughes M, Gibbins M, et al. Using Primary Care-Based Paper and Telephone Interventions to Increase Uptake of Bowel Scope Screening in Yorkshire: A Protocol of a Randomised Controlled Trial. BMJ Open. 2018;8(7):1-8.

Exclusion reason: Intervention

1. McLoughlin P, Murphy E, O'Sullivan F, Connellan C. An Integrated Care Approach to the Uses of Social Prescribing an Acutely Frail Older Adult Cohort. Age and Ageing. 2019;48(Suppl.3).

Exclusion reason: Study design

1. Menon U, Szalacha LA, Kue J, Herman PM, Bucho-Gonzalez J, Lance P, Larkey L. Effects of a Community-to-Clinic Navigation Intervention on Colorectal Cancer Screening Among Underserved People. Annals of Behavioral Medicine. 2020;54(5):308-19.

Exclusion reason: Intervention

1. Messmer E, Brochier A, Joseph M, Tripodis Y, Garg A. Impact of an On-Site Versus Remote Patient Navigator on Pediatricians' Referrals and Families' Receipt of Resources for Unmet Social Needs. Journal of Primary Care & Community Health. 2020; 1:1-6

Exclusion reason: Population

1. Miyamoto RES, Hermosura AH, Acido DAM. A Culture-Based Family-Centered Health Navigation Intervention for Chronic Disease Management in Native Hawaiians. Hawai'i Journal of Medicine & Public Health.2019;78(6):78-82.

Exclusion reason: Study design

1. Molina Y, Glasgow AE, Kim SJ, Watson KS, Darnell JS, Calhoun EA. Patient Navigation in Medically Underserved Areas study design: A trial with implications for efficacy, effect modification, and full continuum assessment. Contemporary Clinical Trials. 2017;53:29-35.

Exclusion reason: Intervention

1. Molina Y, Kim SJ, Berrios N, Glasgow AE, San Miguel Y, Darnell JS, Pauls H, Vijayasiri G, Warnecke RB, Calhoun EA. Patient Navigation Improves Subsequent Breast Cancer Screening after a Noncancerous Result: Evidence from the Patient Navigation in Medically Underserved Areas Study. Journal of Women’s Health. 2018;27(3):317‐23.

Exclusion reason: Intervention

1. Moller UO, Kristensson J, Midlov P, Ekdahl C, Jakobsson U. Effects of a One-Year Home-Based Case Management Intervention on Falls in Older People: A Randomized Controlled Trial. Journal of Aging and Physical Activity. 2014;22(4):457-64.

Exclusion reason: Intervention

1. Montgomery P, Jermyn D, Bailey P, Nangia P, Egan M, Mossey S. Community Reintegration of Stroke Survivors: The Effect of a Community Navigation Intervention. Journal of Advanced Nursing. 2015;71(1):214-25.

Exclusion reason: Study design

1. Morton L, Ferguson M, Baty F. Improving Wellbeing and Self-Efficacy by Social Prescription. Public Health. 2015;129(3):286-9.

Exclusion reason: Wrong setting

1. Moschetti K, Iglesias K, Baggio S, Velonaki VS, Ruggeri O, Hugli O, Burnand B, Wasserfallen JB, Iglesias K, Baggio S, Daeppen JB. Potential Health-Care Service Utilization Substitution Effects Induced by Case Management for Emergency Department Frequent Users. Journal of General Internal Medicine. 2015;(3).

Exclusion reason: Wrong setting

1. Muliira JK, D'Souza MS. Effectiveness of Patient Navigator Interventions on Uptake of Colorectal Cancer Screening in Primary Care Settings. Japan Journal of Nursing Science. 2016;13(2):205-19.

Exclusion reason: Study design

1. Mulligan K, Bhatti S, Rayner J, Hsiung S. Social Prescribing: Creating Pathways Towards Better Health and Wellness. Journal of the American Geriatrics Society. 2020;68(2):426-8.

Exclusion reason: Study design

1. Mullins CD, Shaya FT, Blatt L, Saunders E. A qualitative evaluation of a citywide Community Health Partnership program. Journal of the National Medical Association. 2012;104(1-2):53–60.

Exclusion reason: Study design

1. Myers G. Care Coordination Closes Gaps in Care for High-Risk Seniors. Case in Point. 2014;12(4):23.

Exclusion reason: Study design

1. Myers RE, Bittner-Fagan H, Daskalakis C, Sifri R, Vernon SW, Cocroft J, Dicarlo D, Katurakes N, Andrel J. A Randomized Controlled Trial of a Tailored Navigation and a Standard Intervention in Colorectal Cancer Screening. Cancer Epidemiology, Biomarkers & Prevention. 2013;22(1):109-17.

Exclusion reason: Intervention

1. Myers RE, Sifri R, Daskalakis C, DiCarlo M, Geethakumari PR, Cocroft J, Minnick C, Brisbon N, Vernon SW. Increasing Colon Cancer Screening in Primary Care among African Americans. Journal of the National Cancer Institute. 2014;106(12);1-7.

Exclusion reason: Intervention

1. Natale-Pereira A, Enard KR, Nevarez L, Jones LA. The role of patient navigators in eliminating health disparities. Cancer 2011, 117(SUPPL. 15): 3543-52.

Exclusion reason: Study design

1. Nct. Addressing Social Determinants of Health & Diabetes Self-Management in Vulnerable Populations. 2019.

Exclusion reason: Study design

1. Nct. Apoyo Con Cariño: Patient Navigation for Palliative Care for Non-Cancer Illness. 2017.

Exclusion reason: Study design

1. Nct. Comparing Interventions to Increase Colorectal Cancer Screening. 2017.

Exclusion reason: Study design

1. Nct. Enhancing Community Health Through Patient Navigation, Advocacy and Social Support. 2017.

Exclusion reason: Population

1. Nct. Enhancing Prevention Pathways Toward Tribal Colorectal Health. 2016.

Exclusion reason: Study design

1. Nct. Guidelines to Practice: Reducing Asthma Health Disparities Through Guideline Implementation. 2014.

Exclusion reason: Intervention

1. Nct. Improving Comprehensive Cancer Screening Among Vulnerable Patients Using Patient Navigation. 2015.

Exclusion reason: Intervention

1. Nct. Integrated Care Delivery of HIV Prevention and Treatment in AGYW in Zambia. 2019.

Exclusion reason: Population

1. Nct. Navigation From Community to Clinic to Promote CRC Screening in Underserved Populations. 2013.

Exclusion reason: Intervention

1. Nct. Patient-Centered Cancer Prevention In Chinese Americans. 2017.

Exclusion reason: Study design

1. Nct. Patient Navigation and Financial Incentives to Promote Smoking Cessation. 2015.

Exclusion reason: Population

1. Nct. Patient Navigation for Lung Screening at MGH Community Health Centers. 2016.

Exclusion reason: Intervention

1. Nct. Patient Navigation for Medicaid Frequent ED Users. 2013.

Exclusion reason: Population

1. Nct. Patient Navigation in Primary Care and Access to Resources in the Community. 2018.

Exclusion reason: Study design

1. Nct. PATient Navigator to rEduce Readmissions. 2014.

Exclusion reason: Population

1. Nct. Patients Navigators in Facilitating Weight Management in Obese Participants. 2018.

Exclusion reason: Study design

1. Nct. Peer Navigators to Address Obesity-Related Concerns for African Americans With Serious Mental Illness. 2017.

Exclusion reason: Population

1. Nct. Pregnancy and Chronic Disease: the Effect of a Midwife-coordinated Maternity Care Intervention. 2018.

Exclusion reason: Population

1. Nct. Promoting Cancer Screening Among Medicaid Recipients in Minnesota. 2017.

Exclusion reason: Intervention

1. Nct. Redes II National Patient Navigator Intervention Study. 2014.

Exclusion reason: Population

1. Nct. Rural Interventions for Screening Effectiveness. 2016.

Exclusion reason: Study design

1. Nct. Social Prescribing Improving Mental Health Study. 2019.

Exclusion reason: Study design

1. Nct. Study on the Impact of Patient Navigators on the Health Education and Quality of Life in Formerly Incarcerated Patients. 2014.

Exclusion reason: Study design

1. Nct. The OPTIMIZE Study: Optimizing Patient Navigation for Perinatal Care. 2020.

Exclusion reason: Study design

1. Nct. Using Lay Health Advisors to Improve Hypertension Management. 2018.

Exclusion reason: Study design

1. Nembhard IM, Buta E, Lee YSH, Anderson D, Zlateva I, Cleary PD. A Quasi-Experiment Assessing the Six-Months Effects of a Nurse Care Coordination Program on Patient Care Experiences and Clinician Teamwork in Community Health Centers. BMC Health Services Research. 2020;20(1):137.

Exclusion reason: Intervention

1. No author. Case management: a success story. Professional Case Management. 2013;18(1):3-4.

Exclusion reason: Study design

1. No author. Community-based health coaches and care coordinators reduce readmissions using Information Technology. Remington Report. 2015;23(2):15-6.

Exclusion reason: Study design

1. No author. Community Finds Unique Ways to Bring Case Management to the Frontier: ED Use Decreased, Care Coordination Improved. Case Management Advisor. 2018;29(1).

Exclusion reason: Study design

1. No author. Guided care provides better quality of care for chronically ill older adults: patients also use less home care services. Home Healthcare Nurse. 2013;31(2):115-.

Exclusion reason: Intervention

1. No author. Health System's Integrated Care Program Is a People-Centered Strategy. Hospital Case Management. 2019;27(6):72.

Exclusion reason: Study design

1. No author. Integrated Case Management Model Shows Which Resources Are Needed: Process includes mental health. Case Management Advisor. 2018;29(5):55-6.

Exclusion reason: Study design

1. No author. International Navigation: The Challenges and Successes of Patient Navigation in Guatemala. Journal of Oncology Navigation & Survivorship. 2020;11(4):125-6.

Exclusion reason: Study design

1. No author. Recipe for success: Study identifies cost-saving practices for complex patient care. Case Management Monthly. 2014;11(11):1-5.

Exclusion reason: Study design

1. No author. The Benefits of Using Care Coordinators in Primary Care: A Case Study. American Academy of Family Physicians; 2013. p. 18-21.

Exclusion reason: Study design

1. No author. Work with Community Partners to Develop Solutions for Opioid Use Disorder Patients: Medication-Assisted Treatment, Peer Navigators, and Sophisticated Tracking Systems are Important Tools. ED Management. 2019;31(8).

Exclusion reason: Population

1. Norton WE, Larson RS, Dearing JW. Primary Care and Public Health Partnerships for Implementing Pre-Exposure prophylaxis. American Journal of Preventive Medicine. 2013;44(2): S77-9.

Exclusion reason: Study design

1. Novak KL, Kathol B, Swain MG, Johnston C, Kwan J, Bucholtz L. Nurse-led, Interactive Group Sessions for Dyspepsia and Gerd Referrals: A Prospective Controlled Study of the "Nurse Navigator" Clinic. Gastroenterology. 2014;146(5): S192-S3.

Exclusion reason: Population

1. Ntr. E-consultation between primary and secondary caregivers to arrange health care services more efficiently. 2014.

Exclusion reason: Study design

1. Ntr. The effectiveness of Family Group Conferencing in The Netherlands, Implementation and Effectiveness: The Role of Family Managers, Method Characteristics and Family Factors. 2013.

Exclusion reason: Population

1. Nundy S, Dick JJ, Hogan P, Goddu AP, Bussie A, Chin M,. Using Text Messaging to Provide Self-Management Support and Care Coordination for Individuals with Chronic Disease. Journal of General Internal Medicine. 2013;28; S449-50.

Exclusion reason: Population

1. Nunn S. Psychological Well-Being, Physical Health and Social Prescribing in the Context of Social Research. Thorax. 2020;75(7):536.

Exclusion reason: Study design

1. Nurjono M, Shrestha P, Ang IYH, Shiraz F, Eh KX, Ee S, Toh S, Vrijhoef HJM. Shifting Care from Hospital to Community, A Strategy to Integrate Care in Singapore: A Process Evaluation of Implementation Fidelity. BMC Health Services Research. 2020;20(1):452.

Exclusion reason: Study design

1. Nwokeji ED, Bohman TM, Wallisch L, Stoner D, Christensen K, Spence RR, Reed BC, Ostermeyer B. Evaluating Patient Adherence to Antidepressant Therapy Among Uninsured Working Adults Diagnosed with Major Depression: Results of the Texas Demonstration to Maintain Independence and Employment Study. Administration and Policy in Mental Health and Mental Health Services Research. 2012;39(5):374-82.

Exclusion reason: Population

1. Nyatanga B. Social prescribing: Combating Loneliness is Everyone's Business. British Journal of Community Nursing. 2020;25(4):200.

Exclusion reason: Study design

1. Odusanya R, Lindahl J, Martinez J, Uwemedimo OT, Jardine S, Kim EJ. Utilization of Patient Navigators: An Innovative Method for Connecting Patients with Community Resources Within the Social Health Alliance to Promote Health Equity (S.H.A.P.E) Program. Journal of General Internal Medicine. 2019;34(2):774.

Exclusion reason: Study design

1. Palinkas LA, Ell K, Hansen M, Cabassa L, Wells A. Sustainability of collaborative care interventions in primary care settings. Journal of Social Work. 2011;11(1):99–117.

Exclusion reason: Study design

1. Parry W, Wolters AT, Brine RJ, Steventon A. Effect of an Integrated Care Pathway on Use of Primary and Secondary Healthcare By Patients at High Risk of Emergency Inpatient Admission: A Matched Control Cohort Study in Tower Hamlets. BMJ Open. 2019;9(6);1-16.

Exclusion reason: Study design

1. Peart A, Lewis V, Brown T, Russell G. Patient Navigators Facilitating Access to Primary Care: A Scoping Review. BMJ Open. 2018;8(3).

Exclusion reason: Study design

1. Percac-Lima S, Ashburner JM, McCarthy AM, Piawah S, Atlas SJ. Patient Navigation to Improve Follow-Up of Abnormal Mammograms Among Disadvantaged Women. Journal of Women’s Health. 2015;24(2):138-43.

Exclusion reason: Population

1. Percac-Lima S, Ashburner JM, Rigotti N, Park ER, Chang Y, Atlas SJ. Lung Cancer Screening Patient Navigation for Current Smokers in Community Health Centers: A Randomized Controlled Trial. Journal of Clinical Oncology. 2017;35(15).

Exclusion reason: Intervention

1. Percac-Lima S, Ashburner JM, Rigotti NA, Park ER, Atlas SJ. Patient Navigation Program for Lung Cancer Screening in Community Health Centers. Journal of General Internal Medicine. 2017;32(2):782.

Exclusion reason: Intervention

1. Percac-Lima S, Ashburner JM, Rigotti NA, Park ER, Chang Y, Kuchukhidze S, Atlas SJ. Patient Navigation for Lung Cancer Screening among Current Smokers in Community Health Centers: A Randomized Controlled Trial. Cancer Medicine. 2018;7(3):894-902.

Exclusion reason: Intervention

1. Percac-Lima S, Ashburner JM, Zai A, Chang Y, Oo S, Guimarães E, Atlas SJ. Patient Navigation for Comprehensive Cancer Screening in Vulnerable Patients Using a Population-Based Health IT System: Results of a Randomized Control Trial. Journal of General Internal Medicine. 2015;30(2):221.

Exclusion reason: Intervention

1. Percac-Lima S, Ashburner JM, Zai AH, Chang Y, Oo SA, Guimarães E, Atlas SJ. Patient Navigation for Comprehensive Cancer Screening in High-Risk Patients Using a Population-Based Health Information Technology System: A Randomized Clinical Trial. JAMA Internal Medicine. 2016;176(7):930-7.

Exclusion reason: Intervention

1. Percac-Lima S, Hodgkin D, Ashburner JM, Pandharipande P, Park ER, Gorton E. Provider perceptions and costs outcomes of a community-health center based patient navigation program for lung cancer screening. Journal of General Internal Medicine. 2018;33(2 Supplement 1):316.

Exclusion reason: Study design

1. Pescheny JV, Pappas Y, Randhawa G. Facilitators and Barriers of Implementing and Delivering Social Prescribing Services: A Systematic Review. BMC Health Services Research. 2018;18(1):86.

Exclusion reason: Study design

1. Pescheny JV, Randhawa G, Pappas Y. The Impact of Social Prescribing Services on Service Users: A Systematic Review of the Evidence. European Journal of Public Health. 2019;30(4)664-73.

Exclusion reason: Study design

1. Pesut B, Duggleby W, Warner G, Fassbender K, Antifeau E, Hooper B, Greig M, Sullican K. Volunteer Navigation Partnerships: Piloting a Compassionate Community Approach to Early Palliative Care. BMC Palliative Care. 2017;17(1):2.

Exclusion reason: Wrong outcomes

1. Pfeffer GN, Schnack JA. Nurse practitioners as leaders in a quality health care delivery system. Advanced practice nursing quarterly. 1995;1(2):30–9.

Exclusion reason: Study design

1. Phillips RL, Han M, Petterson SM, Makaroff LA, Liaw WR. Cost, Utilization, and Quality of Care: An Evaluation of Illinois' Medicaid Primary Care Case Management Program. Annals of Family Medicine. 2014;12(5):408-17.

Exclusion reason: Study design

1. Phizackerley D. Social Prescribing: Right Idea, Wrong Name? Drug and Therapeutics Bulletin. 2019;57(9):130.

Exclusion reason: Study design

1. Pizzi LT, Jutkowitz E, Frick KD, Suh DC, Prioli KM, Gitlin LN. Cost-effectiveness of a Community-Integrated Home-Based Depression Intervention in Older African Americans. Journal of the American Geriatrics Society. 2014;62(12):2288-95.

Exclusion reason: Wrong outcomes

1. Plant NA, Kelly PJ, Leeder SR, D'Souza M, Mallitt KA, Usherwood T, Jan S, Boyages SC, Essue BM, Mcnab J, Gillespie JA. Coordinated Care Versus Standard Care in Hospital Admissions of People with Chronic Illness: A Randomised Controlled Trial. Medical Journal of Australia. 2015;203(1):33-8.

Exclusion reason: Wrong setting

1. Popejoy LL, Galambos C, Stetzer F, Popescu M, Hicks L, Khalilia MA, Rants Marilyn J, Marek Karen D. Comparing Aging in Place to Home Health Care: Impact of Nurse Care Coordination On Utilization and Costs. Nursing Economics. 2015;33(6):306-13.

Exclusion reason: Intervention

1. Rachwal CM, Langer T, Trainor BP, Bell MA, Browning DM, Meyer EC. Navigating Communication Challenges in Clinical Practice: A New Approach to Team Education. Critical Care Nurse. 2018;38(6):15-22.

Exclusion reason: Population

1. Reinius P, Johansson M, Fjellner A, Werr J, Ohlen G, Edgren G. A Telephone-Based Case Management Intervention Reduces Healthcare Utilization for Frequent Emergency Department Visitors. 2015;20(5):327-34.

Exclusion reason: Intervention

1. Retkin R, Antoniadis D, Pepitone D, Duval D. Legal services: a necessary component of patient navigation. Seminars in Oncology Nursing. 2013; 29(2):149–55.

Exclusion reason: Study design

1. Rice K, Gressard L, DeGroff A, Gersten J, Robie J, Leadbetter S, Kudon Glover R, Butterly L. Increasing Colonoscopy Screening in Disparate Populations: Results from an Evaluation of Patient Navigation in the New Hampshire Colorectal Cancer Screening Program. Cancer. 2017;123(17):3356-66.

Exclusion reason: Study design

1. Ritvo PG, Myers RE, Paszat LF, Tinmouth JM, McColeman J, Mitchell B, Serenity M, Rabeneck L. Personal Navigation Increases Colorectal Cancer Screening Uptake. Cancer Epidemiology, Biomarkers & Prevention. 2015;24(3):506-11.

Exclusion reason: Intervention

1. Robinson MM, Stone G, Tokarz S, Wortham B. Utilizing Actionable Data Analytics to Support Patient Navigation Enrollment and Retention Within Federally Qualified Health Centers. Journal of Public Health Management & Practice. 2017;23: S54-8.

Exclusion reason: Population

1. Rodriguez-Torres SA, McCarthy AM, He W, Ashburner JM, Percac-Lima S. Long-Term Impact of a Culturally Tailored Patient Navigation Program on Disparities in Breast Cancer Screening in Refugee Women After the Program's End. Health Equity. 2019;3(1):205-10.

Exclusion reason: Study design

1. Roeper B, Mocko J, O'Connor LM, Zhou J, Castillo D, Beck EH. Mobile Integrated Healthcare Intervention and Impact Analysis with a Medicare Advantage Population. Population Health Management. 2018;21(5):349-56.

Exclusion reason: Intervention

1. Rohan E, Slotman B, Morrissey KG, Murillo J, DeGroff A, Schroy P. Abstracts of the 2015 World Congress of Psycho-Oncology 28 July - 1 August 2015 Washington, DC, USA. Psycho Oncology. 2015;24 Suppl 2:1-357.

Exclusion reason: Study design

1. Roland M, Everington S, Marshall M. Social Prescribing - Transforming the Relationship between Physicians and Their Patients. The New England Journal of Medicine. 2020;383(2):97-9.

Exclusion reason: Study design

1. Roland M, Paddison C. Better Management of Patients with Multimorbidity. BMJ. 2013.

Exclusion reason: Study design

1. Ruikes FG, Zuidema SU, Akkermans RP, Assendelft WJ, Schers HJ, Koopmans RT. Multicomponent Program to Reduce Functional Decline in Frail Elderly People: A Cluster Controlled Trial. Journal of the American Board of Family Medicine. 2016;29(2):209-17.

Exclusion reason: Intervention

1. Sadarangani T, Missaelides L, Eilertsen E, Jaganathan H, Wu B. A Mixed-Methods Evaluation of a Nurse-Led Community-Based Health Home for Ethnically Diverse Older Adults With Multimorbidity in the Adult Day Health Setting. Policy Polit Nurs Pract. 2019;20(3):131-44.

Exclusion reason: Wrong setting

1. Saillour-Glenisson F, Duhamel S, Fourneyron E, Huiart L, Joseph JP, Langlois E, Pincemail S, Ramel V, Renaud T, Roberts T, Sibe M, Thiessard F, Wittwer J, Salmi Rachid L. Protocole of a controlled before-after evaluation of a national health information technology-based program to improve healthcare coordination and access to information. BMC Health Serv Res. 2017;17(1):297.

Exclusion reason: Study design

1. Salcedo MP, Gowen R, Lopez M, Baker E, Rodriguez AM, Milbourne A, Varon ML, Reininger B, Fernandez ME, Ogburn T, Castle PE, McCormick J, Baker E, Hawk E, Schmeler KM. Addressing the High Cervical Cancer Rates Along the Texas-Mexico Border through Community Outreach, Patient Navigation, and Provider Training/telementoring. Perspectives in Public Health. 2019;154(S1):115.

Exclusion reason: Study design

1. Samaras AT, Murphy K, Nonzee NJ, Endress R, Taylor S, Hajjar N, Bularzik R, Frankovich C, Dong X, Simon Melissa A. Community-Campus Partnership in Action: Lessons Learned from the DuPage County Patient Navigation Collaborative. Progress in Community Health Partnerships: Research, Education and Action. 2014;8(1):75-81.

Exclusion reason: Study design

1. Sandberg M, Kristensson J, Midlov P, Jakobsson U. Effects on Healthcare Utilization of Case Management for Frail Older People: A Randomized Controlled Trial (RCT). Archives of Gerontology and Geriatrics. 2015;60(1):71-81.

Exclusion reason: Intervention

1. Savage RD, Stall NM, Rochon PA. Looking Before We Leap: Building the Evidence for Social Prescribing for Lonely Older Adults. Journal of the American Geriatrics Society. 2020;68(2):429-31.

Exclusion reason: Study design

1. Schillinger D, Domingo KB, Vranizan K, Bacchetti P, Luce JM, Bindman AB. Primary Care At Home Keeps Patients Safe. Case Management Advisor. 2014;25(9):102-4.

Exclusion reason: Study design

1. Schmidt B, Campbell S, McDermott R. Community Health Workers as Chronic Care Coordinators: Evaluation of an Australian Indigenous Primary Health Care Program. Australian and New Zealand Journal of Public Health. 2016;40(S1):107-14.

Exclusion reason: Population

1. Schutt RK, Woodford ML. Increasing Health Service Access by Expanding Disease Coverage and Adding Patient Navigation: Challenges for Patient Satisfaction. BMC Health Services Research. 2020;20(1):175.

Exclusion reason: Intervention

1. Seneviratne MG, Hersch F, Peiris DP. HealthNavigator: A Mobile Application for Chronic Disease Screening and Linkage to Services at an Urban Primary Health Network. Australian Journal of Primary Health. 2018;24(2):116-22.

Exclusion reason: Intervention

1. Shenkman E, Muller K, Vogel B, Nixon SJ, Wagenaar AC, Case K, Guo Y, Wegman M, Aric J, Stoner D. The Wellness Incentives and Navigation Project: Design and Methods. BMC Health Services Research. 2015; 15:579.

Exclusion reason: Population

1. Society of General Internal Medicine. Abstracts SGIM Annual Meeting 2015. Praxis (Bern 1994). 2015; Suppl 1(104):1-213.

Exclusion reason: Study design

1. Shokar NK, Byrd T, Salaiz R, Flores S, Chaparro M, Calderon-Mora J, Reininger B, Dwivewdi A. Against Colorectal Cancer in our Neighbourhoods (ACCION): A Comprehensive Community-Wide Colorectal Cancer Screening Intervention for the Uninsured in a Predominantly Hispanic Community. Preventive Medicine. 2016; 91:273-80.

Exclusion reason: Intervention

1. Shommu NS, Ahmed S, Rumana N, Barron GR, McBrien KA, Turin TC. What Is the Scope of Improving Immigrants and Ethnic Minority Healthcare Using Community Navigators: A Systematic Scoping Review. Internal Journal for Equity in Health. 2016;15(1):6-12.

Exclusion reason: Study design

1. Shroff S, McCoy M, Tuxbury M, Sherman B, Bak S, Battaglia T. Patient navigation for screening mammography: A resident clinic quality improvement initiative. Journal of General Internal Medicine. 2013;28.

Exclusion reason: Intervention

1. Simpson A, Hannigan B, Coffey M, Jones A, Barlow S, Cohen R, Faulkner A, Thornton A, Vseteckova J, Haddad M, Marlowe K. Study Protocol: Cross-National Comparative Case Study of Recovery-Focused Mental Health Care Planning and Coordination (COCAPP). BMC Psychiatry. 2015;15(145).

Exclusion reason: Study design

1. Singal AG, Tiro JA, Murphy CC, Marrero JA, McCallister K, Fullington H, Mejias C, Waljee AK, Bishop WP, Santini NO. Mailed Outreach Invitations Significantly Improve HCC Surveillance Rates in Patients With Cirrhosis: A Randomized Clinical Trial. American Association of the Study of Liver Diseases. 2018;154(6):121-29.

Exclusion reason: Population

1. Slater JS, Parks MJ, Nelson CL, Hughes KD. The Efficacy of Direct Mail, Patient Navigation, and Incentives for Increasing Mammography and Colonoscopy in the Medicaid Population: A Randomized Controlled Trial. Cancer Epidemiology, Biomarkers & Prevention. 2018;27(9):1047-56.

Exclusion reason: Intervention

1. Slazak E, Shaver A, Clark CM, Cardinal C, Poothapattu M, Prescott WA, Jr., Will S, Jacobs DM. Implementation of a Pharmacist-Led Transitions of Care Program within a Primary Care Practice: A Two-Phase Pilot Study. Pharmacy. 2020;8(1).

Exclusion reason: Intervention

1. Smith G, Kirkham R, Gunabarra C, Bokmakarray V, Burgess CP. “We Can Work Together, Talk Together”: An Aboriginal Health Care Home. Journal of the Australian Healthcare & Hospitals Association. 2019;43(5):486-91.

Exclusion reason: Study design

1. Solorio R, Bansal A, Comstock B, Ulatowski K, Barker S. Impact of a Chronic Care Coordinator Intervention on Diabetes Quality of Care in a Community Health Center. Health Services Research. 2015;50(3):730-49.

Exclusion reason: Study design

1. Spano LA, Cocchieri C, Pierangeli M, Tartaglione C, Bolletta E, Scalise A. Case Management of Patients with Chronic Cutaneous Ulcers: Care Pathways and Collaborative Problem Solving. Acta Vulnologica. 2014;12(2):63-70.

Exclusion reason: Population

1. Spiro A, Oo SA, Marable D, Collins JP. A unique model of the community health worker: The MGH Chelsea community health improvement team. Family and Community Health. 2012;35(2):147–60.

Exclusion reason: Study design

1. Srinivasan A, Zwillenberg J, Chadda A, Gissel H, Lettera M, Bender S, Wallhack MK, Srinivasan A. Monitoring and Developing Volunteer Patient Navigation Intervention To Improve Mammography Compliance in a Safety Net Hospital. Journal of Clinical Oncology. 2017;35(8).

Exclusion reason: Intervention

1. Steiner GZ, Ee C, Dubois S, MacMillan F, George ES, McBride KA, Karamacoska D, McDonald K, Harley A, Abramov G, Andrews-Marney ER, Cave AE, Hohenberg MI. “We Need a One-Stop-Shop”: Co-Creating the Model of Care for a Multidisciplinary Memory Clinic with Community Members, GPs, Aged Care Workers, Service Providers and Policy-Makers. BMC Geriatrics. 2020;20(1):1-14.

Exclusion reason: Study design

1. Stergiopoulos V, Gozdzik A, Cohen A, Guimond T, Hwang SW, Kurdyak P, Leszcz M, Wasylenki D. The Effect of Brief Case Management on Emergency Department Use of Frequent Users in Mental Health: Findings of a Randomized Controlled Trial. Plos One. 2017;12(8).

Exclusion reason: Population

1. Suijker JJ, Van Rijn M, Buurman BM, Ter Fiet G, Van Charante M, De Rooij, SE. Effects of Nurse-Led Multifactorial Care to Prevent Disability in Community-Living Older People: Cluster Randomized Trial. Plos One. 2016;11(7).

Exclusion reason: Intervention

1. Sunde S, Walstad RA, Bentsen SB, Lunde SJ, Wangen EM, Rustoen T, Henrikson AH. The Development of an Integrated Care Model for Patients with Severe or Very Severe Chronic Obstructive Pulmonary Disease (COPD): The COPD-Home Model. Scandinavian Journal of Caring Sciences. 2014;28(3):469-77.

Exclusion reason: Population

1. Swanson M, Wong ST, Martin-Misener R, Browne AJ. The Role of Registered Nurses in Primary Care and Public Health Collaboration: A Scoping Review. Nursing Open. 2020;7(4):1197-207.

Exclusion reason: Study design

1. Swift M. People Powered Primary Care: Learning from Halton. Journal of Integrated Care. 2017;25(3):162-73.

Exclusion reason: Study design

1. Tamilselvi V, Cheng L, Hendriks M, Poo R, Tay SM, Vamadevan T. Caring for patients with major psychiatric conditions in the community with case management. Annals of the Academy of Medicine Singapore. 2013;42(8 SUPPL. 1): S27-S8.

Exclusion reason: Population

1. Tanner JA, Black BS, Johnston D, Hess E, Leoutsakos JM, Gitlin LN, Rabins PV, Lyketsos CG. Samus QM. A Randomized Controlled Trial of a Community-Based Dementia Care Coordination Intervention: Effects of MIND at Home on Caregiver Outcomes. The American Journal of Geriatric Psychiatry. 2013;23(4):391-402.

Exclusion reason: Population

1. Tataw DB, Bazargan-Hejazi S, James FW. Health services utilization, satisfaction, and attachment to a regular source of care among participants in an urban health provider alliance. Journal of Health and Human Services Administration. 2011;34(1):109–41.

Exclusion reason: Population

1. Tejeda S, Darnell J, Cho YI, Stolley M, Markossian T, Calhoun E. Patient barriers to follow-up care for breast and cervical cancer abnormalities. Journal of Women’s Health (15409996). 2013;22(6):507–17.

Exclusion reason: Study design

1. Temucin E, Nahcivan NO. Effect of Nurse Navigation Program on Colorectal Cancer Screening Behaviour of the Individuals Aged 50-70. Journal of Global Oncology. 2018;4(Suppl.2):50.

Exclusion reason: Intervention

1. Temucin E, Nahcivan NO. The Effects of the Nurse Navigation Program in Promoting Colorectal Cancer Screening Behaviors: A Randomized Controlled Trial. Journal of Cancer Education. 2020;35(1):112-24.

Exclusion reason: Intervention

1. Tonel P, Byrne D. Care Coordination Partnerships: Promoting Patient Choice in Primary Care. International Journal of Integrated Care. 2017;17(5):1-2.

Exclusion reason: Intervention

1. Torres E, Richman AR, Schreier AM, Vohra N, Verbanac K. An Evaluation of a Rural Community-Based Breast Education and Navigation Program: Highlights and Lessons Learned. Journal of Cancer Education. 2019;34(2):277-84.

Exclusion reason: Intervention

1. Trivedi D, Goodman C, Gage H, Baron N, Scheibl F, Iliffe S, Manthorpe J, Bunn F, Drennan V. The Effectiveness of Inter-Professional Working for Older People Living in the Community: A Systematic Review. Health Social Care in the Community. 2013;21(2):113-28.

Exclusion reason: Study design

1. Trooskin SB, Feller SC, Yolken A, Harvey J, Lee H, Nunn A. Health Care Delivery. Hepatology. 2013;58(S1):1191-213.

Exclusion reason: Study design

1. Tudrej BV, Hervé C, Pradere C, Besse L, Alonso L, Quoirin E, Paccalin. New forms of Allied Healthcare Coordination in Geriatrics. Soins Gerontologie. 2014(107):20-4.

Exclusion reason: Language

1. Uscher-Pines L, Mehrotra A. Analysis of Teladoc use Seems to Indicate Expanded Access to Care for Patients Without Prior Connection to a Provider. Health Affairs. 2014;33(2):258-64.

Exclusion reason: Study design

1. Valaitis RK, Carter N, Lam A, Nicholl J, Feather J, Cleghorn L. Implementation and Maintenance of Patient Navigation Programs Linking Primary Care with Community-Based Health and Social Services: A Scoping Literature Review. BMC Health Services Research.

Exclusion reason: Study design

1. Van Rijn M, Hoogteijling N, Suijker J, De Rooij S, Buurman B, Van Charante EM. Preventive Home-Visits and Nurse-Led Care Coordination: A Qualitative Study on the Experiences, Needs and Preferences of Community Dwelling Older People. European Geriatric Medicine. 2016.

Exclusion reason: Intervention

1. Vasyushkina MA. The Multidisciplinary Case Management Team as an Effective Model for Providing Modern Psychiatric Care Under Compulsory Treatment Conditions. International Journal of Culture and Mental Health. 2017;11(1):120-1.

Exclusion reason: Study design

1. Verma M, Punyani H, Kalra S. Social Prescription in Diabetes. Journal of Pakistan Medical Association. 2019;69(12):1922-23.

Exclusion reason: Intervention

1. Vimalananda VG, Dvorin K, Fincke BG, Tardiff N, Bokhour BG. Patient, Primary Care Provider, and Specialist Perspectives on Specialty Care Coordination in an Integrated Health Care System. The Journal of Ambulatory Care Management. 2018;41(1):15-24.

Exclusion reason: Population

1. Vora S, Lau JD, Kim E, Sim SC, Oster A, Pong P. Patient Navigation Program for Colorectal Cancer Screening in Chinese Americans at an Urban Community Health Center: Lessons Learned. J Health Care Poor Underserved. 2017;28(3):887-95.

Exclusion reason: Study design

1. Wallace E, Smith SM, Fahey T, Roland M. Reducing Emergency Admissions Through Community Based Interventions. BMJ. 2016;352

Exclusion reason: Study design

1. Wallis L, Co-Management Improves Geriatric Care. American Journal of Nursing. 2013;113(10):16-.

Exclusion reason: Study design

1. Wang P, Sewanan LR, Duffy E, Meizlish M, Berk-Krauss J, Gamez S. Meeting Patients Where They Are: Enhancing Competence In Addressing Social Barriers To Care Through A Medical Student Patient Navigator Program. Journal of General Internal Medicine. 2019;34(2): S830.

Exclusion reason: Study design

1. Ward A, Asif A, Cattermole R, Chima J, Ebbatson T, Mahi I, Richardson N, Sheikh H. Social Prescribing by Students: The Design and Delivery of a Social Prescribing Scheme By Medical Students in General Practice. Education for Primary Care. 2020;(5)1-5.

Exclusion reason: Study design

1. Wardle B, Birch S, Reeves T. STD and Viral Hepatitis Screening and Testing Among Ryan White Clients in a Case Management Setting. Sexually Transmitted Diseases. 2018;45.

Exclusion reason: Intervention

1. White EM, Kosar CM, Rahman M, Mor V. Trends in Hospitals and Skilled Nursing Facilities Sharing Medical Providers, 2008-16. Health Affairs 2020;39(8):1312-20.

Exclusion reason: Intervention

1. Whitelaw S, Thirlwall C, Morrison A, Osborne J, Tattum L, Walker S. Developing and Implementing a Social Prescribing Initiative in Primary Care: Insights Into the Possibility of Normalisation and Sustainability from a UK Case Study. Cambridge. 2017;18(2):112-21.

Exclusion reason: Wrong outcomes

1. Williams VS, Smith A, Chapman L, Oliver D. Community matrons – an exploratory study of patients’ views and experiences. Journal of Advanced Nursing. 2010;1(67):86–93.

Exclusion reason: Study design

1. Wilson A, O'Hare JP, Hardy A, Raymond N, Szczepura A, Crossman R, Baines D, Khunti K, Kumar S, Saravanan P. Evaluation of the Clinical and Cost Effectiveness of Intermediate Care Clinics for Diabetes (ICCD): A Multicentre Cluster Randomised Controlled Trial. PLOS ONE. 2014;9(4): e93964.

Exclusion reason: Population

1. Yang Y, Dillon EC, Li M, Li J, Erlich KJ, Heneghan AM, Becker DF. Primary Care Provider Utilization and Satisfaction with a Health System Navigation Program for Adolescents with Behavioral Health Needs. Translational Behavioral Medicine. 2019;9(3):549-59.

Exclusion reason: Population

1. Yee LM, Martinez NG, Nguyen AT, Hajjar N, Chen MJ, Simon MA. Using a Patient Navigator to Improve Postpartum Care in an Urban Women's Health Clinic. Obstetrics & Gynecology. 2017;129(5):925-33.

Exclusion reason: Study design
