# Supplementary material for: Effectiveness of system navigation programs linking primary care with community-based health and social services: a systematic review
Source: BMC Health Serv Res. 2023 May 8;23:450. doi: 10.1186/s12913-023-09424-5 (PMC10165767; doi:10.1186/s12913-023-09424-5)
Supplement: Supplementary file 3 — Additional file 3. JBI Critical Appraisal Checklist for Randomized Controlled Trials. [file 12913_2023_9424_MOESM3_ESM.docx]

# **Additional file 3: JBI Critical Appraisal Checklist for Randomized Controlled Trials (n=8)**

| **Study** | **Q1** | **Q2** | **Q3** | **Q4** | **Q5** | **Q6** | **Q7** | **Q8** | **Q9** | **Q10** | **Q11** | **Q12** | **Q13** | **Score By Study** |
| --- | --- | --- | --- | --- | --- | --- | --- | --- | --- | --- | --- | --- | --- | --- |
| Boult 2013 | Y | Y | N | Y | N | N | Y | N | U | Y | Y | U | Y | **7/13** |
| Dolovich 2016 | Y | Y | Y | N | Y | N | N | Y | Y | Y | Y | Y | Y | **10/13** |
| Kangovi 2016 | Y | Y | Y | N | N | N | Y | Y | Y | Y | Y | Y | U | **9/13** |
| Kangovi 2018 | Y | Y | Y | Y | N | N | Y | Y | Y | Y | Y | Y | Y | **11/13** |
| Mercer 2019 | U | U | N | Y | N | N | N | N | Y | Y | N | Y | Y | **5/13** |
| Spoorenberg 2018 | Y | Y | Y | Y | N | N | Y | Y | Y | Y | Y | Y | Y | **11/13** |
| Taube 2018 | Y | N | Y | Y | N | N | N | Y | Y | Y | Y | Y | Y | **9/13** |
| Zhang 2018 | Y | N | U | Y | N | N | U | N | U | Y | U | N | Y | **4/13** |
| **Total (%)** | **87.5** | **62.5** | **62.5** | **75.0** | **12.5** | **0.0** | **50.0** | **62.5** | **75.0** | **100.0** | **75.0** | **75.0** | **87.5** |  |
| Note: Y, yes. U, unclear. N, no. 1. Was true randomization used for assignment of participants to treatment groups? 2. Was allocation to treatment groups concealed? 3. Were treatment groups similar at baseline? 4. Were treatment groups treated identically other than the intervention of interest? 5. Were participants blind to treatment assignment? 6. Were those delivering treatment blind to treatment assignment? 7. Were outcomes assessors blind to treatment assignment? 8. Was follow up complete and if not, were differences between groups in terms of their follow up adequately described and analyzed? 9. Were participants analyzed in the groups to which they were randomized? 10. Were outcomes measured in the same way for treatment groups? 11. Were outcomes measured in a reliable way? 12. Was appropriate statistical analysis used? 13. Was the trial design appropriate for the topic, and any deviation from the standard RCT design accounted for in the conduct and analysis? | | | | | | | | | | | | | | |

# 
