# Supplementary material for: Effectiveness of system navigation programs linking primary care with community-based health and social services: a systematic review
Source: BMC Health Serv Res. 2023 May 8;23:450. doi: 10.1186/s12913-023-09424-5 (PMC10165767; doi:10.1186/s12913-023-09424-5)
Supplement: Supplementary file 4 — Additional file 4. JBI Critical Appraisal Checklist for Quasi-Experimental Studies. [file 12913_2023_9424_MOESM4_ESM.docx]

# **Additional file 4: JBI Critical Appraisal Checklist for Quasi-Experimental Studies (n=13)**

| **Study** | **Q1** | **Q2** | **Q3** | **Q4** | **Q5** | **Q6** | **Q7** | **Q8** | **Q9** | **Score By Study** |
| --- | --- | --- | --- | --- | --- | --- | --- | --- | --- | --- |
| Burger 2019 | Y | Y | Y | N | Y | N | Y | Y | N | **6/9** |
| Carnes 2017 | Y | N | Y | Y | Y | N | Y | Y | Y | **7/9** |
| Dye 2018 | Y | U | U | Y | U | N | Y | Y | U | **4/9** |
| Franse 2018 | Y | N | Y | Y | Y | N | Y | Y | Y | **7/9** |
| Kellezi 2019 | Y | Y | Y | N | Y | N | Y | Y | Y | **7/9** |
| Loftus 2017 | N | Y | Y | Y | N | Y | N | Y | Y | **6/9** |
| Loskutova 2016 | Y | Y | Y | N | Y | N | Y | N | Y | **6/9** |
| Mayhew 2019 | Y | Y | Y | N | Y | Y | Y | Y | Y | **8/9** |
| Pescheny 2019 | Y | Y | U | N | N | N | Y | Y | Y | **5/9** |
| Tung 2020 | Y | Y | N | Y | Y | Y | Y | Y | Y | **8/9** |
| Vanderboom 2014 | Y | N | Y | Y | Y | U | Y | Y | Y | **7/9** |
| Wang 2015 | Y | U | Y | N | Y | N | Y | Y | Y | **6/9** |
| Woodall 2018 | Y | Y | Y | N | Y | N | Y | Y | U | **6/9** |
| **Total (%)** | **92.3** | **61.5** | **76.9** | **46.2** | **76.9** | **23.1** | **92.3** | **92.3** | **76.9** |  |
| Note: Y, yes. U, unclear. N, no. 1. Is it clear in the study what is the “cause” and what is the “effect” (i.e., there is no confusion about which variable comes first)? 2. Were the participants included in any comparisons similar? 3. Were the participants included in any comparisons receiving similar treatment/care, other than the exposure or intervention of interest? 4. Was there a control group? 5. Were there multiple measurements of the outcome both pre and post the intervention/exposure? 6. Was follow up complete and if not, were differences between groups in terms of their follow up adequately described and analyzed? 7. Were the outcomes of participants included in any comparisons measured in the same way? 8. Were outcomes measured in a reliable way? 9. Was appropriate statistical analysis used? | | | | | | | | | | |


# 
