# Supplementary material for: Effectiveness of system navigation programs linking primary care with community-based health and social services: a systematic review
Source: BMC Health Serv Res. 2023 May 8;23:450. doi: 10.1186/s12913-023-09424-5 (PMC10165767; doi:10.1186/s12913-023-09424-5)
Supplement: Supplementary file 5 — Additional file 5. Health Service Utilization Outcomes. [file 12913_2023_9424_MOESM5_ESM.docx]

# **Additional file 5: Health Service Utilization Outcomes (n=13)**

| **Study** | **Description of Intervention/Comparator** | **Outcome (Tool)** | **Effect and significance** | | **Risk of Bias Score** |
| --- | --- | --- | --- | --- | --- |
| **Lay person-led system navigation model** | | | | | |
| **Burger 2019** | I: Health-coach led self-management including care team communication, scheduling reminders, medication refills, referral to social services, emotional support and review of care plans.  C: Baseline | Scheduled primary care visits, last 6 months (EHR) | -4 visits, p-value NR | | 6/9 |
|  |  | Appointment no-shows, last 6 months (EHR) | -1 visits, p-value NR | |  |
|  |  | ED or hospital admission, last 6 months (EHR) | -1 admission, p-value NR | |  |
|  |  | Acute care visits, last 6 months (EHR) | -3 visits, p-value NR | |  |
| **Dye 2018** | I: Volunteer health coach intervention, including needs assessment, home visits, self-management, education on use of self-monitoring equipment, linking to external services based on client needs.  C: Matched patients who chose not to participate | % Receiving emergent care for at least one home health service diagnosis during intervention (patient records) | I: 42.4% C: 61.1%, p-value NR | | 4/9 |
|  |  | % With ED/hospital visit at 90-days (patient records) | I: 10%  C: 24%, p-value NR | |  |
|  |  | % With ED/hospital visit at 180-days (patient records) | I: 19%  C: 44%, p-value NR | |  |
|  |  | Rate of ED/hospital visits per patient at 90-days (patient records) | I: 0.13  C: 0.28, p = 0.65 | |  |
|  |  | Rate of ED/hospital visits per patient at 180-days (patient records) | I: 0.29  C: 0.72, p= 0.19 | |  |
| **Kangovi 2016** | I: Goal setting plus IMPaCT, standardized intervention led by community health workers. Includes tailored coaching, social support, navigation, and advocacy.  C: Goal setting plus UC | Number of primary care appointments, last 6 months (EHR) | MD: -0.8 (SD NR), p = 0.57 | | 9/13 |
|  |  | % Hospitalized, during 6-month intervention (EHR) | I: 16%  C: 17.8%, p = 0.68 | |  |
|  |  | % Hospitalized, at 12-month follow-up (EHR) | I: 23.3%  C: 31.6%, p = 0.11 | |  |
|  |  | Number of all-cause hospitalizations, last 12 months (EHR) | I: 68  C: 98, p = 0.17 | |  |
|  |  | Number of hospital-days, last 12 months (EHR) | I: 278  C: 414, p = 0.17 | |  |
| **Kangovi 2018** | I: Goal setting plus IMPaCT, standardized intervention led by community health workers. Includes tailored coaching, social support, navigation, and advocacy.  C: Goal setting plus UC | Risk of hospital admission during intervention period (administrative data) | Risk difference: -0.01, 95% CI -0.06, 0.05 | | 11/13 |
|  |  | Risk of repeat admission during intervention period (administrative data), *I vs. C* | **OR: 0.4, 95% CI 0.2, 0.9**  **Risk difference: -0.24, 95% CI: -0.40, -0.07** | |  |
|  |  | Risk of 30-day readmission during intervention period (administrative data), *I vs. C* | **OR: 0.3, 95% CI 0.1, 0.9**  **Risk difference: -0.17, 95% CI −0.32, −0.02** | |  |
|  |  | Total hospital days at end of 9-month intervention (administrative data) | I: 300 days  C: 471 days  Absolute reduction: 65%, p-value NR | |  |
|  |  | Number of hospitalizations during intervention period (administrative data) | MD: -0.3, 95% CI -0.6, 0.0 | |  |
|  |  | Mean length of stay (administrative data) | MD: -3.1, 95% CI -6.3, 0.2 | |  |
| **Mayhew 2009** | I: Integrated Care Coordination Service led by a care coordinator, includes identification of needs and liking to relevant health, social security or other organizations.  C: Baseline | Trend in actual admissions 9 months post-integrated care coordination service referral (source NR) | **Slope -0.1467, standard error 0.029, p = 0.01** | | 8/9 |
|  |  | Trend in actual bed-day utilization 9 months post-integrated care coordination service (source NR) | **Slope -0.1105, standard error 0.025, p = 0.01** | |  |
|  |  | Trend in actual ED attendance 9 months post-integrated care coordination service (source NR) | **Slope -0.1136, standard error 0.0217, p = 0.01** | |  |
| **Wang 2015** | I: Community health worker-led patient navigation including education, appointment scheduling, assistance with overcoming barriers to health care access.  C: Participants not reached by patient navigators | Rates of visits with primary care provider and/or chronic disease nurse visit, last 6 months (EHR) *I vs. C* | **Higher rates of visits, p < 0.05** | | 6/9 |
|  |  | % patients who visited primary care provider first before seeing other providers (EHR) | **I: 64%**  **C: 27.6%, p < 0.0001** | |  |
| **Health professional-led system navigation model** | | | | | |
| **Boult 2013** | I: Nurse-led Guided Care intervention including assessment of patient needs, care-planning and coordination, transitional care, monitoring, self-management, caregiver support and access to community-based services.  C: UC | Mean annual per capita rate of primary care visits (health insurance data) | | Rate Ratio: 0.99, 95% CI 0.82, 1.18 | 7/13 |
|  |  | Mean annual per capita rate of hospital admissions (health insurance data) | | Rate ratio 0.94, 95% CI 0.74, 1.19 |  |
|  |  | Mean annual per capita rate of 30-day hospital re-admissions (health insurance data) | | Rate ratio 0.87, 95% CI 0.53, 1.41 |  |
|  |  | Mean annual per capita rate of ED visits (health insurance data) | | Rate ratio 1.02, 95% CI 0.78, 1.33 |  |
|  |  | Mean annual per capita rate of specialist visits (health insurance data) | | Rate ratio: 1.02, 95% CI 0.86, 1.22 |  |
|  |  | Mean annual per capita rate of home health care episodes (health insurance data) | | **Rate ratio 0.71, 95% CI 0.51, 0.97** |  |
|  |  | Mean annual per capita rate of skilled nursing facility days (health insurance data) | | Rate ratio 0.74, 95% CI 0.38, 1.44 |  |
| **Franse 2018** | I: Care coordinator-led (variable by setting including social worker, nurse, nurse practitioner, physician assistant) Urban Health Centres Europe approach, including health assessment, shared decision making in development of care plan and referral to appropriate care pathways including health and social services.  C: UC | Number of visits to a medical doctor, last 12 months (self-report) | | MD: -0.25, 95% CI: -0.90, 0.41 | 7/9 |
|  |  | Number of days admitted to hospital, last 12 months (self-report) | | MD: 0.24, 95% CI -0.30, 0.77 |  |
|  |  | Hours per week receiving help in caring for oneself, last 12-months (self-report) | | MD: -0.01, 95% CI -0.23, 0.20 |  |
|  |  | Hours per week receiving help in household work, last 12-months (self-report) | | **MD: -0.39, 95% CI -0.75, -0.04** |  |
| **Loftus 2017** | I: Social worker-led social prescribing activities focused on health and well-being, emotional and practical support, education and self-help.  C: Patients who declined to participate | % patients ≥1 primary care visit, last 3 months (tool NR) | | **-16.2%, p = 0.021** | 6/9 |
|  |  | % patients ≥1 primary care home visit, last 3 months (tool NR) | | +5.9%, p = 0.32 |  |
|  |  | % patients ≥1 primary care telephone visit, last 3 months (tool NR) | | -13.2, p = 0.14 |  |
|  |  | % Patients with 0 or 1 primary care contacts, last 3 months (tool NR) | | No change, p = 1.0 |  |
|  |  | % Patients with no new repeat medications, last 3 months (tool NR) | | -7.4%, p = 0.38 |  |
| **Vanderboom 2014** | I: Nurse-led Community Connections Program, including strengths assessment, action planning, crisis prevention plan, and circle of support, comprised of community and informal resources for self-management.  C: UC | Number of primary care visits, last 3 months (Health Service Utilization Questionnaire) | | NS change, values NR | 7/9 |
|  |  | ED/urgent care, hospital stays, last 3 moths (Health Service Utilization Questionnaire) | | NS change, values NR |  |
|  |  | Home health care use, last 3 months (Health Service Utilization Questionnaire) | | NS change, values NR |  |
| **Team-based system navigation model** | | | | | |
| **Carnes 2017** | I: Social prescribing service coordinated by social workers with volunteer support, including action planning and referral to community services.  C: Matched patients from neighbouring area | Annual GP consultation rate (health records) | | ***Pre-intervention (median, IQR)***  **I: 8.3 (5.8-12.1)**  **C: 2.9 (0.6-5.8), between-group difference p <0.001**  ***Post-intervention (median, IQR)***  **I: 7.3 (4.7-10.7)**  **C: 3.3 (0-6.4), between-group difference p <0.0001** | 7/9 |
|  |  | ED visits, last 3 months (health records) | | *Pre-intervention*  I: NR  C: 0.3, SD 0.79  *Post-intervention*  I: 0.3, SD 0.68 C: 0.5, SD 1.15, NS |  |
| **Dolovich 2016** | I: Health TAPESTRY, volunteer-led home visit to assess health status and goals, action planning with healthcare team including links to community support.  C: Wait-list control (UC) | Primary care visits, last 6 months (EMR) | | **MD: 1.52, 95% CI 0.84, 2.19** | 10/13 |
|  |  | % Hospitalized, last 6 months (EMR) *None vs. 1+* | | **OR: 0.44, 95% CI 0.20, 0.95** |  |
|  |  | Mean number of hospitalizations, last 6 months (EMR) | | **Incidence Rate Ratio: 0.37, 95% CI 0.18, 0.77** |  |
|  |  | % Visited ER, last 6 months (EMR) *None vs. 1+* | | OR: 0.58, 95% CI 0.28, 1.20 |  |
|  |  | Mean number of ER visits, last 6 months (EMR) | | Incidence Rate Ratio: 0.69, 95% CI 0.34, 1.43 |  |
| **Kellezi 2019** | I: Health coach and link worker-led intervention that involved a needs assessment and then subsequent referral to relevant third sector groups.  C: Baseline | Primary care use, last 3 months (self-report) | | **Pre-intervention: 5.9, SD 8.2**  **Post-intervention: 4.5, SD 8.4, p = 0.003** | 7/9 |

| Note: **Bold text indicates significant difference.** C = comparator group, CI = confidence interval, ED/ER = emergency department/room, EHR = electronic health record, EMR = electronic medical record, GP = general practitioner, I = intervention group, IMPaCT = Individualized Management for Patient-Centered Targets, IQR = interquartile range, MD = mean difference, NR = not reported, NS = not statistically significant, OR = odds ratio, SD = standard deviation, UC = usual care |
| --- |


# 
