# Supplementary material for: Effectiveness of system navigation programs linking primary care with community-based health and social services: a systematic review
Source: BMC Health Serv Res. 2023 May 8;23:450. doi: 10.1186/s12913-023-09424-5 (PMC10165767; doi:10.1186/s12913-023-09424-5)
Supplement: Supplementary file 6 — Additional file 6. Patient-Related Outcomes. [file 12913_2023_9424_MOESM6_ESM.docx]

# **Additional file 6: Patient-Related Outcomes**

| **Study** | **Description of Intervention/Comparator** | **Outcome (Tool)** | **Effect and significance** | **Risk of Bias Score** | |
| --- | --- | --- | --- | --- | --- |
| **QUALITY OF LIFE/HEALTH RELATED QUALITY OF LIFE, MENTAL HEALTH, AND WELLBEING OUTCOMES (n=13)** | | | | | |
| **Lay person-led system navigation model** | | | | | |
| **Kangovi 2016** | I: Goal setting plus IMPaCT, standardized intervention led by community health workers. Includes tailored coaching, social support, navigation, and advocacy.  C: Goal setting plus UC | Health-related QoL, physical (SF-12) | MD: 0.4 (SD NR), p = 0.67 | 9/13 | |
|  |  | Health-related QoL, mental (SF-12) | **MD: 2.5 (SD NR), p = 0.008** |  |  |
| **Kangovi 2018** | I: Goal setting plus IMPaCT, standardized intervention led by community health workers. Includes tailored coaching, social support, navigation, and advocacy.  C: Goal setting plus UC | Health-related QoL, physical (SF-12) | MD: -0.7, 95% CI -2.2, 0.7 | 11/13 | |
|  |  | Health-related QoL, mental (SF-12) | MD: 0.8, 95% CI -1.1, 2.6 |  |  |
| **Mercer 2019** | I: Community Links Practitioner intervention including assessing patient needs, linking to community organizations and if necessary, providing support to ensure attendance.  C: UC | Health-related QoL (EQ-5D-5L) | Adjusted MD: 0.008, 95% CI –0.028, 0.045 | 5/13 | |
|  |  | Wellbeing (Investigating Choice Experiments for the Preferences of Older People Capability Measure for Adults, interpretation NR) | Adjusted MD: –0.011, 95% CI –0.039, 0.016 |  |  |
|  |  | Anxiety (HADS where 0-7 is normal, 8-10 is borderline, and 11-21 is abnormal) | Adjusted MD: -0.41, 95% CI -0.99, 0.18 |  |  |
|  |  | Depression (HADS where 0-7 is normal, 8-10 is borderline, and 11-21 is abnormal) | Adjusted MD: 0.09, 95% CI -0.49, 0.68 |  |  |
| **Pescheny 2019** | I: Social prescribing service led by trained non-clinicians that linked patients in primary care with sources of support within the community sector to improve their health, well-being, and care experience.  C: Baseline | Wellbeing (Warwick-Edinburg Mental Wellbeing Scale where higher score indicates better mental wellbeing, and a minimal clinically significant difference is 3 points) | **MD: 2.78, 95% CI 1.68, 3.88** | 5/9 | |
| **Woodall 2018** | I: Social prescribing via wellbeing coordinators, including needs assessment and referral to local community health and wellbeing resources.  C: Baseline | Wellbeing (Warwick-Edinburgh Mental Wellbeing Scale where higher score indicates better mental wellbeing, and a minimal clinically significant difference is 3 points) | **MD: 3.98, 95% CI 3.41, 4.55**  *Note: Size of improvement medium to large* | 6/9 | |
|  |  | Anxiety and depression (EQ-5D) | **“Lower than at baseline”, p < 0.001** |  |  |
|  |  | Health rating (scale of 1-100: 0 was the ‘worst health you can image’ and 100 was the ‘best health you can imagine’) | **MD: 9.56, 95% CI 7.09, 12.02**  *Note: Size of improvement small to medium* |  |  |
| **Health professional-led system navigation model** | | | | | |
| **Boult 2013** | I: Nurse-led Guided Care intervention including assessment of patient needs, care-planning and coordination, transitional care, monitoring, self-management, caregiver support and access to community-based services.  C: UC | Health-related QoL, physical (SF-36) | Adjusted MD: -1.31, 95% CI -3.02, 0.41 | 7/13 | |
|  |  | Health-related QoL, mental health (SF-36) | Adjusted MD: 1.05, 95% CI -1.08, 3.12 |  |  |
|  |  | Self-rated health (Single item; “excellent, very good, or good”) *I vs. C* | OR: 0.89, 95% CI 0.61, 1.33 |  |  |
| **Franse 2018** | I: Care coordinator-led (variable by setting including social worker, nurse, nurse practitioner, physician assistant) Urban Health Centres Europe approach, including health assessment, shared decision making in development of care plan and referral to appropriate care pathways including health and social services.  C: UC | Health-related QoL, physical (SF-12) | **MD: 0.95, 95% CI 0.14, 1.76** | 7/9 | |
|  |  | Health-related QoL, mental (SF-12) | MD: 0.52, 95% CI -0.32, 1.37 |  |  |
|  |  | Health-related QoL, mental health (SF-36) | **MD: 1.50, 95% CI 0.15, 2.84** |  |  |
| **Vanderboom 2014** | I: Nurse-led Community Connections Program, including strengths assessment, action planning, crisis prevention plan, and circle of support, comprised of community and informal resources for self-management.  C: UC | Mobility (EQ-5D) | MD: 0.11 (SD NR), p=0.42 | 7/9 | |
|  |  | Self-care (EQ-5D) | MD: -0.04 (SD NR), p=0.79 |  |  |
|  |  | Usual activities (EQ-5D) | MD: -0.18 (SD NR), p=0.2 |  |  |
|  |  | Pain/discomfort (EQ-5D) | MD: -0.01 (SD NR), p=0.86 |  |  |
|  |  | Anxiety/depression (EQ-5D) | MD: -0.11 (SD NR), p=0.48 |  |  |
| **Team-based system navigation model** | | | | | |
| **Carnes 2017** | I: Social prescribing service coordinated by social workers with volunteer support, including action planning and referral to community services.  C: Matched patients from neighbouring area | General Health Score (Scale of 1-5 where 1 is “very bad” and 5 is “very good”) | Adjusted MD: 0.127, 95% CI -0.221, 0.475 | 7/9 | |
|  |  | Wellbeing, past week (Measure Yourself Medical Outcome Profile, range 0-6) | Adjusted MD: -0.013, 95% CI -0.623, 0.596 |  |  |
|  |  | Anxiety (HADS, where 0-7 is normal, 8-10 is borderline, and 11-21 is abnormal) | Adjusted MD: -0.119, 95% CI −0.847, 1.609 |  |  |
|  |  | Depression (HADS, where 0-7 is normal, 8-10 is borderline, and 11-21 is abnormal) | Adjusted MD 0.857, 95% CI −0.737, 2.451 |  |  |
| **Dolovich 2016** | I: Health TAPESTRY, volunteer-led home visit to assess health status and goals, action planning with healthcare team including links to community support.  C: Wait-list control (UC) | Quality of life (EQ-5D-5L) | MD: 0.02, 95% CI -0.014, 0.058 | 10/13 | |
| **Spoorenberg 2018** | I: Embrace, population-based integrated elderly care model (physician, nurse, social worker) including self-management support, introduction to community resources, and case management for those with complex care needs.  C: UC | Health-related QoL (EQ-5D-3L) | MD: 0, 95% CI -0.01, 0.01 | 11/13 | |
|  |  | Self-rated health (EQ-VAS) | MD: 0.1, 95% CI -1.21, 1.42 |  |  |
|  |  | Complexity of care needs (INTERMED-E-SA) | MD: 0.12, 95% CI -0.31, 0.54 |  |  |
|  |  | Wellbeing (Groningen Wellbeing Indicator Satisfaction Score) | MD: 0, 95% CI -0.02, 0.02 |  |  |
|  |  | Health-related QoL (RAND-36) | MD: -0.02, 95% CI -0.09, 0.06 |  |  |
|  |  | Self-management ability (Self-Management Ability Score-30) | MD: -0.34, 95% CI -1.16, 0.48 |  |  |
|  |  | Self-management knowledge and behaviour (Partners in Health scale for older adults) | MD: 0.45, 95% CI -0.37, 1.28 |  |  |
| **Taube 2018** | I: Registered nurse and physical therapist-led case management including monthly home visit, care plan development, healthcare system navigation, health information, information about local activities.  C: UC | Life satisfaction (Life Satisfaction Index-Z, range 0-26 where higher score indicates greater satisfaction) | Cohen’s d (mid-point): 0.06, p=0.326  Cohen’s d (end-of-study): 0.07, p=0.906 | 9/13 | |
|  |  | Depression (Geriatric Depression Scale-20, range 0-20 where 6 indicates risk of depression) | Cohen’s d (mid-point): 0.21, p= 0.643  Cohen’s d (end-of-study): 0.02, p= 0.314 |  |  |
| **Self-navigation with lay support as needed system navigation model** | | | | | |
| **Tung 2020** | I: “HealtheRx” intervention, electronic-medical record generated personalized list of local community resources with access to community health information specialist as needed.  C: UC | Health-related QoL, mental (SF-12) | MD: -1.03, 95% CI -3.02, 0.96 | 8/9 | |
|  |  | Health-related QoL, physical (SF-12) | MD: 0.59, 95% CI -0.98, 2.16 |  |  |
| **SOCIAL PARTICIPATION AND FUNCTION OUTCOMES (n=8)** | | | | | |
| **Lay person-led system navigation model** | | | | | |
| **Mercer 2019** | I: Community Links Practitioner intervention including assessing patient needs, linking to community organizations and if necessary, providing support to ensure attendance.  C: UC | Work and Social Adjustment (Work and Social Adjustment Scale, range 0-40 where 0 -9 is low impairment, 10-19 is moderate impairment, and 20-40 is severe impairment) | MD: 0.05, 95% CI –1.37, 1.48 | 5/13 | |
| **Woodall 2018** | I: Social prescribing via wellbeing coordinators, including needs assessment and referral to local community health and wellbeing resources.  C: Baseline | Social networks (Campaign to End Loneliness Measurement Tool, range 0-12 where 0 is least lonely and 12 is most lonely)) | **MD: 0.83, 95% CI 0.57, 1.1** | 6/9 | |
| **Health professional-led system navigation model** | | | | | |
| **Franse 2018** | I: Care coordinator-led (variable by setting including social worker, nurse, nurse practitioner, physician assistant) Urban Health Centres Europe approach, including health assessment, shared decision making in development of care plan and referral to appropriate care pathways including health and social services.  C: UC | Loneliness (6-item Jong Giervald Loneliness Scale) | MD: −0.10, 95% CI −0.24, 0.03 | 7/9 | |
| **Vanderboom 2014** | I: Nurse-led Community Connections Program, including strengths assessment, action planning, crisis prevention plan, and circle of support, comprised of community and informal resources for self-management.  C: UC | Social health (Satisfaction with Participation in Social Roles) | MD: -0.36 (SD NR), p=0.97 | 7/9 | |
|  |  | Social health (Satisfaction with Participation in Discretionary Social Activities) | MD: -0.25 (SD NR), p=0.36 |  |  |
| **Team-based system navigation model** | | | | | |
| **Carnes 2017** | I: Social prescribing service coordinated by social workers with volunteer support, including action planning and referral to community services.  C: Matched patients from neighbouring area | Active engagement in life (Health Education Impact Questionnaire, range 5-20 where 5 is poorly integrated and 20 is well integrated) | Adjusted MD: -0.073, 95% CI -1.278, 1.131 | 7/9 | |
| **Dolovich 2016** | I: Health TAPESTRY, volunteer-led home visit to assess health status and goals, action planning with healthcare team including links to community support.  C: Wait-list control (UC) | Social network score (Duke Social Support Index) | MD: 0.038, 95% CI -0.25, 0.33 | 10/13 | |
|  |  | Social satisfaction score (Duke Social Support Index) | MD: 0.102, 95% CI -0.35, 0.55 |  |  |
| **Kellezi 2019** | I: Health coach and link worker-led intervention that involved a needs assessment and then subsequent referral to relevant third sector groups.  C: Baseline | Number of social group memberships | **T0 (baseline): 1.89, SD 1.59**  **T1 (end-of-study): 2.21, SD 1.87**  **p=0.022** | 7/9 | |
|  |  | Community belonging (Single item) | T0 (baseline): 2.72, SD 1.12 T1 (end-of-study): 2.75, SD 1.08  p-value NR |  |  |
|  |  | Loneliness (UCLA Loneliness Scale) | T0 (baseline): 2.42, SD 1.13 T1 (end-of-study): 2.35, SD 1.00  p-value NR |  |  |
| **Taube 2018** | I: Registered nurse and physical therapist-led case management including monthly home visit, care plan development, healthcare system navigation, health information, information about local activities.  C: UC | Loneliness (Single item, “Not lonely” vs. “lonely”) | Relative risk (mid-point): 0.15, p=0.206 Relative risk (end-of-study): 0.13, p=0.587 | 9/13 | |
| **HEALTH BEHAVIOUR OUTCOMES (n=7)** | | | | | |
| **Lay person-led system navigation model** | | | | | |
| **Kangovi 2016** | I: Goal setting plus IMPaCT, standardized intervention led by community health workers. Includes tailored coaching, social support, navigation, and advocacy.  C: Goal setting plus UC | Cigarettes per day | MD: -4.3, 95% CI –9.3, 0.7 | 9/13 | |
| **Kangovi 2018** | I: Goal setting plus IMPaCT, standardized intervention led by community health workers. Includes tailored coaching, social support, navigation, and advocacy.  C: Goal setting plus UC | Cigarettes per day | MD: -0.5, 95% CI -2.2, 1.2 | 11/13 | |
| **Mercer 2019** | I: Community Links Practitioner intervention including assessing patient needs, linking to community organizations and if necessary, providing support to ensure attendance.  C: UC | Exercise level (Self-reported, unit NR) | Adjusted MD: 0.12, 95% CI –0.06, 0.3 | 5/13 | |
| **Pescheny 2019** | I: Social prescribing service led by trained non-clinicians that linked patients in primary care with sources of support within the community sector to improve their health, well-being, and care experience.  C: Baseline | Physical activity (International Physical Activity Questionnaire) | **Total MET (min/week): Mean +56.3%, 95% PPI 54.77%, 57.69%** | 5/9 | |
|  |  |  | **Moderate MET (min/week): Mean +5.0%, 95% PPI 2.94%, 7.09%** |  |  |
|  |  |  | **Vigorous MET (min/week): Mean +107.3%, 95% PPI 98.19%, 116.20%** |  |  |
|  |  |  | **Walking MET (min/week): Mean +41.7%, 95% PPI 40.31%, 43.11%** |  |  |
| **Health professional-led system navigation model** | | | | | |
| **Franse 2018** | I: Care coordinator-led (variable by setting including social worker, nurse, nurse practitioner, physician assistant) Urban Health Centres Europe approach, including health assessment, shared decision making in development of care plan and referral to appropriate care pathways including health and social services.  C: UC | Healthy lifestyle (No smoking, no drinking, and exercise > 1 times per week), *I vs. C* | OR 0.96, 95% CI 0.68, 1.34 | | 7/9 |
| **Team-based system navigation model** | | | | | |
| **Dolovich 2016** | I: Health TAPESTRY, volunteer-led home visit to assess health status and goals, action planning with healthcare team including links to community support.  C: Wait-list control (UC) | Physical activity (International Physical Activity Questionnaire) | Total: MD: 0.464 min/week, 95% CI –0.025, 0.953 | | 10/13 |
|  |  |  | Moderate: MD: 0.453 min/week, 95% CI –0.374, 1.280 | |  |
|  |  |  | Vigorous: MD: 0.681 min/week, 95% CI –0.199, 1.561 | |  |
|  |  |  | Moderate or vigorous: MD: 0.689 min/week, 95% CI –0.127, 1.505 | |  |
|  |  |  | **Walking: MD: 1.130 min/week, 95% CI 0.306, 1.953** | |  |
|  |  |  | Sitting time: MD: –0.111 min/week, 95% CI –0.218, 0.005 | |  |
| **Zhang 2018** | I: Older person-centred and integrated health management model programme intervention led by community health centre staff and multidisciplinary care team including self-management, health management, referral to community programs, and family participation.  C: Bimonthly health education | Smoking habits (Smoking history questionnaire, *Non-smoking vs. smoking*) | **Adjusted OR 0.501, 95% CI 0.352, 0.738** | | 4/13 |
|  |  | Diet score (Food frequency questionnaire), *High diet score vs. low diet score* | **Adjusted OR 0.511, 95% CI 0.381, 0.686** | |  |
|  |  | Alcohol intake (Average weekly intake of pure alcohol), *≤350 ml/week vs. ≥350 ml/week* | **Adjusted OR 0.496, 95% CI 0.367, 0.67** | |  |
|  |  | Physical activity (Tool NR), *≥150 min moderate-to-vigorous activity/week vs. ≤150 min moderate-to-vigorous activity/week* | **Adjusted OR 0.417, 95% CI 0.331, 0.525** | |  |
| **PATIENT ACTIVATION, SELF-EFFICACY, AND EMPOWERMENT OUTCOMES (n=5)** | | | | | |
| **Lay person-led system navigation model** | | | | | |
| **Kangovi 2016** | I: Goal setting plus IMPaCT, standardized intervention led by community health workers. Includes tailored coaching, social support, navigation, and advocacy.  C: Goal setting plus UC | Achievement of chronic disease management goal (% Yes) | +1.1%, p = 0.81 | | 9/13 |
|  |  | Patient activation (Patient Activation Measure) | MD: 0.7 (SD NR), p = 0.66 | |  |
| **Kangovi 2018** | I: Goal setting plus IMPaCT, standardized intervention led by community health workers. Includes tailored coaching, social support, navigation, and advocacy.  C: Goal setting plus UC | Patient activation (Patient Activation Measure) | MD: 1.9, 95% CI -0.1, 3.8 | | 11/13 |
| **Loskutova 2016** | I: Cities for Live Program, patient navigators assessed needs, barriers, limitations, stage of change and linked to 2-3 community programs.  C: Baseline | Self-efficacy (Hybrid of Stanford Scale, Diabetes Empowerment Scale, Chronic Illness Resources Survey developed by study team, higher score indicates improved self-efficacy levels) | **T0 (baseline): 3.1, SD 0.8**  **T1 (end-of-study): 3.6, SD 0.7**  **p < 0.001** | | 6/9 |
| **Team-based system navigation model** | | | | | |
| **Dolovich 2016** | I: Health TAPESTRY, volunteer-led home visit to assess health status and goals, action planning with healthcare team including links to community support.  C: Wait-list control (UC) | Goal attainment (based on attainment of individual health goals, using the mean difference of scores between groups from baseline) | Adjusted MD: –1.50, 95% CI –6.5, 3.50 | | 10/13 |
|  |  | Self-efficacy score (Arthritis Self-Efficacy scale) | Adjusted MD: 2.34, 95% CI –2.38, 7.06 | |  |
|  |  | Patient empowerment (CIHI common indicators) | Adjusted MD: 0.172, 95% CI –0.02, 0.36 | |  |
| **Self-navigation with lay support as needed system navigation model** | | | | | |
| **Tung 2020** | I: “HealtheRx” intervention, electronic-medical record generated personalized list of local community resources with access to community health information specialist as needed.  C: UC | Higher category of confidence in finding resources in community to help manage health (single item adapted from Bandura’s self-efficacy measurement), *I vs. C* | **Adjusted OR: 2.1, 95% CI 1.2, 3.6** | | 8/9 |
| Note: C = comparator group, CI = confidence interval, CIHI = Canadian Institute for Health Information, EQ-5D = EuroQol-5D, EQ-5D-5L = EuroQol-5D-5L, EQ-VAS = EuroQol Visual Analogue scale, HADS = Hospital Anxiety and Depression Scale, I = intervention group, IMPaCT = Individualized Management for Patient-Centered Targets, INTERMED-E-SA = INTERMED Elderly Self-Assessment, MD = mean difference, MET = metabolic equivalent, NR = not reported, p = p-value, PPI = posterior probability interval, QoL = quality of life, RAND-36 = RAND 36-Item Health Survey, SD = standard deviation, SF-12 = 12-Item Short Form Survey, SF-36 = 36-Item Short Form Survey, T0 = timepoint 0, T1 = timepoint 1, UC = usual care | | | | | |
