# Supplementary material for: Effectiveness of system navigation programs linking primary care with community-based health and social services: a systematic review
Source: BMC Health Serv Res. 2023 May 8;23:450. doi: 10.1186/s12913-023-09424-5 (PMC10165767; doi:10.1186/s12913-023-09424-5)
Supplement: Supplementary file 7 — Additional file 7. Patient Experience Outcomes. [file 12913_2023_9424_MOESM7_ESM.docx]

# **Additional file 7: Patient Experience Outcomes (n=5)**

| **Study** | **Description of Intervention/Comparator** | **Outcome (Tool)** | **Effect and significance** | **Risk of Bias Score** |
| --- | --- | --- | --- | --- |
| **Lay person-led system navigation model** | | | | |
| **Kangovi 2016** | I: Goal setting plus IMPaCT, standardized intervention led by community health workers. Includes tailored coaching, social support, navigation, and advocacy.  C: Goal setting plus UC | % Patients reporting highest quality of care - comprehensiveness (CAHPS-PCMH) | **+9.5%, p = 0.01** | 9/13 |
|  |  | % Patients reporting highest quality of care - supportiveness of self-management (CAHPS-PCMH) | **+24.9%, p < 0.001** |  |
| **Kangovi 2018** | I: Goal setting plus IMPaCT, standardized intervention led by community health workers. Includes tailored coaching, social support, navigation, and advocacy.  C: Goal setting plus UC | Highest rating for quality of care – comprehensiveness (CAHPS-PCMH), *I vs. C* | **OR: 1.8, 95% CI 1.4, 2.4** | 11/13 |
|  |  | Highest rating for quality of care - supportiveness of self-management (CAHPS-PCMH), *I vs. C* | **OR: 1.8, 95% CI 1.4, 2.4** |  |
| **Health professional-led system navigation model** | | | | |
| **Boult 2013** | I: Nurse-led Guided Care intervention including assessment of patient needs, care-planning and coordination, transitional care, monitoring, self-management, caregiver support and access to community-based services.  C: UC | High quality care – overall (PACIC) *I vs. C* | **OR: 2.19, 95% CI 1.38, 3.49** | 7/13 |
|  |  | High quality care – goal setting (PACIC) *I vs. C* | **OR: 2.35, 95% CI 1.57, 3.52** |  |
|  |  | High quality care – coordination (PACIC) *I vs. C* | **OR: 2.08, 95% CI 1.26, 3.44** |  |
|  |  | High quality care – decision support (PACIC) *I vs. C* | **OR: 1.52, 95% CI 1.11, 2.06** |  |
|  |  | High quality care – problem solving (PACIC) *I vs. C* | **OR: 1.47, 95% CI 1.06, 2.04** |  |
|  |  | High quality care – patient activation (PACIC) *I vs. C* | OR: 1.22, 95% CI 0.87, 1.71 |  |
|  |  | "Very satisfied" with regular health care (PCAS) *I vs. C* | OR: 1.50, 95% CI 0.77, 2.82 |  |
|  |  | "Excellent or very good" access to telephone advice (PCAS) *I vs. C* | **OR: 1.66, 95% CI 1.02, 2.73** |  |
|  |  | "Excellent or very good" wait time for appointment when sick (PCAS) *I vs. C* | OR: 1.09, 95% CI 0.61, 2.04 |  |
|  |  | Access to doctor's appointment the same day when sick (PCAS) *I vs. C* | OR: 1.20, 95% CI 0.65, 2.29 |  |
|  |  | Quality of care - communication (PCAS) | MD: 2.79, 95% CI -0.68, 6.61 |  |
|  |  | Quality of care – integration (PCAS) | MD: 2.97, 95% CI -0.97, 6.60 |  |
| **Vanderboom 2014** | I: Nurse-led Community Connections Program, including strengths assessment, action planning, crisis prevention plan, and circle of support, comprised of community and informal resources for self-management.  C: UC | Quality of care – overall (PACIC) | **MD: 0.50 (SD NR), p = 0.03** | 7/9 |
|  |  | Quality of care – patient activation (PACIC) | MD: 0.24 (SD NR), p = 0.85 |  |
|  |  | Quality of care – delivery system design (PACIC) | MD: 0.52 (SD NR), p = 0.19 |  |
|  |  | Quality of care – goal setting (PACIC) | **MD: 0.79 (SD NR), p = 0.02** |  |
|  |  | Quality of care – problem solving (PACIC) | **MD: 0.74 (SD NR), p = 0.02** |  |
|  |  | Quality of care – coordination (PACIC) | MD: 0.16 (SD NR), p = 0.43 |  |
| **Team-based system navigation model** | | | | |
| **Dolovich 2016** | I: Health TAPESTRY, volunteer-led home visit to assess health status and goals, action planning with healthcare team including links to community support.  C: Wait-list control (UC) | Access to health care resources (CIHI common indicators) *Higher vs. Lower level of difficulty* | OR: 1.17, 95% CI 0.59, 2.32 | 10/13 |
|  |  | Comprehensiveness of health care (CIHI common indicators) | MD: 0.37, 95% CI -0.42, 1.16 |  |
|  |  | Patient-centeredness (CIHI common indicators) | MD: 0.1, 95% CI -0.07, 0.28 |  |
|  |  | Satisfaction with health care (single item; rated from 1 to 10) | MD: -0.11, 95% CI -0.54, 0.31 |  |
| Note: **Bold test indicates significant difference.** C = comparator group, CAHPS-PCMH = Consumer Assessment of Healthcare Providers and Systems-Patient Centered Medical Home, CI = confidence interval, CIHI = Canadian Institute for Health Information, I = intervention group, IMPaCT = Individualized Management for Patient-Centered Targets, MD = mean difference, NR = not reported, NS = not statistically significant, PACIC = Patient Assessment of Chronic Illness Care tool, PCAS = Primary Care Assessment Survey, OR = odds ratio, UC = usual care | | | | |
