# Supplementary material for: Effectiveness of system navigation programs linking primary care with community-based health and social services: a systematic review
Source: BMC Health Serv Res. 2023 May 8;23:450. doi: 10.1186/s12913-023-09424-5 (PMC10165767; doi:10.1186/s12913-023-09424-5)
Supplement: Supplementary file 8 — Additional file 8. Caregiver Outcomes. [file 12913_2023_9424_MOESM8_ESM.docx]

# **Additional file 8: Caregiver Outcomes (n=2)**

| **Study** | **Description of Intervention/Comparator** | **Outcome (Tool)** | **Effect and significance** | **Risk of Bias Score** |
| --- | --- | --- | --- | --- |
| **CAREGIVER EXPERIENCE OUTCOMES (n=2)** | | | | |
| **Health professional-led system navigation model** | | | | |
| **Boult 2013** | I: Nurse-led Guided Care intervention including assessment of patient needs, care-planning and coordination, transitional care, monitoring, self-management, caregiver support and access to community-based services.  C: UC | Caregiver perception of recipients’ quality of care (PACIC) | **Hedges’ d: 0.47, 95% CI 0.15, 0.78** | 7/13 |
|  |  | Caregiver perception of recipients’ quality of care – goal setting (PACIC) | **Hedges’ d: 0.47, 95% CI 0.15, 0.79** |  |
|  |  | Caregiver perception of recipients’ quality of care – coordination (PACIC) | **Hedges’ d: 0.43, 95% CI 0.12, 0.75** |  |
|  |  | Caregiver perception of recipients’ quality of care – decision support (PACIC) | **Hedges’ d: 0.41, 95% CI 0.09, 0.74** |  |
|  |  | Caregiver perception of recipients’ quality of care – problem solving (PACIC) | Hedges’ d: 0.17, 95% CI -0.14, 0.49 |  |
|  |  | Caregiver perception of recipients’ quality of care – patient activation (PACIC) | **Hedges’ d: 0.47, 95% CI 0.14, 0.80** |  |
| **Vanderboom 2014** | I: Nurse-led Community Connections Program, including strengths assessment, action planning, crisis prevention plan, and circle of support, comprised of community and informal resources for self-management.  C: UC | Support persons’ assessment of patient-centered care (Family-Centered Care Self-Assessment) *I vs. C* | Reported “much more” patient-centered care (values NR, NS) | 7/9 |
| **CAREGIVER HEALTH OUTCOMES (n=1)** | | | | |
| **Health professional-led system navigation model** | | | | |
| **Boult 2013** | I: Nurse-led Guided Care intervention including assessment of patient needs, care-planning and coordination, transitional care, monitoring, self-management, caregiver support and access to community-based services.  C: UC | Caregiver strain (Caregiver Strain Index) | Hedges’ d: -0.08, 95% CI -0.37, 0.20 | 7/13 |
|  |  | Caregiver depression (Center for Epidemiological Studies Depression Scale) | Hedges’ d: 0.23, 95% CI -0.06, 0.51 |  |
| Note: **Bold text indicates significant difference.** C = comparator group, CI = confidence interval, I = intervention group, NR = not reported, NS = not statistically significant, PACIC = Patient Assessment of Chronic Illness Care tool, UC = usual care | | | | |
