# Supplementary material for: Effectiveness of system navigation programs linking primary care with community-based health and social services: a systematic review
Source: BMC Health Serv Res. 2023 May 8;23:450. doi: 10.1186/s12913-023-09424-5 (PMC10165767; doi:10.1186/s12913-023-09424-5)
Supplement: Supplementary file 9 — Additional file 9. Cost-Related Outcomes. [file 12913_2023_9424_MOESM9_ESM.docx]

# **Additional File 9: Cost-Related Outcomes (n=2)**

| **Study** | **Description of Intervention/Comparator** | **Outcome (Tool)** | **Effect and significance** | **Risk of Bias Score** |
| --- | --- | --- | --- | --- |
| **Lay person-led system navigation model** | | | | |
| **Dye 2018** | I: Volunteer health coach intervention, including needs assessment, home visits, self-management, education on use of self-monitoring equipment, linking to external services based on client needs.  C: Matched patients who chose not to participate | ED/hospital visit costs per patient (patient records) | I: Median $4865 USD (IQR $17,191)  C: Median $7112 USD (IQR $19,451), descriptive statistics only | 4/9 |
|  |  | Cost of emergent care per patient, for participants who received emergent care for at least one diagnosis of cerebrovascular disease, congestive heart failure, or diabetes mellitus (patient records) | I: Mean $11,161 USD (SD NR)  C: Mean $21,523.68 USD (SD NR), descriptive statistics only |  |
| **Mayhew 2009** | I: Integrated Care Coordination Service led by a care coordinator, includes identification of needs and liking to relevant health, social security or other organizations.  C: Baseline | Projected annual cost savings from changes in A&E attendance following intervention (modelled based on 500 cases/year and estimated cost of 200 pounds per A&E visit) | Projection: -282 thousand pounds (lower bound) to -781 thousand pounds (upper bound) | 8/9 |
|  |  | Projected annual cost savings from changes in bed-days following intervention (modelled based on 500 cases/year and average cost of 300 pounds per bed-day) | Projection: -2.13 million pounds (lower bound) to -4.31 million pounds (upper bound) |  |
| Note: A&E = Accident and Emergency, C = comparator group, ED = emergency department, I = intervention group, IQR = interquartile range, NR = not reported, SD = standard deviation, USD = US dollars | | | | |
